# Mechanosensory entities and functionality of endothelial cells

**DOI:** 10.3389/fcell.2024.1446452

**Published:** 2024-10-23

**Authors:** Claudia Tanja Mierke

**Affiliations:** Faculty of Physics and Earth System Science, Peter Debye Institute of Soft Matter Physics, Biological Physics Division, Leipzig University, Leipzig, Germany

**Keywords:** mechanoreceptors, ion channels, integrins, endothelial cell anisotropy, fluid shear stress, forces, vascular function, stiffness

## Abstract

The endothelial cells of the blood circulation are exposed to hemodynamic forces, such as cyclic strain, hydrostatic forces, and shear stress caused by the blood fluid’s frictional force. Endothelial cells perceive mechanical forces via mechanosensors and thus elicit physiological reactions such as alterations in vessel width. The mechanosensors considered comprise ion channels, structures linked to the plasma membrane, cytoskeletal spectrin scaffold, mechanoreceptors, and junctional proteins. This review focuses on endothelial mechanosensors and how they alter the vascular functions of endothelial cells. The current state of knowledge on the dysregulation of endothelial mechanosensitivity in disease is briefly presented. The interplay in mechanical perception between endothelial cells and vascular smooth muscle cells is briefly outlined. Finally, future research avenues are highlighted, which are necessary to overcome existing limitations.

## 1 Introduction

Endothelial cells are subject of mechanical cues from their microenvironment. They form a continuous monolayer at the inner side of vessels, and thus, they are located at the interface of the blood flow and the vessel wall. This specific location of endothelial cells enables them to sense mechanical cues, such as forces that can alter endothelial cell functions. Moreover, due to the blood flow–vessel wall interface location, endothelial cells can serve as mechanical transducers for neighboring cells, such as vascular smooth muscle cells (VSMCs). In turn, from a mechanical viewpoint, VSMCs may act as regulators of the endothelial cell shape and function. Apart from VSMCs, the morphology and functions of endothelial cells are mainly controlled by the blood pressure and extracellular matrix (ECM). The intricate hemodynamic milieu exposes mechanical signals toward endothelial cells in a direct manner, such as tensile forces. These tensile forces strain endothelial cells (Raupach et al., 2007) and strengthen their cell–cell junctions. These mechanical cues are converted into biochemical messages and determine numerous facets of cell fate and determinacy, ranging from endothelial cell proliferation, differentiation, motility, adhesiveness, cell death, and survival. The hemodynamic shear stress has been revealed as a major and relevant determinant of endothelial functionality and shape. Arterial shear stress above 15 dyne/cm^2^ causes endothelial dormancy and an atheroprotective gene expression pattern. In contrast, a low shear stress of less than 4 dynes/cm^2^ is predominant at atherosclerosis-prone locations and induces an atherogenic phenotype. In large arteries of healthy individuals, average wall shear rates of 80–400 s^-1^ and maximum wall shear rates of 900–1,600 s^-1^ have been determined. Major veins have a larger diameter and less fluid velocity, which leads to lower shear rates (Stroev et al., 2007; Tinken et al., 2009). Likewise, the average shear stress is usually higher in the arterial system, which is between 4 and 70 dynes/cm^2^, than in the venous system, which ranges from 1 to 6 dynes/cm^2^ (Malek et al., 1999). Pathological vessels suffering from stenosis exhibit abrupt alterations in vessel diameter, which can modify the normal flow profile and generate shear stresses of over 1,000 dynes/cm^2^ (Strony et al., 1993). Even though smaller vessels demonstrate reduced fluid velocities, the reduction in the diameter of the vessel is accompanied by an escalation in shear stress and shear rate. *In vivo* determinations of maximum and average shear rates in 6–12-µm precapillary arteries yielded values of 733–6,562 s^-1^ and 587–3,515 s^-1^, respectively (Koutsiaris et al., 2013; Sakariassen et al., 2015).

In adults, maintaining normal blood pressure is also critical for the sustenance of vascular structure and functionality. The pulsatile characteristic of blood pressure produces radial and axial forces, mainly in the shape of cyclic dilation and shear flow, which have a lasting impact on the make-up of the vessel walls so that high blood pressure causes thickening and stiffening of the arterial walls (Laurent and Boutouyrie, 2020; Humphrey, 2021). The friction generated between the blood and the vessel wall produces fluid shear stresses running parallel to the vessel surface, acting primarily on the endothelial cells at the vessel wall boundary (Chiu and Chien, 2011; Roux et al., 2020); circumferential stresses act vertically to the vessel wall on all cells in the vessel wall, such as endothelial cells and VSMCs (Davis et al., 2023; Katoh, 2023).

**FIGURE 1:**
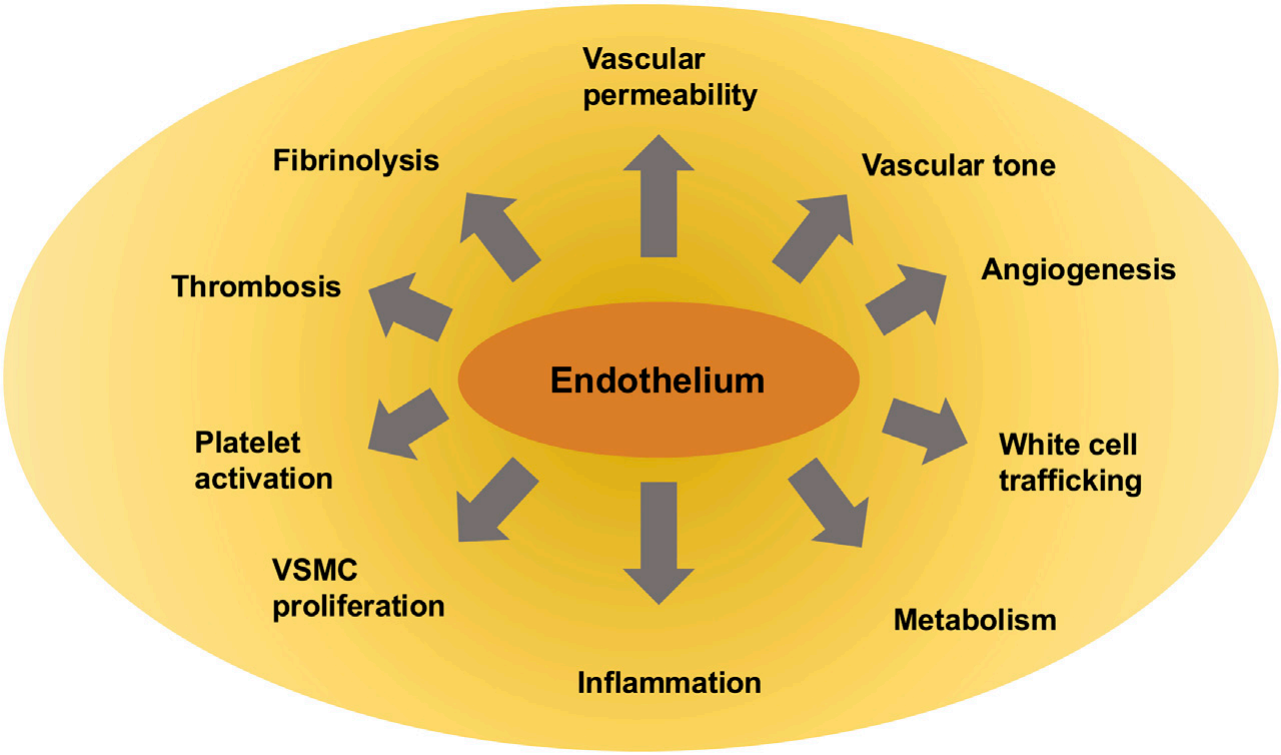
Various endothelial cell functions in the vascular system.

Endothelial cells sense mechanical forces through specific molecules or structures referred to as mechanosensors. Mechanosensors are first responses to alterations in the mechanical surroundings, and many of them are located at the cell’s plasma membrane. Mechanosensors can transduce extracellular mechanical cues into intracellular chemical signals and initiate signaling pathways, following the engagement of adapter molecules. The process involved is known as mechanotransduction, a mechanism consisting of a series of signaling processes triggered by mechanical impulses. Apart from the activation of mechanosensors, physical forces impact the fluidity of the plasma membrane and cause alterations in protein assemblies on the plasma membrane, which is associated with changes in intercellular junctions, cell–ECM connectivity, cytoskeletal remodeling, and, subsequently, transcriptional reactions in sculpting certain cell phenotypes. The endothelium can perceive these mechanical signals and assimilate inputs from various kinds of physical stimuli, which is of vital importance for the control of vascular functions. The functions of endothelial cells lie in the production of messenger molecules that regulate vascular tone, vascular flow, activity of immune cells, and adhesion, which are all involved in the maintenance of blood pressure and perfusion (Félétou and Vanhoutte, 2006; Moncada and Higgs, 2006). These various endothelial functions are depicted in Figure 1 and also comprise regulation of overall vessel integrity, angiogenesis, hemostasis, vascular growth, vessel restructuring, and growth of tissues, as well as metabolism.

Endothelial function homeostasis is prevalent under physiological conditions, whereby the endothelium keeps an equilibrium between vasodilation and vasoconstriction, impairment and fostering of the migration and proliferation of VSMCs, and fibrinolysis and thrombogenesis, as well as hindering and encouraging the adhesion and platelet agglomeration (Risman et al., 2023). Endothelium-originated molecules with vasodilatory and antiproliferative actions comprise endothelium-derived hyperpolarizing factor (EDHF) (Chen et al., 1988), nitric oxide (NO) (Garty and Palmer, 1997), and prostacyclin (PGI2) (Moncada et al., 1976), whereas endothelin-1 (ET-1) (Yanagisawa et al., 1988), angiotensin II, and reactive oxygen species (ROS) belong to the factors that enhance vasoconstrictive processes (Endemann and Schiffrin, 2004; Just et al., 2008). Endothelial cells also secrete antithrombotic molecules such as NO and PGI2, both of which suppress platelet aggregation, and prothrombotic molecules, such as the von Willebrand factor that fosters the aggregation of platelets and plasminogen activator inhibitor-1 (PAI-1) that blocks fibrinolysis (Risman et al., 2023). In contrast, the dysregulation of homeostasis is tethered to pathological conditions, comprising atherosclerosis, hypertension, cancer metastasis, and diabetes (hampered endothelium-based vasodilation).

In mechanobiological research, the endothelium has been found to be predestined for mechanical analyses because of its appearance as a mostly closed monolayer, the internal apical–basal polarity of endothelial cells, the barrier function of the endothelium, the facilitation of transmigration of immune cells and cancer cells, angiogenesis, and the ease of stimulation. Several mechanosensory elements have been identified, and it has been proposed that the perception of mechanical signals enables the endothelial cells to mechanically analyze their environment and react to changes. There is still much to be done in the field of physical characterization of individual endothelial cells and the perception of physical changes in the environment by endothelial cells regarding their function. The key goals of this review article are, first, to introduce the mechanical environment of endothelial cells lining blood vessels. Second, the most important mechanosensory proteins on endothelial cells are presented. Third, the prominent membrane structures serving as mechanosensors are discussed. Fourth, the coupling between mechanosensation and endothelial cell functions is highlighted. Fifth, the deregulation of the mechanosensory system in pathological conditions is discussed. Sixth, future research directions in the fields of physiology and pathology are predicted.

## 2 Physical environment to which the endothelial cells in the blood vessels are exposed

It is important to describe which biomechanical forces are relevant for endothelial function and possibly for dysfunction, e.g., endothelial cells lining the lumen of different types of (mostly distensible) vessels. The physical environment of the endothelium consists of a series of interconnected stresses (for definitions see Box 1) that fit into two general classes. The blood vessels are, therefore, not exposed to hydrostatic pressure as such but to the distension that accompanies a rise in (blood) pressure, which stresses the contacts between the neighboring endothelial cells. Hydrostatic pressure is the amount of pressure that a fluid column, e.g., blood, creates when it is trapped in blood vessels or heart chambers. The circulatory system depends on hydrostatic pressure to govern the blood flow and ensure optimized perfusion of organs and tissues. The hydrostatic pressure inside the blood vessels fluctuates according to parameters such as the position of the vascular tree and the level of the pathological disorder. Gravity increases the hydrostatic pressure in the lower body limbs. In the case of pathological states such as high blood pressure, the hydrostatic pressure can rise and result in endothelial malfunction and damage to the blood vessels (Weenink and Wingelaar, 2021). The intensity of this force is several orders of magnitude higher than the shear stress to which only the endothelial cells (but not other cells inside the vessel wall) are subjected. This is the most physiologically pertinent biomechanical force for endothelial cells, which possess an augmentation mechanism at their cell–cell junctions to perceive it more effectively. Most notably, it controls the synthesis of endothelial NO (Vozzi et al., 2014). NO is a highly relevant mediator that mainly governs the phenotype of the endothelial cells themselves, both in the microcirculation in which unidirectional shear stress is mostly dictated by narrowing of the lumen caused by vasoconstriction and at bifurcations or bends of large conduit arteries, in which it declines and undergoes oscillatory behavior, leading to atherogenesis.

Vascular endothelial cells are affected *in vivo* by two different hemodynamic forces: cyclic strain due to the distension (dilatation) of the vessel wall caused by transmural pressure and shear stress, and the frictional force created through blood flow (Ballermann et al., 1998). Shear stress operates at the apical cell surface to distort cells in the blood flow direction; wall stretch tends to distort cells in all orientations (Ballermann et al., 1998). The response to shear stress is at least partially distinct from the reaction to cyclic elongation, implying that elongation of the cytoskeleton by itself is not sufficient to account for it. Acute shear stress *in vitro* causes fast cytoskeletal reorganization and activates endothelial cell signaling pathways, leading to acute liberation of NO and prostacyclin, activation of transcription factors, such as nuclear factor (NF)κB, c-fos, Nrf2, c-Jun, and SP-1 (Chien et al., 1998; Wilson et al., 2000; Dai et al., 2007; Hahn et al., 2009). Thereby, the transcription of genes, comprising ICAM-1, VCAM-1, MCP-1, tissue factor, platelet-derived growth factor-B (PDGF-B), transforming growth factor (TGF)-β1, cyclooxygenase-II, and endothelial nitric oxide synthase (eNOS), is activated (Wilcox et al., 1988; Nakashima et al., 1998; Viñals and Pouysségur, 2001; Chien, 2003; Tang et al., 2014; Tran et al., 2022). This type of reaction thus has analogies with the reactions of endothelial cells reacting to inflammatory cytokines (Wautier and Wautier, 2021). In sharp distinction, endothelial cells adjust to chronic shear stress through reshaping and thinning their architecture to mitigate shear stress (Zhong et al., 2020). These bordered cells adhere strongly to their substrate and exhibit indications of cell differentiation (Gifre-Renom et al., 2022). The elevated adhesion, following chronic shear stress, has been artificially generated to produce vascular grafts with confluent endothelial cell monolayers that are better preserved post-implantation *in vivo*, thereby surmounting a principal hurdle to endothelization of vascular grafts (Dardik et al., 1999). In the following, the cyclical strain and the shear stress are defined by physical equations.

### 2.1 Cyclical strain

Cyclic strain, especially in arteries and heart valves, is the periodic distortion of blood vessels due to pulsatile blood flow. The vessel wall is cyclically dilated and relaxed because of this mechanical stress, which triggers a reaction in the endothelial cells (Katoh, 2023). The blood flow triggered by myocardial spasm and relaxation generates cyclic tension on the walls of the arteries. The magnitude and length of cyclic stretch are determined by blood pressure, diameter of the vessel, and compliance (Brozovich et al., 2016). Cyclic stress has been found to impact the proliferation of endothelial cells, migration, and the liberation of vasoactive molecules. It has also been implicated in vascular smooth muscle cell attachment, inflammation, restructuring of the ECM, and expression of genes (transcriptional profile). For example, stretch-activated ion channels, focal adhesions and focal adhesion complexes, and integrins are representative mechanosensitive proteins that transmit the reaction of arterial endothelial cells toward cyclic stretching (Baeyens et al., 2014; Zhang et al., 2020).

*In vivo*, endothelial cells are subjected to two principal hemodynamic forces: transmural pressure gradients and wall shear stress. In blood vessels *in vivo*, these forces fluctuate in a pulsatile manner, apart from those points where the pressure curve of the heart has been attenuated due to high upstream resistances. Transmural pressure gradients cause vessel wall distention and generate the emergence of wall tension (T), as provided in Equation 1 using Laplace’s law:

T=∆P⋅r, (1)

where ∆P denotes the transmural pressure gradient and r stands for the vessel radius. The wall tension is, therefore, in direct proportion to the transmural pressure gradient and the diameter of the vessel. Transmural pressure gradients lead to the development of wall tension, which causes the cells to elongate and deform in all directions. The vessel wall’s distensibility is dictated through its thickness, its constitution, and the extent of contraction of smooth muscle cells (Katoh, 2023). Since wall tension increases with the growing vessel diameter, for a given transmural pressure gradient, tension is maximized in vessels characterized as highly distensible. The tension propagates throughout all structural constituents of the vessel wall, incorporating the extracellular connective tissue and the cytoskeleton of the cells that it is composed of. The tension is transferred to the cell cytoskeleton at the sites of cell–cell and cell–matrix attachment, at which the cytoskeleton links to adhesion molecules on adjacent cells or to matrix moieties through various transmembrane proteins. In highly distensible vessels, the extent of cell cytoskeleton deformation and tension resulting from transmural pressure gradients may be extremely pronounced. Wall shear stress exerts its strong vascular actions not through a mechanical effect on the vascular structure itself but solely by initiating biological signaling and is several orders of magnitude lower than other mechanical stresses acting on the coronary arteries, such as tensile or compressive stresses (Gijsen et al., 2019).

BOX 1 **Definitions of mechanical terms**

**Axial stress** = This refers to a normal stress that is positioned parallel to the symmetrical axis of the cylinder.

**Circumferential stress** (or **hoop stress**) = This is a normal stress in the tangential (azimuthal) orientation.

**Longitudial stress** = The axial force places the vessel either in tension or in compression.

**Radial stress** = It represents a stress that acts toward or away from the central axis of a construction element.

**Shear stress** = It refers to the tangential stress resulting from the friction of a fluid flowing alongside a solid interface.

**Tensile stress** = It is produced by the blood pressure, has a circumferential spread, and influences all elements of the vessel wall. It is directly proportional to the transmural pressure (P) and radius (r) and reciprocally related to the vessel wall thickness (w).

**Transmural pressure** = This is defined as the pressure difference between the intravascular pressure and the pressure applied to the external surface of the vessel wall.

**Wall shear stress** = It is the resistance that the flowing blood places on the vessel wall, i.e., the force applied by the blood movement against the arterial wall. This stress is assumed to have an important effect on the adaptive processes of the vascular wall by triggering the release of substances such as nitric oxide (NO), prostacyclin, and endothelin from the endothelial cells.

**Vasoconstriction** = It refers to the narrowing of blood vessels due to the tightening (constriction) of blood vessels, typically occurring when the muscles of the blood vessel walls contract, thereby reducing the vessel lumen. It is the opposite phenomenon of vasodilation.

**Vasodilation** = It is a process that occurs when the lumen of the blood vessels is enlarged by the muscles of the blood vessel walls expanding.

### 2.2 Shear stress

Endothelial cells are also exposed to the frictional force created by the shear force of blood rushing along their apical sites. This force is based on the mean fluid flow velocity, its viscosity, and the physical geometry of the blood vessels. For Newtonian fluids, which are defined as fluids for which the flow velocity has no influence on the viscosity, flowing in stiff vessels with constant internal geometry, uniform gradients of velocity arise so that the fluid velocity is lowest at the fixed vessel wall and increases with rising distance therefrom. A liquid flow with this typical uniform velocity gradient is referred to as laminar flow as the liquid can be considered a sequence of molecular layers (laminae) that slide along one another with accelerating velocity as one comes closer to the middle of the vessel. In a uniform, stiff cylinder, the shear stress ( τ ) across the vessel wall based on Poiseuille’s law can be deduced as provided in Equation 2:

$$\tau =4Q\cdot \frac{\eta }{\Pi }\cdot r^{3},$$ (2)

whereby Q stands for the fluid flow rate and η provides fluid viscosity. At a steady mean flow velocity, the larger the flow resistance, the higher the shear stress, owing to either increased fluid viscosity or decreased vessel diameter. Shear stress induces a deformation of the cell (elongation) that increases the tension of the cytoskeleton, even though the orientation of the deformation is distinct from that generated by transmural pressure gradients. In the strict meaning of the word, shear stress and transmural pressure gradients are, therefore, unconnected forces that both exert strain on the endothelial cells. In both situations, deformation of cytoskeletal elements, cell membranes, and cell–cell and cell–matrix adhesion sites occurs, leading to tension generation. With shear stress, the force acts on a particular cell in a single direction only, whereas stretching occurs in all directions because of transmural pressure gradients. It is also noteworthy that it is solely the endothelial cells but not vascular smooth muscle cells or pericytes that are subjected to shear stress, while all vessel wall elements are distorted through transmural pressure gradients. Thus, the endothelial cell seems to play a unique part in sensing various mechanical cues.

### 2.3 *In vivo* shear stress

Biological systems deviate from the conditions defined in the previous section because the blood fluid is not a Newtonian fluid, i.e., the viscosity of the blood drops with rising speed, and the vessels are non-uniform, differently expandable containers (Katoh, 2023). At extremely low blood speeds, the aggregation of cell elements leads to a significant increase in blood viscosity, while the viscosity at very high speeds is just four times higher than that of water (Letcher et al., 1981). Even though the blood flow in vessels with a diameter of less than 0.5 mm is generally laminar, it deviates somewhat from this characteristic in very small vessels like the glomerular capillaries. Moreover, the shear stress is affected by transmural pressure gradients as vessel distention enlarges the vessel diameter and thus leads to a tendency to reduce the shear stress. The impact of vessel distention on shear stress can be substantial since shear stress is in inverse relation to the third power of the radius of the vessel. Differences in vessel wall folds and cell structure also lead to fluctuations in shear stress at various points within a single vessel (Satcher et al., 1992) and even on the single cell (Satcher et al., 1992). Wall shear stress is estimated at a variety of sites in the circulation (Giddens et al., 1993; Jones et al., 1997); some have considered circulating cellular components and the structure and distensibility of the vessel. Therefore, the analysis of the wall shear stress has been standardized later (Gijsen et al., 2019). The mean shear stress is usually at its lowest in the large veins, typically below 1 dynes/cm^2^. It is generally greatest in small arterioles, in which it can range from 60 to 80 dynes/cm^2^. It is remarkable that the mean shear stress in small venules is also high due to the large flow rates and the small diameter of these vessels (20–40 dynes/cm^2^) (Lipowsky et al., 1978). Human cervical artery bifurcation observations indicate that the curvature and shape of the vessel can influence wall shear stress in a dramatic fashion, with values varying from less than 1 dyne/cm^2^ to over 600 dyne/cm^2^ at various locations in the same vessel (Zarins et al., 1983). Shear stress in glomerular capillaries has been estimated with a computer modeling approach and varies from approximately 1 to approximately 95 dynes/cm^2^, with mean stress levels of 5–20 dynes/cm^2^ in the majority of circuits (Remuzzi et al., 1992).

### 2.4 Shear stress in different types of vessels

The shear stress is exerted on the endothelial cells through the blood circulation. It has a major impact on both vascular physiology and the functioning of endothelial cells. The impact of shear stress in arteries and its involvement in regulating endothelial cell response is reviewed in this section (Chien, 2007). The aorta and other large arteries experience laminar shear stress, which is characterized by the unidirectional blood flow of restricted spatial and temporal variations (Tarbell and Pahakis, 2006). Arterial shear stress, which fluctuates across the vascular system, is fundamental to the sustenance of endothelial functioning and integrity. Laminar shear stress is a condition with unidirectional flow and exhibits moderate temporal and spatial variations and occurs in large arteries like the aorta. The permeability of the endothelium is influenced, the expression of endothelial adhesion molecules is adjusted, and the formation of vasodilators, such as NO, is activated, whereas thrombosis and inflammation of the endothelium are suppressed (Cunningham and Gotlieb, 2005; Chiu and Chien, 2011). Mechanosensitive gene expression linked to vascular reorganization and evolution of atherosclerosis is impacted through the pulsatile and bidirectional flow in mid-sized arteries (Khan et al., 2021). Smaller arterioles have a branching and twisting shape, which leads to perturbed or fluctuating flow characteristics. Varying shear stress profiles in arteries impact endothelial cell physiology and gene expression in different ways (Cunningham and Gotlieb, 2005; Tarbell and Pahakis, 2006; Chiu and Chien, 2011). Capillaries are essential for exchanging nutrients and for perfusing the tissue. They are exposed to a lower shear stress compared to larger arteries. The shear stress in capillaries is generally at a minimum and exhibits considerable spatial and temporal fluctuations due to the fluctuating spread of the blood flow (Pries et al., 2000). The form and orientation of endothelial cells and the development of fenestrations in capillaries are altered when subjected to shear stress. Moreover, shear stress affects the formation of carriers that support the exchange of nutrients and angiogenesis. The sensitive interplay of shear stress and endothelial reactions within the capillaries is fundamental for homeostasis of the tissue and maintaining optimal microvascular functionality (Chien, 2007). A typical vessel’s shear stresses are the following: aorta ranges from 1 to 22 dyn/cm^2^ (Cheng C. P. et al., 2003), arteries are in the range of 10–70 dyn/cm2 (Cheng C. et al., 2003), veins are between 1 and 6 dyn/cm^2^ (Malek et al., 1999), and capillaries exhibit 3–95 dyn/cm^2^ (Koutsiaris et al., 2013).

Proliferation of endothelial and vascular smooth muscle cells takes place in reaction to a rise in axial stress due to arterial stretching, while shear stress and circumferential stretching stay undisturbed. Enhanced matrix metalloproteinase MMP activity (a family of zinc-dependent extracellular matrix proteins) and the buildup of ECM accompany this process, which leads to growth in length to compensate the effect and re-establish normal axial tension (Liu and Khalil, 2017; Wang and Khalil, 2018; Shimoda, 2019). In laminar flow conditions, the shear stress is dictated by parameters such as blood viscosity, flow speed, and the diameter of the vessel. The shear stress rises with declining vessel diameter if viscosity and speed are kept at a constant level. When blood vessels are exposed to elevated shear stress, they tend to dilate to accommodate and revert to their normal state. In theory, vessels are capable of unlimited expansion and remodeling within the boundaries of the body if this leads to a normalization of circumferential stretching and shear stress. Nevertheless, the mechanical adjustment capability of elastin and collagen within the vascular wall is restricted (Hoefer et al., 2013).

The wall tension is directly correlated with the blood pressure, particularly the pulse pressure. Blood pressure applies three forms of stress to the arterial wall: the longitudinal stress, the radial (or normal) stress (vertically to the vessel axis), and the tangential (or hoop) stress (for definitions see Box 1). The shear stress operates parallel to the surfaces of the intima, media, and adventitia sheets and has the effect that one sheet glides over the other sheet. The strain is the degree of deformation per initial material length and is linked to the stress. Shear stress is able to injure the vessel wall by causing harm to the intima, which is the inner vessel layer, as blood flows over the surface, and it is now considered that this is the gateway for the incorporation of plasma lipids into the wall, causing atherosclerosis (Wang S. et al., 2016). The effect of shear stress does not halt at the intima. Shear stress may alter the inner sheets of a multilayer artery so that one layer is displaced in relation to the other as a function of blood pressure and the varying characteristics of the individual layers (Mishani et al., 2021). Alterations in mechanical forces, like tensile or shear stresses, lead to adaptations in the vessel wall architecture to compensate for the altered circumstances and eventually bring the tensile and shear stresses back to their former values (Gimbrone et al., 1999; Cui et al., 2020). Temporary alterations in vessel diameter are caused by sudden alterations in mechanical stress. These alterations are predominantly governed by the liberation of vasoactive stimulants or alterations in myogenic tone. Conversely, chronic alterations lead to considerable alterations in the form and configuration of the vessel wall (Renna et al., 2013; Martinez-Quinones et al., 2018). This process is termed vascular restructuring, which is a term that refers to alterations in vessels exposed to mechanical forces. For instance, experimental hypertension results in an augmentation of wall thickness in resistance arterioles and arteries because of VSMC hyperplasia and in conduit arteries because of hypertrophy. In the same way, decreased mechanical stress results in vascular atrophy (Lehoux and Tedgui, 1998).

### 2.5 Vascular tone

Vascular tone refers to the extent to which the blood vessel walls, especially the arteries and arterioles, constrict or relax. Vasoconstriction means the narrowing (constriction) of blood vessels by vascular smooth muscle cells within the vessel walls. If the blood vessels narrow, the blood flow is reduced or obstructed. The relation to NOS (nitric oxide synthase) consists in its function as an essential controller of vascular tone. NO produced by endothelial cells fulfills a key function in governing the acute dilation of arteries that takes place when the blood flow rises inside these vessels (Rubanyi et al., 1986). Shear stress demonstrably enhances nitric oxide production by activating endothelial NO synthase and increasing its gene expression (Boo and Jo, 2003). Shear stress is also proven to activate NO synthesis in cultured endothelial cells (Ohno et al., 1993). Tetrahydrobiopterin, an essential cofactor of eNOS, and intracellular Ca^2+^ concentration rise in reaction to shear stress, and the activation of protein kinases switches on eNOS (Corson et al., 1996; Fleming et al., 1998). NF-κB, a shear stress response component in the promoter of the *eNOS* gene, and 3′-polyadenylation help in stabilizing eNOS mRNA to improve transcription in reaction to shear stress (Uematsu et al., 1995; Weber et al., 2005). Endothelial cells subjected to shear stress are also more likely to generate the potential vasodilators prostacyclin, adrenomedullin, and C-type natriuretic peptide (Chun et al., 1997). The generation of endothelin and the enzyme expression, which turns angiotensin into the powerful vasoconstrictor angiotensin II, are both reduced in reaction to shear stress (Rieder et al., 1997).

### 2.6 Endothelial barrier function

The endothelial barrier ensures the retention of vascular integrity and the transfer of substances between the bloodstream and the adjacent tissue. The function of the endothelium as a barrier can be perturbed through mechanical stresses, including tension, hydrostatic pressure, and shear stress (Cahill and Redmond, 2016). The intactness of the endothelial barrier is mostly sustained by two distinct kinds of junctional complexes, adherens junctions which offer cell–cell adhesion and provide mechanical stability, and tight junctions which serve as a physical border restricting paracellular permeability (Adil et al., 2021). Mechanical stress can influence the endothelial barrier capacity via the regulation of the production and breakdown of junctional complexes. For instance, shear stress encourages the maturation and growth of the tight junctions, which leads to increased barrier strength. In turn, cyclic stress causes harm to the proteins that comprise the tight junctions, thereby enhancing permeability (Gulino-Debrac, 2013). Mechanical stress impacts the permeability of endothelia; shear stress, in particular, reduces permeability through encouraging the creation of tight junctions and decreasing the expression of adhesion molecules (Sukriti et al., 2014; Claesson-Welsh et al., 2021). Inversely, excessive shear stress or prolonged cyclic stress can enhance permeability and interfere with the endothelial barrier. Alterations in the junctional proteins, the arrangement of the cytoskeleton, and the endothelial glycocalyx contribute to these irregularities (Sukriti et al., 2014; Schött et al., 2016; Claesson-Welsh et al., 2021). Studying the processes that control endothelial barrier function during mechanical stress is essential to gain an insight into vascular diseases characterized mainly due to enhanced permeability. This has previously been initiated by exerting shear stress on two kinds of endothelial cells, such as human aortic endothelial cells (HAECs) and human umbilical vein endothelial cells (HUVECs). Shear stress application increases the typical characteristic manifestation of a mature endothelium, which shows a linear arrangement of VE-cadherin at the cell–cell interface and a redistribution of actin filaments alongside the periphery of the endothelial cells (Silvani et al., 2021). An escalating sequence of ascending force levels, varying from 186 pN to 3.5 nN, has then been utilized in a single measurement to assess the force-dependent apparent stiffness of the membrane cortex in the kPa regime, indicating that the membrane cortex has become stiffer and can exert increased forces. Moreover, it has been found that the beads adhered to cells grown in dynamic conditions were more difficult to dislodge using acoustic force spectroscopy than those grown in static culture, indicating a more rigid membrane cortex located at the periphery of the cell. Based on the successful acoustic force spectroscopic measurements, it can be concluded that this biophysical method can be used in the future to determine changes in cell mechanics based on force measurements on adherent cells under conditions that mimic their native microenvironment and, thus, also to uncover the shear stress relationship of the mechanical characteristics of adjacent endothelial cells (Silvani et al., 2021).

### 2.7 Endothelial nitric oxide synthase (eNOS) and nitric oxide (NO) generation

**FIGURE 2:**
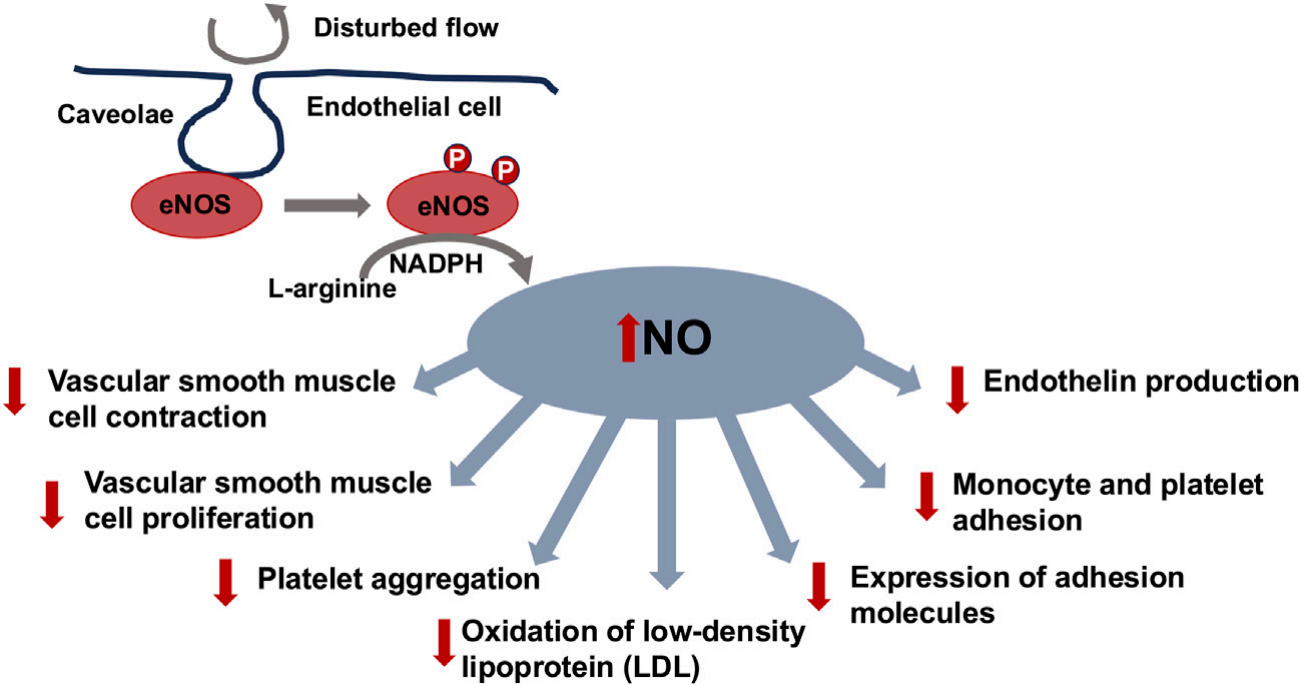
Endothelial nitric oxide synthetase (eNOS)-generated nitric oxide (NO) provides atheroprotective characteristics.

Activity of eNOS and NO generation in endothelial cells is governed through mechanical stress, specifically shear stress. NO, which is a powerful vasodilator and signal molecule with numerous physiological roles (Figure 2), is generated upon shear stress activation of eNOS (Li et al., 2004; Sriram et al., 2016). The phosphorylation of eNOS at certain locations, which is triggered via shear stress, leads to the generation of NO because of its activation. NO is a key actor in maintaining vascular homeostasis. NO is produced in endothelial cells through eNOS upon converting L-arginine to L-citrulline (Vallance and Chan, 2001). It is predominantly secreted from endothelial cells in reaction to shear stress triggered by circulating blood or receptor-regulated compounds like acetylcholine, bradykinin, or serotonin (Boulanger and Lüscher, 1990). NO efficiently dissipates to the VSMCs and stimulates soluble guanylate cyclase (sGC), leading to elevated levels of cyclic guanosine-3,5-monophosphate (cGMP) and VSMC relaxation (Joannides et al., 1995; Vallance, 2001). In addition, NO also hinders the adhesion and migration of leukocytes, the proliferation of VSMCs, and the adhesion and agglomeration of platelets, and counteracts apoptosis and inflammation, which has an altogether anti-atherogenic impact (Wheatcroft et al., 2003). The natural half-life of NO is extremely short (under 4 s). It is quickly broken down into nitrite and subsequently nitrate, prior to being removed from the body via urine (Moncada and Higgs, 2006). At the same time, NO can also be an endocrine vasoregulator that regulates the flow of blood in the microcirculation (Datta et al., 2004). The key point is that decreased eNOS expression and/or NO bioavailability is linked to dysfunction of the endothelium (Oemar et al., 1998; Sena et al., 2008). When NO penetrates the smooth muscle cells of the vascular system, it causes them to relax and dilate. This helps control the vascular tone, the flow of blood, and the retention of endothelial functionality (Boo et al., 2002; Sriram et al., 2016). Moreover, NO exhibits multiple favorable actions on endothelial functioning, which includes anti-inflammatory and anti-thrombotic effects. It reduces the formation of adhesion molecules, inhibits platelet agglomeration, and inhibits the adhesion of leukocytes to endothelial cells (Kubes et al., 1991; Riddell and Owen, 1997; Liao, 2013; Gao et al., 2018). NO has additional impacts on the motility, angiogenesis, and proliferation of endothelial cells (Pilard et al., 2022). NO represents a signal transduction molecule that exhibits various functions in physiological events including vasodilation (relaxation of blood vessels), neurotransmission, and immune reactions (Ziegler et al., 1998; Balligand et al., 2009; Boycott et al., 2020; Uchida et al., 2021).

eNOS is induced through different agonists and shear stress by a multitude of cellular mechanisms, which includes elevated intracellular Ca^2+^, engagement with substrates and cofactors, protein phosphorylation, engagement with adapter and regulatory proteins, and inter-subcellular domain shuttling (Fleming, 2010). Certain serine and threonine residues of eNOS have been phosphorylated through PKA and Akt. Activation of eNOS relies on its degree of phosphorylation. eNOS usually exists as a monomer and is in an inactive form. The dimerization of eNOS is evoked through phosphorylation and the attachment of calcium–calmodulin, which leads to the generation of an active enzyme (Fleming and Busse, 1999). Mechanical forces can induce the stimulation of tyrosine kinases, which are enzymes that attach phosphate groups directly to tyrosine residues. The activity of NOS or other associated molecules can be influenced through subsequent signaling routes that are potentially induced. Moreover, NO can influence cellular events that affect tyrosine phosphorylation since NO is able to react with specific molecules to produce nitrosylated derivatives that can interfere with protein functioning (Gantner et al., 2020; Pourbagher-Shahri et al., 2021; Lundberg and Weitzberg, 2022). Importantly, the relation between NOS, mechanoresponsiveness, and tyrosine phosphorylation can be impacted from the environmental milieu and may potentially engage intricate signaling cascades that are still under exploration.

## 3 Mechanosensors of endothelial cells

Research into the pathways underpinning endothelial cell mechanotransduction has advanced remarkably. Several molecules located in the apical, junctional, and basal regions of endothelial cells have been found to be crucial for endothelial cell mechanotransduction. Therefore, an overview of the different mechanosensory elements is given and discussed below.

The groups of Chalfie and coworkers and Patapoutian and coworkers (Árnadóttir and Chalfie, 2010; Syeda et al., 2016) identified the following criteria for a well-functioning mechanosensitive ion channel, which had to meet stringent criteria to ensure that the channel is a direct and not an indirect mechanosensor.

I. The channel is designed to incorporate a pore-forming subunit that enables fast ion conduction.
II. When the purified channel is reformed in an artificial, cell-free lipid bilayer, it is expected to open in response to tension exerted on the bilayer.
III. Site-directed mutagenesis of key channel domains that influence pore selectivity or conductivity is expected to modify mechanosensitivity.
IV. Forced expression of the channel in a non-mechanosensitive cell is expected to provide mechanosensitive.
V. Both the gene and the protein of the channel need to be expressed in the presumably mechanosensitive cell.
VI. Genetic deletion of the channel is intended to eliminate mechanosensitivity in a manner that excludes the possibility that the channel plays solely a developmental function or is a downstream signaling partner of another mechanosensor. Genetic deletion, nevertheless, can perturb normal signaling complexes, resulting in off-target events, so expression of a dominant-negative (dead) channel construct might be an improved option.

Finally, whether these criteria are sufficient to distinguish true mechanosensitive channels from those that are indirect mechanosensors is a matter of debate.

**FIGURE 3:**
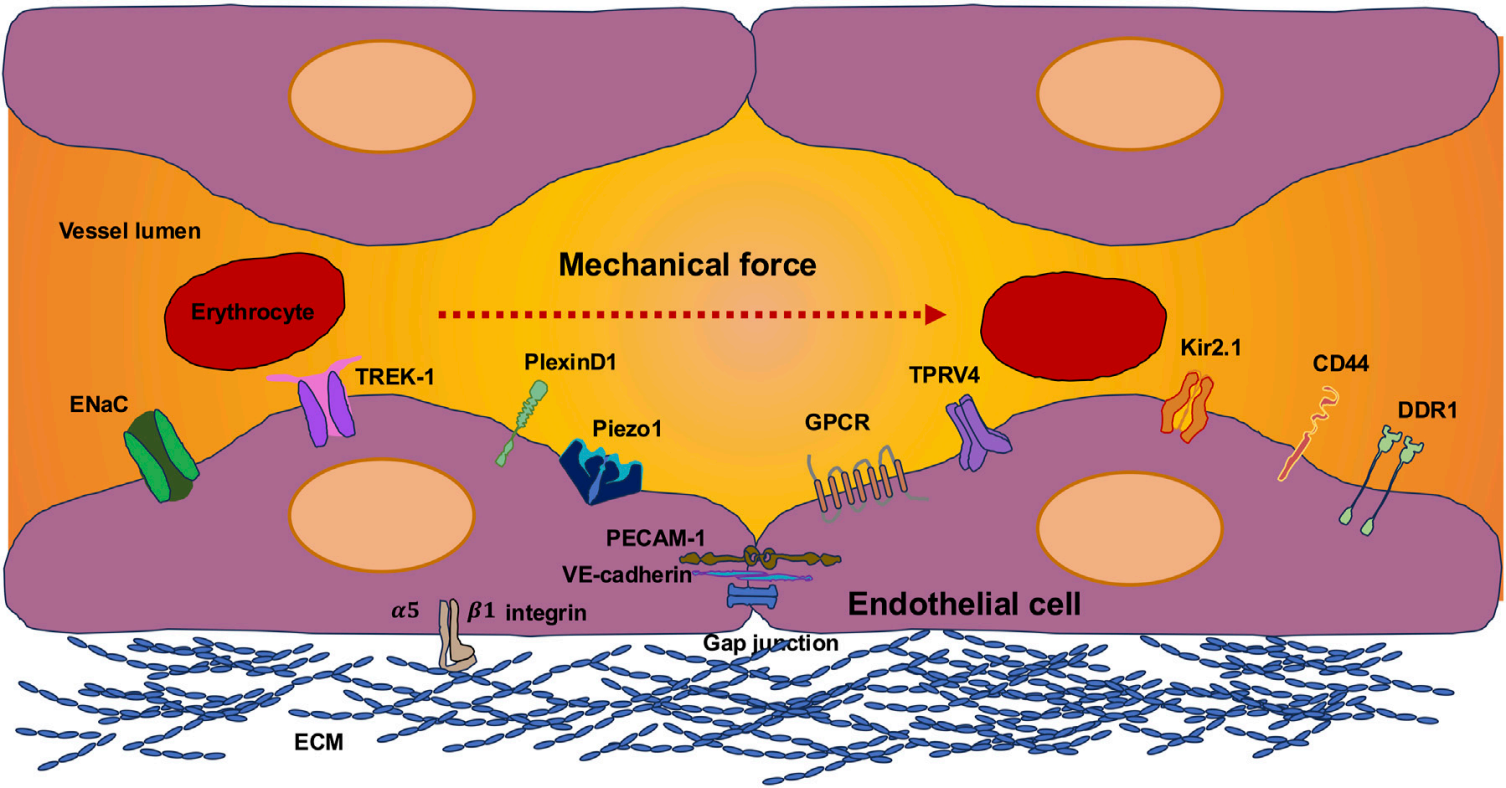
Types of endothelial mechanoreceptors at their luminal site are permanently subject to vascular mechanical forces. The sensing of these forces can be performed through specific mechanosensors. Among them are ion channels, such as Piezo1, transient receptor potential vanilloid 4 channel (TRPV4), K^+^ channel inwardly rectifying 2.1 (Kir 2.1), epithelial sodium channel (ENaC), TREK-1, DDR1, CD44, and mechanoreceptors, comprising the G-protein-coupled receptor (GPCR).

The translation of shear forces into biological inputs is facilitated through mechanosensors that can be either specialized intercellular compartments or protein assemblies. These particularly comprise intercellular junctional complexes, integrins, the cytoskeletal components, specific ion channels, G-proteins and G-protein-coupled receptors (GPCRs), the cellular glycocalyx, caveolae, primary cilia (if present on mammalian endothelial cells), and plexin D1 (PLXND1) (Figure 3) (Chatterjee and Fisher, 2014; Mehta et al., 2020). In the case of stimulation, the transmission of the mechanical force commences at the neighboring cell circumference and then spreads across the entire cell (Vogel and Sheetz, 2006). Several signal paths arranged after the mechanosensors are triggered at almost the same time.

Messenger substances like cytosolic calcium (Ca^2+^) and NO are subsequently produced, and the mechanical transduction of nuclear transcription factors becomes enabled. Such shear stress-responsive transcription factors comprise Krüppel-like factor 2 (KLF2) and KLF4, nuclear factor erythroid 2-like (NRF2), and Yes-associated protein (YAP)/transcriptional coactivator with a PDZ-binding domain (TAZ) (Tsaryk et al., 2022). KLF2, which is expressed in vascular endothelial cells of mouse embryos even at E9.5, is triggered through atheroprotective streaming (Dekker et al., 2002) and controls nuclear gene transcription (Dekker et al., 2005). Nevertheless, endothelial cells in arteries with perturbed flow exhibited, amongst other characteristics, an accumulation of TEAD1 and ETV3 tethering sites for transcription factors (Andueza et al., 2020). Shear stress-responsive elements have been found in endothelial cells that are susceptible to atheroporotic oscillatory shear stress. Their activation of the YAP/TAZ signaling pathway was implicated in facilitating the endothelial cell reaction to this mode of shear stress (Bondareva et al., 2019).

In the study of histone changes in reaction to pulsatile shear stress, KLF4 was recognized as a cognate downstream transcription factor (He et al., 2019). In addition, the early growth response (EGR1) and SMAD1/5 are involved in the control of shear stress-induced alterations in gene expression (Khachigian et al., 1997; Zhou J. et al., 2012), whereas the transcription factor Snail reacts to low shear stress and facilitates the switch from an endothelial to a mesenchymal state (Mahmoud et al., 2017). Twist transcription factor is also expressed at low shear stress (Mahmoud et al., 2016). Consequently, the comprehension of transcriptional organization of the shear stress reaction is by no means exhaustive and could imply the involvement of different groups of transcription factors based on the flow scheme and/or endothelial cell type studied (Nakajima and Mochizuki, 2017).

The diverse kinds of mechanosensors can be broadly grouped into three major types, such as apical mechanosensors, cell–cell junctions, and cell-matrix interactions. Apical mechanosensors comprise mechanosensitive ion channels, including PIEZO (Barakat et al., 1999; Yamamoto et al., 2000; Ranade et al., 2014; Wang et al., 2020; Cudmore and Santana, 2022), primary cilia (Iomini et al., 2004; Hierck et al., 2008), the glycocalyx—like glypican-1, heparan sulfate, hyaluronan (HA), and sialic acid—(Weinbaum et al., 2003; Foote et al., 2022), GTP-binding proteins (Gudi et al., 2003; Iomini et al., 2004), and the caveolae (Boyd et al., 2003; Shihata et al., 2016). There are convergent roles of Piezo1 action as, on one hand, Piezo1-driven Akt activity triggers vasodilation and vasorelaxation due to increased flow conditions and, on the other hand, conveys in vasoconstriction (Porto Ribeiro et al., 2022). The cooperation between these two mechanisms is presumably key to controlling the blood pressure. In cell–cell contacts, platelet endothelial adhesion molecule-1 (PECAM-1), vascular endothelial cadherin (VE-cadherin), and vascular endothelial growth factor receptors (VEGFRs), such as VEGFR2, have been demonstrated to constitute an intricate mechanosensory system (Osawa et al., 2002; Tzima et al., 2005; Chiu et al., 2008). PECAM-1 can perceive unidirectional and perturbed flow and consequently transmit protecting and inflammatory messages. Therefore, PECAM-1 knockouts exhibit an intriguing phenotype consisting of amplified lesion formation at locations of the unidirectional flow and diminished lesion development at locations of the perturbed flow (Goel et al., 2008; Harry et al., 2008). In contrast, syndecan-4, which belongs to transmembrane heparan sulfate proteoglycans, is necessary for aligning endothelial cells during shear stress but not required for other mechanoreactions, pointing to a function in flow direction recognition (Baeyens et al., 2014). Depletion of syndecan-4 leads to atherosclerotic plaque formation.

There are important and comprehensive review articles on cell–cell junctions acting as mechanosensors (Dorland and Huveneers, 2017; Angulo-Urarte et al., 2020), but nonetheless, some main cell–cell adhesion receptors are discussed in this review. The major class of mechanosensory receptors comprises ion channels, and thus, they are presented and discussed in the following.

### 3.1 Ion channels

Several classes of ion channels operate under the control of ligands or are voltage-activated, whereas others are mechanically activated. Mechanosensitive ion channels can be directly activated through physiological mechanical forces. Because of the vital importance of endothelial cells, the question of how the endothelium perceives and reacts to blood flow and the accompanying mechanical forces is something that has been of great concern to scientists for decades. The first proof of a mechanosensitive endothelial channel that transmits the mechanically triggered cation inflow occurred over 40 years ago (Guharay and Sachs, 1984). This groundbreaking revelation was soon afterward followed by the finding of shear stress-activated K^+^ currents within endothelial cells (Olesen et al., 1988).

Endothelial mechanosensory channel activity varies in direct reaction to shear forces, while the activity of other channels can be modified by forces in an indirect way. For an ion channel to be regarded as a genuine mechanosensory channel, several criteria must be satisfied. First, channel overexpression in a null cell needs to impart mechanosensitivity, and removal or blocking of the channel needs to abrogate the mechanoreaction. The second is that the signal and the protein expression of the channel need to be detectable in the mechanosensitive cell—here the endothelial cell. The third is that the alleged mechanosensitive channel needs to have a direct part in ion permeation as a force reaction. Fourth, the expression of channels is essential to the mechanosensing mechanism and cannot be a downstream objective of any other mechanosensing mechanism. Lastly, the targeted mutagenesis of the ion channel needs to change the mechanoresponse.

Mechanosensitive ion channels are an entrenched group of biological molecules that have an exceptional ability to perceive and handle external mechanical stimuli by triggering an electrical or chemical response that is ultimately incorporated into a coherent cellular reaction (Brohawn, 2015). Several ion channels, such as the ion channels TREK-1 (Honoré, 2007) and TRPC1 (Maroto et al., 2005), are found to be triggered directly by the lipid bilayer during force propagation with no additional associated cellular constituents, such as the cytoskeleton. This corresponds to a kind of gating model that is commonly seen in bacterial ion channels and is referred to as the bilayer mechanism. In sharp distinction, the overwhelming majority of mechanosensitive ion channels present in the membranes of animal cells, which comprise multiple TRP channels (Kanzaki et al., 1999; Liman, 2007; Nilius et al., 2007; Pedersen and Nilius, 2007; Zhu et al., 2011), are controlled through a process referred to as the tethered mechanism. The tethered mechanism exerts a force on them indirectly through the cytoskeleton via ECM molecules like fibronectin and auxiliary scaffold constituents (Rohács et al., 1999; Matsuda et al., 2003). As a multitude of physical stimuli act on the plasma membrane, they are transmitted via the ECM toward the cytoskeleton, which is referred to as the dual tether model, by activating overlapping integrin receptors situated at focal adhesions (Geiger et al., 2009). Consequently, cytoskeletal actin and other structural elements may represent pivotal pathways of cellular force transduction that distinctly affect the mechanosensitivity of ion channels.

The opening of mechanosensitive ion channels, such as Piezo1, triggered upon shear stress or flow-induced membrane elongation constitutes the initial phase of mechanosignaling (Christensen and Corey, 2007; Murthy et al., 2017; Martino et al., 2018). These ion channels exhibit opposing characteristics sometimes, varying from hyperpolarization by K^+^-selective TREK channels to depolarization through Ca^2+^- and Na^+^-permeable Piezo1 channels. The primary area of focus was on mechanosensitive cation-permeable ion channels, causing Ca^2+^ entry in the endothelial cell. Ample evidence indicates that the increase in intracellular Ca^2+^ occurs as a first step in reaction to shear stress. In the endothelium, augmented Ca^2+^ is followed by the activation of eNOS and Ca^2+^-activated intermediate conductance K^+^ channels (IKCa), all of which leads to vasodilation via eNOS-driven NO liberation and possibly hyperpolarization of the plasma membrane. Ion channels appear to be exceptionally skilled at sensing forces, and it is widely assumed that they perform a pivotal task in the perception of shear stress (Hyman et al., 2017).

Transient receptor potential (TRP) channels operate as non-voltage-driven cation channels that are controlled by a variety of stimuli and are associated with a wide range of cellular processes (Nilius and Owsianik, 2011). There are, at minimum, 10 different TRP channels (TRPC1, 5, 6; TRPV1, 2, 4; TRPM3, 7; TRPA1; and TRPP2) that are suggested to act mechanosensitive (Tobin et al., 2002; Sidi et al., 2003; Christensen and Corey, 2007; Inoue et al., 2009b; Malsch et al., 2014). TRP can moderate Ca^2+^ transmission, although it may be regulated by direct Ca^2+^ engagement with the channel or by the Ca^2+^–calmodulin complex-driven activation mechanism. The inward Ca^2+^ current from TRP channels in VSMCs results in the depolarization of the plasma membrane and a compelled inward current via voltage-gated Ca^2+^ channels, comprising L-type or T-type Ca^2+^ channels, CaV1.2/CaV3.1. The Ca^2+^–calmodulin compound triggers myosin light-chain kinase and starts the contractile response (Hill-Eubanks et al., 2014). TRPV1 can exercise an anti-inflammatory impact on endothelial cells through the activation of eNOS over the eNOS/NO signaling route (Wang Y. et al., 2017). In the last two decades, there have been major advances in research on TRP channels, including their involvement in diseases and their function as mechanosensors (Zhang et al., 2023). The subsequent mechanisms have been proposed: first, direct activation through extracellular forces including membrane elongation and shear-induced alterations of the lipid bilayer structure and consequent distortion of channel domains (Gao et al., 2003; Maroto et al., 2005; Spassova et al., 2006); second, the engagement of ion channel architecture with cellular or matrix constituents including ECM, intracellular cytoskeleton, or proteins (Becker et al., 2009; Matthews et al., 2010); and third, secondary activation through other well-defined primary mechanosensors and their consequent biochemical conversion into effector TRP channels (Köhler et al., 2006; Loot et al., 2008; y Schnitzler et al., 2008). In the following, after the discussion of Piezo1, specific mechanosensitive receptor types of the TRP family are also presented and discussed, including TRPV4, TRPC1, TRPC6, TRPM7, TRPP2, and ENaC.

#### 3.1.1 Piezo1

Piezo1 and Piezo2 constitute mechanically excited cation channels that function as large homomultimeric assemblies capable of facilitating cation fluxes in multiple tissues (Coste et al., 2015; Volkers et al., 2015). Both isoforms function as mechanically driven gates and convey non-selective Na^+^, K^+^, and Ca^2+^ ion flows characterized by rapid activation kinetics. However, Piezo2 is mainly expressed in tactile epithelial cells (Merkel) (Woo et al., 2014) and mechanosensory nerve cells (Murthy et al., 2017). Piezo1 can convey mechanically derived flows in different cell types, comprising endothelial cells and VSMCs. (Ranade et al., 2014; Retailleau et al., 2015). Piezo1 protein represents a trimeric, propeller-shaped channel protein consisting of a central anchor, three long rods, and three blade-like structures (Zhao et al., 2019).

Ion channels respond extremely sensitively toward mechanical forces and change their conformation when mechanically excited (Martinac, 2004). The most well-characterized ion channel in endothelial shear stress reactions is Piezo1, which is necessary for shear stress-imposed endothelial orientation, Ca^2+^ influx, and reshaping of focal adhesions in reaction to the flow (Li et al., 2014). Both the stable/laminar and disturbed flow trigger a signaling route through Piezo1 and the GPCRs Gq/G11 and P2Y2 (P2RY2) (Albarrán-Juárez et al., 2018). When the flow is disturbed, this route activates the mechanosensory complex and integrins, thereby initiating NFκB activity and inflammation, which ultimately leads to atherosclerosis. In addition, Piezo1 has been shown in zebrafish embryos to react to mechanical forces in heart valve development, where it induces endothelial expression of the transcription factor KLF2 (Duchemin et al., 2019). Piezo1 also acts in the flow-induced mitochondrial signaling route, resulting in the activation of ERK and upregulation of KLF2 (Coon et al., 2022). Intriguingly, Piezo1 associates with PECAM1, the Ca^2+^ influx, and dynamical remodeling of the actin cytoskeleton under flow (Chuntharpursat-Bon et al., 2023).

VE-cadherin at the endothelial cell–cell junction, where it maintains Ca^2+^, is a bone-fide mechanosensitive ion channel. Piezo1 is a true mechanosensory since it fulfills the criteria as such. Piezo1 acts as a non-selective cation channel, which is somewhat better permeable for Ca^2+^ compared to Na^+^. Electrophysiological and imaging investigations on endothelial cells derived from several vascular beds revealed that activation of Piezo1 results in cationic currents and intracellular Ca^2+^ signaling (Porto Ribeiro et al., 2022). Activation of Piezo1 can be accomplished in experiments by mechanical (for example shear stress) or chemical activation, such as by Yoda1—which is a selective Piezo1 activator (Servin-Vences et al., 2017). The impact of the endothelial cell Yoda1 imitates shear stress-induced reactions and is mitigated by inhibitors of mechanically gated channels (Wang S. et al., 2016).

Piezo1 has been found to be an effective sensor for shear forces in endothelial cells and is implicated in the orientation of the endothelial cells in the direction of the flow (Li et al., 2014). Endothelial Ca^2+^ signaling fulfills a critical task in the development of the vasculature. Therefore, after the identification of Piezo1 in endothelial cells, previous endeavors were focused on comprehending the impact of Piezo1-mediated Ca^2+^ transients on vessel evolution. Piezo1 also activates multiple Ca^2+^-dependent endothelial metalloproteinases implicated in the process of angiogenesis (Ichioka et al., 1997; Zhou et al., 2000; Kang et al., 2019). Piezo1-based Ca^2+^ events regulate wayfinding and cerebrovascular organization within brain endothelial cells (Liu T. et al., 2020). Collectively, these investigations have revealed that Piezo1-faciliated Ca^2+^ signaling is necessary for vascular evolution, patterning development, and angiogenesis. The activation of Ca^2+^-dependent enzymes is essential for the three-dimensional arrangement and orientation of endothelial cells throughout developmental processes. Ca^2+^-activated protease calpain, which cleaves focal adhesion proteins necessary for cellular orientation, becomes upregulated with the activation of Piezo1 and is critical during vessel evolution (Lai et al., 2022). Piezo1 activation has been found to be important for stress fiber orientation and the alignment of endothelial cells (Ranade et al., 2014). Laminar flow-based activation of Piezo1 induces the flow-triggered liberation of ATP from endothelial cells, leading to Gq/G11-coupled purinergic P2Y2 receptor engagement (Wang et al., 2015; Wang S et al., 2016). P2Y2 receptor and Gq/G11 pathways cause the activation of AKT and eNOS and facilitate flow-based vasodilation. Laminar and perturbed currents equally activate the identical early mechanosignaling route that integrates Piezo1- and Gq/G11-based paths (Albarrán-Juárez et al., 2018). Consequently, the Piezo1 channel activator Yoda1 triggers NO-driven relaxation of intrapulmonary arteries of mice (Lhomme et al., 2019; Porto Ribeiro et al., 2022). Moreover, in cultured microvascular endothelial cells, Piezo1 channel activation induced, either by shear stress or by the chemical agonist Yoda1, a disintegrin and metalloproteinase domain-containing protein 10 (ADAM10), which is a Ca^2+^-regulated transmembrane sheddase that facilitates S2-Notch1 modification by cleavage. In accordance with this finding, a Piezo1-induced abundance enhancement of the Notch1 intracellular domain (NICD) has been identified, which relies on ADAM10 and the subsequent S3 cleavage enzyme, namely, γ-secretase. Conditional endothelial-specific silencing of Piezo1 of adult mice repressed the expression of several Notch1 target genes in the liver vasculature, implying a constitutive functional importance of Piezo1 *in vivo*. Together, the data imply that Piezo1 functions as a mechanism that imparts force responsiveness to ADAM10 and Notch1, with subsequent implications for the persistent activation of Notch1 target genes and possibly related processes. Ca^2+^ translocations trigger subsequent signaling cascades and stimulate Ca^2+^-based endothelial mechanisms that control vascular diameter guidance (Caolo et al., 2020).

A pivotal mechanism enabled through Ca^2+^ signals involves the synthesis of the powerful vasodilator NO. Flow-mediated vasodilation is due to Piezo1-driven Ca^2+^ inward current and Ca^2+^-driven eNOS activation, NO formation, and ultimately relaxation of VSMC. Endothelial-cell specific loss of NO-driven vasodilation in lead to hypertension in Piezo1-knockout mice (Wang S. et al., 2016). In addition, the downregulation of Piezo1 leads to a reduction in renin and thus to blood pressure control in the kidney (Yang et al., 2022). Conditional endothelial cell-specific ablation of Piezo1 in adult mice resulted in a reduction in physical fitness (Bartoli et al., 2022). Apoptosis of muscle microvascular endothelial cells and capillary dilution were apparent and enough to account for the impact on performance. Selective high regulation of thrombospondin-2 (TSP2), which triggers apoptosis of endothelial cells, occurred without affecting TSP1, which is a cognate key actor in the physiology of muscle. In muscle endothelial cells, TSP2 was barely expressed but strongly expressed in muscle pericytes, where NO suppressed the *Tsp2* gene without affecting Tsp1. In endothelial cells, Piezo1 was necessary for the normal endothelial NOS expression. The findings point to a cooperative partnership between endothelial cells and muscle pericytes, where the endothelial Piezo1 perceives the blood flow to preserve the capillary density and thus preserve the physical performance (Bartoli et al., 2022).

Nevertheless, Piezo1-driven vasodilation in several vascular patches is facilitated through NO generation (Li et al., 2014; Evans et al., 2018; John et al., 2018; Lhomme et al., 2019). Endothelial Piezo1 has been demonstrated to affect the activity of GPCRs. Laminar flow triggers Piezo1 activation and facilitates the liberation of adenosine triphosphate (ATP), which then stimulates the activation of purinergic Gq/11 PCRs to finally enhance NO generation (Albarrán-Juárez et al., 2018). Specifically, Gq-PCR activity is a key controller of ion channels in endothelial cells (Harraz et al., 2018a; Harraz et al., 2018a), but it is uncertain whether Gq-PCR signal transduction impacts endothelial Piezo1 activity.

The cation inward current connected with activation of Piezo1 causes depolarization of cells, such as endothelial cells (Rode et al., 2017; Ye et al., 2022). Endothelial cells are linked with VSMCs in structural and electrical terms through myo-endothelial protrusions and gap junctions (Yamamoto et al., 1999; Wu et al., 2006). This electrical pairing eases endothelial cell communication with adjacent VSMCs and probably facilitates the propagation of Piezo1-facilitated electrical cues from endothelial cells to VSMCs. Evidence indicates that Piezo1-triggered endothelial cell depolarization spills over to VSMCs and triggers the activation voltage-gated Ca^2+^ channels that induce vasoconstriction of the mesenteric artery (Rode et al., 2017). This accounts for the reported flow-mediated vasoconstriction rather than vasodilation and the complete cessation of this reaction in endothelial cells from Piezo1-knockout mice. These results, nonetheless, are in contrast to other investigations, demonstrating that blood flow-triggered vasodilation relies on NO and is mitigated in endothelial cells of Piezo1-knockout mice (Li et al., 2014). These disparities need to be examined more closely and may be partly attributable to differing expression profiles in the various arterial branches or to variations between the mouse models. It is also an open question whether Piezo1 stimulates Ca^2+^-activated ion channels within endothelial cells. When this is the case, Piezo1 activity potentially hyperpolarizes or depolarizes the V_m_ of endothelial cells through the activation of Ca^2+^-activated K^+^ or Cl^−^ channels, which is a theory that needs to be verified experimentally. In general, Piezo1 is an essential mechanosensory mechanism within endothelial cells. Two envisionable potential scenarios exist for the prospective regulatory actions of capillary Piezo1 channels in the cerebral blood flow (CBF) (Harraz et al., 2022). In the first case, mechanical forces cause Piezo1-driven Ca^2+^ signaling, which might influence CBF at a local level through the production of NO and consequent relaxation of mural cells, such as pericytes or VSMCs. In the second place, Piezo1, acting as a non-selective cation channel, might convey a depolarizing conductance within capillary endothelial cells. From a conceptual perspective, hyperemia-induced Piezo1 activation potentially depolarizes capillary endothelial cells, which is an action that is proposed to be a built-in restraint system that enables membrane potential restoration, thus aiding the repetitive function of the previously described hyperpolarization-based NVC mechanism (Longden et al., 2017). The two outlined mechanisms are not strictly mutually independent; they may occur in parallel and control CBF by spatially and temporally different mechanisms. In confirmation of this hypothesis, the Ca^2+^/Na^+^-permeable TRPV4 channel within the cerebral capillaries appears to fulfill two tasks: control of membrane potential (Harraz et al., 2018b) and facilitation of Ca^2+^ signal transduction (Dabertrand et al., 2021). It remains unclear as to whether and how Piezo1 channels control CBF and, therefore, needs experimental modification of Piezo1 with simultaneous surveillance of CBF, which is a key aspect under investigation.

Piezo1 channels open as a reaction to a variety of mechanical cues, such as physical membrane deformations that regulate the curvature of flexible domains known as blades. It has been revealed that flow-induced blade movements are functionally coupled to the pore and that at least two widely separated blade regions distinguish flow from two other stimuli, indicating that Piezo1 utilizes different mechanisms to perceive a wide spectrum of mechanical stimuli (Ozkan et al., 2022). An uncommon characteristic of Piezo1 is the highly curved blade, which allows the protein to locally distort the membrane to form a dome shape (Guo and MacKinnon, 2017). When the channel opens, the enclosing membrane is flattened, which expands the dome into an in-plane configuration (Guo and MacKinnon, 2017). This specific event produces free energy, which could act as a cause for the mechanical gating of Piezo1 (Guo and MacKinnon, 2017). Membrane dome hypothesis is a piece of evidence that piezoelectric channels obey the force-from-lipid (FFL) model, which proposes that mechanosensitive channels are directly manipulated through variations from nearby membrane curvature and tension (Martinac and Kung, 2022). A further model that can account for the sensitivity of piezo channels to wide-ranging mechanical cues is the force-from-filament (FFF) model, which enables full-cell mechanosensing across the cytoskeleton (Jiang et al., 2021; Chuang and Chen, 2022). In fact, piezo channels act as a physical connection to the actin cytoskeleton through the cadherin–β-catenin–vinculin complex, and the Cap domain of Piezo1 also interfaces in a direct manner with the extracellular domain of E-cadherin (Wang et al., 2022b) and possibly also VE-cadherin in a similar way (Chuntharpursat-Bon et al., 2019). In living cells, Piezo1 is able to exploit the FFF model in concert with the FFL model, allowing them to act as multipurpose and adaptable mechanotransducers (Jiang et al., 2021). Apart from the activation of the plasma membrane or the filaments of the cytoskeleton, it was found that the activation of Piezo by the ligand Yoda1 is independent of the presence of TRPV4 (Harraz et al., 2022), all of which leads to the hypothesis that Piezo also possibly fulfills a prominent and unique function in mechanosensation and mechanotransductions. The lack of specific and selective inhibitors, given that the molecular structure and ligand binding mechanism are not fully elucidated, is a major challenge in Piezo1 research. Progress has recently been achieved in the design of novel selective inhibitory substance of the Piezo1 channel (Thien et al., 2024). The discovery of inhibitors, although, is so far in its fledgling stages.

#### 3.1.2 TRPV4

TRPV4 was first recognized in the endothelium in 2002 (Watanabe et al., 2002). TRPV4 has since been detected in endothelial cells of various vascular sites, including carotid arteries, mesenteric resistance arteries, pulmonary arteries, arterioles of the skeletal muscle, and cerebral capillaries (Köhler et al., 2006; Hartmannsgruber et al., 2007; Mendoza et al., 2010; Earley and Brayden, 2015). TRPV4 acts as a non-selective cation channel, which is better permeable for Ca^2+^ compared to Na^+^ ions (Liedtke et al., 2000; Strotmann et al., 2000). The endothelial TRPV4 channel participates in multiple vascular processes, which includes control of vascular tone, endothelial cell alignment, angiogenesis, and perfusion (Earley and Brayden, 2015; White et al., 2016). TRPV4 appears to be a prospective receptor for the molecular blood flow sensor that triggers flow-based vasodilation, which is a reaction to elevated blood fluid speed or enhanced blood fluid viscosity. In agreement, TRPV4 has been found to be activated during hypertonic circumstances and membrane elongation induced through cell swelling (Liedtke et al., 2000). The TRPV4 channel reacts sensitively to forces and is essential for recognizing shear stress in endothelial cells (Köhler et al., 2006; Hartmannsgruber et al., 2007; Mendoza et al., 2010). Whether TRPV4 is a genuine mechanosensor is, nonetheless, controversial. Cell-attached patch-clamp approaches failed to activate TRPV4 directly through pipette suction, indicating its indirect activation via force-driven signaling pathways (Earley and Brayden, 2015). Using the rat carotid artery, it has been demonstrated that activation of TRPV4 triggered by endothelial cell agonists or shear stress causes expansion of the rat gracilis arteries. The impairment of eNOS mitigates the TRPV4-driven response (Köhler et al., 2006). In addition, TRPV4 knockouts exhibited markedly decreased flow-driven vasodilation (Hartmannsgruber et al., 2007). In this context, TRPV4-driven relaxation has been identified engaging NO and EDHFs, as well as Ca^2+^ inward flow across endothelial TRPV4 channels in reaction to flux (Mendoza et al., 2010). Thus, direct activation of the channel by mechanical forces has not yet been clearly identified, which indicates that TRPV4 may not be a true mechanosensor (Liedtke et al., 2000; Loukin et al., 2010; Servin-Vences et al., 2017; Nikolaev et al., 2019). Beyond this, the fact that TRPV4 can be stimulated in a physiological manner through endogenous compounds like arachidonic acid (AA) and epoxyeicosatrienoic acids (EETs), which are liberated as a reaction to shear stress, would account for how TRPV4 activity rises with force (Watanabe et al., 2003; Köhler et al., 2006; Loot et al., 2008). In this sense, Piezo1 activation has been found to induce AA metabolite formation in tissue-engineered endothelial cells, which ultimately increases the activity of TRPV4 (Swain and Liddle, 2021). These results imply that Piezo1 functions as the mechanosensor and TRPV4 is functionally downstream to augment and maintain the mechanically generated Ca^2+^ inward current.

Endothelial TRPV4 activation causes vasodilation via NO liberation and/or by changing the V_m_ of endothelial cells. These two regulatory principles are not inherently mutually independent and potentially synergistic. Strongly localized Ca^2+^ effects during the activation of TRPV4 channels, referred to as TRPV4 sparklets, are linked to, first, the activation of eNOS, NO generation, and vasodilation, or second, the engagement of Ca^2+^-activated K^+^ channels and subsequent endothelial cell hyperpolarization (Köhler et al., 2006; Hartmannsgruber et al., 2007; Mendoza et al., 2010; Sonkusare et al., 2012; Longden et al., 2021). Genetic ablation of TRPV4 causes dampened endothelial Ca^2+^ reactions and impaired NO liberation (McFarland et al., 2020). The enhancement of TRPV4-driven Ca^2+^ signal transduction is achieved by sensitization of the inositol 1,4,5-trisphosphate receptor (IP3R) (Heathcote et al., 2019).

TRPV4 channels engage with a variety of proteins and molecules, including proteins and molecules that are manipulated directly through mechanical forces. Similar to various other TRP channels, TRPV4 undergoes activation, following GqPCR induction. Activation of endothelial GqPCR increases the activity of phospholipase C (PLC) and the hydrolytic breakdown of phosphatidylinositol 4,5-bisphosphate (PIP2) into IP3, which results in the liberation of Ca^2+^ from intracellular storage, and diacylglycerol (DAG), inducing protein kinase C (PKC) activation. GqPCR signal transduction in endothelial cells triggers the activation of TRPV4 channels in a PKC- or PIP2-dependent mode (Sonkusare et al., 2014; Harraz et al., 2018b), and IP3/IP3 R signal transduction enhances the activity of TRPV4 (Heathcote et al., 2019). Since some GPCRs exhibit mechanosensitivity (Xu et al., 2018) (see section below), the activation of these GPCRs through forces could increase endothelial cell TRPV4 activity, prompting the hypothesis that TRPV4 channels are integral mechanosensors. Notably, GqPCR signal transduction and subsequent regulation of ion channels like TRPV4 and Piezo1 may represent an important interface between various mechanosensing mechanisms. CAV1, a scaffold protein, directly engages with TRPV4 and colocalizes with gap junction proteins to promote electrical pairing. In fact, CAV1 is necessary for the endothelial Ca^2+^ inward flow and consequent vasodilation (Saliez et al., 2008; Rath et al., 2012). Overall, there is strong support that caveolae are critical microdomains participating in the activity of TRPV4 and vasodilation. In addition to mechanosensing and flow-induced vasodilation, there are several other TRP channels that are key for signal transmission in endothelial cells. For instance, the cerebral endothelial transient receptor potential ankyrin 1 channel (TRPA1) is part of the neurovascular connection, and the activation of TRPA1 through reactive oxygen species causes vasodilation (Sullivan et al., 2015; Thakore et al., 2021).

Shear stress also results in exocytosis-facilitated enrollment of TRPV4 channels and mechanical stress awareness of the endothelium (Baratchi et al., 2016). TRPV4 can colocalize with TRPC1 proteins in endothelial cells derived from rabbit mesenteric arteries. High external Ca^2+^-based endothelial cell-driven vasodilation exhibited TRPV4- and TRPC1-driven Ca^2+^ affluence and NO production stimulation. Activation of TRPV4-based triggered NO formation and consecutive vasodilation could be impeded with N(ω)-nitro-L-arginine methyl ester (L-NAME), which is an eNOS inhibitory substance, the TRPC1 antagonist T1E3, which is an inhibitory peptide or TRPV4 antagonist RN1734. Heteromeric TRPV4 and TRPC1 channels convey calcium receptor-triggered vasorelaxation via NO generation (Greenberg et al., 2017). In HUVECs, agonist-driven stimulation of the calcium-sensing receptor (CaSR) results in a TRPC1-driven elevation of intracellular Ca^2+^ ion levels and encourages the generation of NO. It has been postulated that engagement of TRPC1 with CaSR and TRPC1-mediated store-operated Ca^2+^ entry (SOCE) mechanisms have been hypothesized to promote an inward flow of Ca^2+^ ions (Qu et al., 2017). TRPC1 co-localizes with TRPV4 in mesenteric artery endothelial cells. This kind of heteromeric channel is triggered through CaSR and enhances NO formation and vasorelaxation (Greenberg et al., 2017; Greenberg et al., 2019).

#### 3.1.3 TRPC6

TRPC6 appears to be a mechanosensitive TRP channel that can be specifically and directly activated via diacylglycerol (Hofmann et al., 1999; Kress et al., 2008). TRPC6 functions to modulate endothelial permeability in reaction to proinflammatory cytokines and inflammatory mediators (Leung et al., 2006; Singh et al., 2007). In pulmonary artery ECs, TRPC6 silencing reduced TRPC6 agonist-driven rise in intracellular Ca^2+^ ion levels, vascular perfusion, and edema production (Samapati et al., 2012). Cytochrome P450 (CYP)-based epoxyeicosatrienoic acids (EETs), which are among the other mechanically generated compounds, promote the displacement of TRPC6 into caveolin-1-rich cell plasma membrane areas, with increased caveola generation upon fluid shear stress (Fleming et al., 2007). The direct mechanical activation of TRPC6 has not yet been clearly identified. The synergistic activation of a dual mechanical and muscarinic receptor agonist carbachol-mediated activation mechanism has been postulated (Inoue et al., 2009a).

#### 3.1.4 TRPM7

TRPM7 expression has been detected within HUVECs (Baldoli et al., 2013), wherein it is coupled to the trafficking of magnesium. TRPM7 is, to some extent, unique relative to other TRPs, in which it contains a regulatory kinase domain located near the C-terminus (Ryazanova et al., 2014). The mechanosensitive capability of TRPM7 has been confirmed in pressure-loaded patch-clamp assays (Xiao E. et al., 2015) and in fluid shear stress studies of mesenchymal stromal cells (Liu et al., 2015).

#### 3.1.5 TRPP2

TRPP2, synonymously referred to as polycystin-2 or polycystic kidney disease 2 (PKD2), has been coupled to mechanosensitive functions of primary cilia. Decreased expression of TRPP2 decreases NO formation of mouse endothelial cells (AbouAlaiwi et al., 2009). Elimination of TRPP2 prevents endothelial cells from converting external shear stress into intracellular Ca^2+^ signals and nitric oxide production (Nauli et al., 2008). An interplay of TRPP2 and TRPC1 and a hypothesized involvement in stretch-driven blood–brain barrier endothelial cell damage are also proposed (Patel et al., 2010; Berrout et al., 2012). Moreover, it has been found that, solely, a heteromeric channel assemblage of TRPP2, TRPC1, and TRPV4 is capable of imparting flux-driven cation flows (Earley and Brayden, 2015).

#### 3.1.6 Kir2.1

In an early effort to identify mechanosensors in endothelial cells, a K^+^ channel was revealed that responds to forces (Olesen et al., 1988). Expression of functional Kir2 channels has been identified in VSMCs (Sancho et al., 2022). Kir2 channels are not subject to conventional regulatory oversight, which is why they are frequently regarded as being merely background conductance (Sancho et al., 2022). Two membrane lipids, phosphatidylinositol 4,5-bisphosphate (PIP2) and cholesterol, provide stabilization of Kir2 channels in a favored open or closed configuration and, in conjunction with the cytoskeleton, CAV1 and syntrophin, endow them with hemodynamic sensitivity.

Kir2.1 channels, which are part of the inwardly rectifying potassium Kir channel family that let larger amounts of potassium ions into the cell than out of it, have been linked to neurovascular coupling (NVC). The cerebral circulation consists of a mesh of interlinked surface vessels with a substantial capacity to reroute the blood flow (Blinder et al., 2013). Arterioles enter the brain in an orthogonal fashion, ramify into smaller arterioles, and merge into capillaries, which vastly expand the zone of blood flow. Capillaries, which comprise the tiniest of all blood vessels, are small of approximately 5-μm diameter ducts composed of a monolayer of capillary endothelial cells. It has been observed that inwardly rectifying Kir2.1 channels in capillary endothelial cells are activated by K^+^ liberated by active neurons, causing a spreading hyperpolarization that propagates upstream and enlarges arterioles, referred to as dilation (Longden et al., 2017). This mechanism is a key driver of the NVC mechanism, which conveys an enhancement of the local blood flow toward active neurons, which is referred to as functional hyperemia (Longden et al., 2017; Harraz et al., 2018a; Dabertrand et al., 2021). Since the red blood cells are a little more than capillary diameter, the blood cells get “squeezed” through the capillary in a single stream, thereby exerting a unique mechanical force on the capillary endothelium. It is currently uncertain how the capillaries of the brain perceive the forces connected with blood motion and whether this mechanosensitivity plays a role in NVC. There is, therefore, a need to investigate this in further studies.

In cultured aortic endothelial cells, a Kir channel has been found to be activated by shear stress. It was hypothesized that this channel participates in flow-based hyperpolarization and the regulatory control of vascular tone (Olesen et al., 1988). This research sparked the pursuit of the function of Kir channels in mechanosensation and regulation of blood flow. The Kir2.1 channel found to be expressed in endothelial cells from various vascular types (Zaritsky et al., 2000; Ahn et al., 2017; Longden et al., 2017), is responsive to extracellular K^+^ levels, and its activation is triggered by hyperpolarization (Hibino et al., 2010). The flow activates Kir2.1 in a way that relies on the level of shear stress and oscillation frequency, which results in K^+^ efflux and hyperpolarization. In addition to regulation of Vm, Kir2.1 activity has been implicated in NO generation (Hoger et al., 2002; Lieu et al., 2004; Ahn et al., 2017). It has been hypothesized that Kir2.1 in endothelial cells is critical for flow-triggered phosphorylation and activation of eNOS and Akt and downstream NO production, probably in a Ca^2+^-independent fashion (Ahn et al., 2017). Shear stress enhances Kir2.1 activity, although the channel may not serve as the primary mechanosensor. Cell-attached electrophysiology was utilized to induce shear activation of Kir channels, although the cell-tethered membrane was not necessarily directly subjected to shear stress (Jacobs et al., 1995). It is probable that some upstream mechanosensing mechanisms are implicated in the reaction of Kir2.1 to shear stress. It is speculated that shear stress changes the lipids and fluidity of the plasma membrane, which then, in turn, impacts the activity of Kir2.1. Kir2.1 is, in fact, an essential regulatory target of phosphoinositides such as PIP2 and cholesterol (Romanenko et al., 2004; Harraz et al., 2018a). In arterial endothelial cells, among ion channel mechanosensors, Piezo1 and Kir2.1 channels convey shear stress-initiated NO liberation, although the Piezo1 channel functions in a depolarizing manner via the Ca^2+^/Na^+^ influx and Kir2.1 in a hyperpolarizing manner via the K^+^ efflux (Wang S. et al., 2016; Ahn et al., 2017). In other investigations, flow-driven activation of the TRP polycystin (PKD2) channel has been connected to the activation of eNOS and K^+^ channel-based hyperpolarization (MacKay et al., 2020). These findings and other related findings underscore the versatility of endothelial signal transduction. Endothelial cells use various mechanisms to provide resilient signaling and mechanotransduction paths in reaction to hemodynamic forces.

#### 3.1.7 ENaC

ENaC is a type of Na^+^ channel that is nonvoltage-dependent, composed of three subunits, constitutive active channel, and an amiloride-sensitive channel. It belongs to the ENaC/degenerin family (Kellenberger and Schild, 2002). ENaC has been originally identified in epithelial cells, and it has been suggested to express exclusively on these cells. The epithelial sodium channel ENaC has been characterized mainly in the principal cells of the distal renal nephron, where it is mostly implicated in water and salt balance homeostasis (Garty and Palmer, 1997; Bhalla and Hallows, 2008). Evidently, ENaC is widely expressed in several different tissues, where it performs various tasks (Zhang et al., 2022). In specific, ENaC has been detected in the vascular endothelium, in which it regulates endothelial mechanical characteristics at the nanoscale (Fels et al., 2010; Tarjus et al., 2017). The endothelial ENaC channel seems to be activated through laminar flow in a direct fashion. ENaC, along with several other ion channels, is connected to cytoskeletal elements, and these connections serve for mechanotransduction purposes (Mazzochi et al., 2006; Ilatovskaya et al., 2011; Fels et al., 2012; Warnock et al., 2014). It is assumed that elevated ENaC activity within the endothelial cells and, thus, enhanced sodium inflow cause the cortical actin to become stable in its filamentous form (F-actin), thereby creating a stiffer cell cortex (Oda et al., 2001; Warnock et al., 2014). AFM-based nanoindentation approaches were employed to demonstrate a relationship between shear stress-induced ENaC membrane abundance, polymerization of actin, and the nanomechanical characteristics of the endothelial cortex. In other words, shear stress under laminar flow resulted in a stiffening of the cortex of the cells by 18.9% ± 5.5% (N = 3, n = 37, *p* ≤ 0.01) relative to static control conditions, which can be attributed to an elevated quantity of F-actin in laminar shear stress (Cosgun et al., 2022). It is important to note that the implementation of non-laminar shear stress enhanced cortical stiffness by a further 67.9% ± 8.9% (N = 3, n = 36, *p* ≤ 0.01), which was accompanied by additional non-laminar shear stress-driven actin polymerization (Cosgun et al., 2022). Consequently, these results emphasized the impact of non-laminar shear stress in a robust *in vitro* model. Moreover, an increase in the ENaC channel density and/or activity, referred to as ENaC gain-of-function (GOF), has been demonstrated to cause endothelial cells to stiffen and subsequently lead to vasoconstriction (Jernigan and Drummond, 2005). ENaC GOF, resulting from elevated channel expression, has been seen in hypertension (Mills et al., 2016; Ahmad et al., 2023). The mechanisms through which ENaC GOF impairs endothelial functioning and shear stress-driven vasodilation include decreased generation of NO. NO loss is the outcome of increased Na^+^ influx, which weakens the driving force for L-arginine, which is a NO precursor, incorporation through cationic amino acid transporters. As an alternative, NO depletion can result from the negative regulation of the phosphoinositide 3-kinase (PI3K)/Akt pathway, which is essential for the synthesis of NO (Stojanovic et al., 2006).

Functional blockade of ENaC can cause a switch from F- to G-actin, resulting in a softening of the cell cortex (Fels and Kusche-Vihrog, 2020). On the contrary, the fixation of the actin cytoskeleton through chemical substances cancels this switching effect. In addition, the decrease in the spacing between the cortex and actin filaments is crucial for the thickness and tension of the cortex (Lembo et al., 2023). Thus, ENaC functioning and actin dynamism are tightly coupled in endothelial cells. In addition, ENaC could be triggered due to the flow and elevated hydrostatic pressure, and raised intracellular sodium concentrations cause lower NO formation in endothelial cells (Guo et al., 2015). In agreement with these results, ENaC has been observed to insert into the plasma membrane of HUVECs (cultured as a 2D monolayer) due to acute shear stress application or alterations (Cosgun et al., 2022). Consequently, an enhanced Na^+^ flow into the endothelial cells and the polymerization of the cortical actin take place. ENaC shear force detection relies on the engagement of sugar residues with the endothelial glycocalyx. Extracellular N-glycosylated asparagine moieties of ENaC bind the channel to both the ECM and endothelial glycocalyx, and elimination of these N-glycans decreases shear force-triggered ENaC flows (Leung et al., 2006). Notably, the endothelial glycocalyx is also engaged in the capacity of endothelial ENaC to moderate flow-mediated vasodilation, and removal of ENaC in endothelial cells abrogates shear stress-based relaxation.

Together, these findings reinforce the concept of a close cooperation and mutual relationship between the endothelial glycocalyx, ion channel functionality, and the endothelial cytoskeleton as linked mechanosensors. Finally, ENaC has a dual function, such as being beneficial or detrimental for the vascular function (Zhang et al., 2022). In summary, normal endothelial ENaC activity promotes shear stress-mediated vasodilation, whereas increased ENaC activity is associated with dysfunction of the endothelium. The differential effect of ENaC on vascular functioning can be attributed to the heterogeneity of the vascular sites and whether ENaC activity is increased in physiological or pathological manners (Zhang et al., 2022).

#### 3.1.8 TREK-1

Two-pore K^+^ channel (K2P channel) superfamily members have been hypothesized to act as endothelial mechanosensors. The K2P channels are widely found to be expressed in various vascular patches, where they control the vascular function (Gurney and Manoury, 2009). TRAAK, TREK-1, and TREK-2 belong to the K2P channel family, and the TREK-1 channel has been thoroughly characterized in endothelial cells (Lesage et al., 2000; Brohawn et al., 2014). TREK-1 exhibits a direct responsiveness to positive and negative pressure within lipid bilayers, and the mechanosensitivity of the channel is maintained in various electrophysiological settings (Patel et al., 1998; Brohawn et al., 2014). TREK-1 is directly controlled through membrane tension; distortion of the plasma membrane removes the lipid blockade of ion conductance and consequently increases the activity of the channel, which results in hyperpolarizing flows (Honoré et al., 2006; Brohawn et al., 2014; Brohawn, 2015; Schmidpeter et al., 2023). TREK-1 not only modifies the V_m_ value of endothelial cells but is also involved in endothelium-driven, NO-facilitated vasodilation (Garry et al., 2007). The TREK-1 channel is not only solely mechanosensitive but also sensitive to osmotic pressure and becomes activated through ischemia-associated swelling. The cerebrovascular TREK-1 channel, therefore, plays a preservative role in the event of ischemia as it hyperpolarizes the endothelial cells and dilates the vessels (Patel et al., 1998; Maingret et al., 1999; Rao et al., 1999). It is hypothesized that hyperpolarization conveyed by TREK-1 may act in synergy with some other hyperpolarizing channels or mechanosensors, such as Kir2.1, or antagonize depolarizing channels, such as Piezo1 or TRPV4. The TREK-1 channel is regulated through GPCRs, with several of them being mechanosensors (Maingret et al., 1999; Lesage et al., 2000; Chemin et al., 2005; Mathie, 2007; Brohawn et al., 2014; Brohawn, 2015). PIP2 increases the activity of TREK-1, and cyclic adenosine monophosphate/protein kinase A (cAMP/PKA) acts to repress the channel. Consequently, TREK-1 activity is inhibited through Gs-PKA (enhanced inhibitory PKA) and Gq-PKA (reduced stimulatory PIP2) but is not blocked through Gi-PKA (reduced PKA) (Chemin et al., 2005; Mathie, 2007). Overall, the function of endothelial TREK-1 in mechanosensing is probably influenced through the other mechanosensitive GPCRs in place.

#### 3.1.9 Future perspectives of ion channel actin as mechanosensors

Many endothelial mechanosensitive ion channels have been examined, and the emerging lines of evidence that Piezo1 is a genuine mechanosensor are very strong. Piezo1 appears to be expressed in most vascular grafts investigated so far, but the functions of endothelial Piezo1 are still not sufficiently characterized, which has been due to the recent detection of Piezo1 and the general relatively low number of specific/selective blockers. There exist inhibitors against the Piezo-ligand Yoda1, such as salvianolic acid B (Pan et al., 2022), and the Yoda1 derivative Dooku1 (Evans et al., 2018; Hatem et al., 2023). Recently, there have also been inhibitors that target Piezo1, comprising the toxin GsMTx4 from tarantulas; specific fatty acids; amyloid beta (Aβ) peptides; metal-containing compounds, such as ruthenium red and gadolinium; the Yoda1 derivative Dooku1; and natural compounds, such as tubeimoside I, salvianolic acid B, jatrorrhizine, and escin (Tapia and Velasco, 1997; Peng et al., 2016; Evans et al., 2018; Thien et al., 2024). The results showed that Piezo1 misexpression can be linked to a variety of chronic illnesses, which range from hypertension to cancer and hemolytic anemia. As a result, the blockade of Piezo1 and the resulting calcium inward current may have favorable implications for several pathological processes as several *in vitro* and *in vivo* investigations have been revealed (Thien et al., 2024). The development of Piezo1 inhibitors is still in its infancy, although, and there are still plenty of options and many challenges to be exploited.

As mentioned above, several mechanosensors cooperate, and Piezo1 may be a crucial element in these mechanisms. It is assumed that progress will be made in the near future in gaining an improved insight into mechanosensors, in general, and Piezo1 as the prevailing channel mechanosensor in endothelial cells. Remarkably, the expression of these ion channel species differs according to the anatomical position of the endothelial cells in the vascular tree. Arterial/arteriolar endothelial cells exhibit IK and SK channels, which are crucial in converting increases in intracellular Ca^2+^ levels into hyperpolarizing/dilator cues, whereas in the capillary endothelium, this ion channel family is lacking (Jackson, 2022). Notably, endothelial cells that do not express voltage-gated channels can be electrically coupled to VSMCs and can, therefore, directly control the basal tone of the vessels (Ebong et al., 2014). Finally, the characteristics of the mechanosensors must be defined to be able to better work out the similarities and differences. In addition, these hallmarks could be used to focus on the essential mechanisms of these receptors in the regulation of vascular functions to be able to intervene in these mechanisms with inhibitors or activators.

### 3.2 G-protein-coupled receptors

Selected G-protein-coupled receptors are briefly presented as mechanosensory units. Canonical activation of a GPCR is induced when an extracellular ligand engages the receptor, thereby initiating second messenger cascades that rely on Ca^2+^ signal transduction, cyclic nucleotides, or β-arrestin. Nevertheless, some GPCRs transduce mechanochemical signaling, regardless of ligand tethering (Takada et al., 1997; Chachisvilis et al., 2006). The mechanisms behind the mechanosensitivity of GPCRs are not yet completely elucidated but probably comprise alterations in membrane fluidity that permit GPCRs to acquire an active configuration even in the complete lack of a receptor agonist (Gudi et al., 1998; Yamamoto and Ando, 2013; 2015). In addition, helix 8, which is a conserved helical motif situated adjacent to transmembrane segment 7, has been demonstrated to confer mechanosensitivity to GPCRs. Structural and functional investigations have revealed that mechanical pacing extends helix 8 and thus stimulates the activation of GPCR (Erdogmus et al., 2019). Multiple mechanosensory mechanisms intertwine. Activation of an endothelial cell mechanosensor is frequently accompanied by metabolic alterations. Shear stress triggers Gq-PCRs and subsequently enhances the production of DAG and IP3 and the degradation of PIP2. Alterations in the intracellular metabolite content have considerable effects on mechanosensitive ion channels. DAG increases the activity of TRPV4 in arterial endothelial cells. Depletion of PIP2 increases TRPV4 and TREK-1 activity and represses the Kir2.1 activity. The activation of Piezo1 can increase AA synthesis, and then, AA triggers TRPV4 and TREK-1 and subsequent reactions (Patel et al., 1998; Chemin et al., 2005; Sonkusare et al., 2014; Harraz et al., 2018b; Harraz et al., 2018b; Swain and Liddle, 2021). The overlaps among various metabolic routes are hence key components of mechanosensory mechanisms.

#### 3.2.1 GPR68

G protein-coupled receptor 68 (GPR68) has been shown to be an endothelial Gq/11-coupled receptor that acts to promote flow-induced vasodilation by increasing NO levels. Deletion of GPR68 compromised flow-based dilation of mesenteric arteries and had no impact on the regulation of blood pressure (Xu et al., 2018). Disruption of the structural integrity of the helix 8 (H8) domain of GPR68 by amino acid replacement limits mechanosensitivity, suggesting that H8 is an essential structural motif that provides mechanosensitivity to GPCRs (Erdogmus et al., 2019). Inconsistent findings employing cultured cells, nevertheless, revealed that flow-driven GPR68 activation is sustained, regardless of H8 removal or the pharmacological blockade of G protein signal transduction (Ozkan et al., 2021). The latter is in accordance with the piece of evidence that shear stress triggers the activation of Gq/11, regardless of the activation of GPCRs in cultivated endothelial cells (Dela Paz et al., 2017).

#### 3.2.2 Bradykinin 2 receptor

Another type of endothelial mechanosensors is the Gq/11-coupled bradykinin-2 (B2) receptors (Chachisvilis et al., 2006). Shear stress, hypo-osmotic excitation, or a modification of plasma membrane fluidity stimulates the B2 receptor, resulting in an elevation of intracellular Ca^2+^ ion levels. Ca^2+^ signal transduction facilitated through the endothelial B2 receptor causes vasodilation through enhancing NO generation or stimulating Ca^2+^-driven hyperpolarization (Liao and Homcy, 1993; Chachisvilis et al., 2006; Hu et al., 2022). In addition, the endothelial B2 receptor increases the generation of AA, which can elevate the Ca^2+^ flux through TRPV4 (Watanabe et al., 2003), indicating a possible link between the B2 receptor and other mechanosensors.

#### 3.2.3 Endothelial sphingosine-1-phosphate receptor 1 and histamine H1 receptor

Shear stress has been found to induce activation of sphingosine-1-phosphate receptor 1 (S1PR1) and the histamine H1 receptor (H1R) in endothelial cells. Activation of S1PR1 triggers NO synthesis, which causes vasodilation. S1PR1 is likewise critical for vessel development and homeostasis of blood pressure (Jung et al., 2012; Cantalupo et al., 2017). Overall, the results indicate that S1PR1 acts as a pivotal element in an endothelial mechanosensory signal transduction route, behaving more as a mechanotransducer than as a sensor. H1R contains helix 8 and has thus been described as an endothelial mechanosensitive GPCR that can promote NO signal transduction and vasodilation (Erdogmus et al., 2019).

#### 3.2.4 Perspectives for the mechanosensitive GPCRs

Endothelial GPCRs that act as putative mechano-GPCRs comprise GPR68, B2 receptor, S1PR1, and H1R. Mechanosensing evidence indicates that GPR68 may fulfill the most important function. The expression at the messenger RNA (mRNA) stage, nevertheless, is most prominent for S1PR1 and tends to be minor for other culprits (Vanlandewijck et al., 2018). Moreover, some putative mechanosensitive Gq/11-coupled receptors are lacking indications of a direct activation through mechanical forces when exposed to heterologous expression networks, confirming the concept that GPCRs are implicated in mechanosensation in an indirect manner (Marullo et al., 2020). The downstream actions of GPCR signaling intersect with and modify the activity of other mechanosensitive ion channels; the complement of the endothelial cell receptors may hence orchestrate and control the mechanosensitive ion channel activity (Harraz et al., 2018b). This indicates that the mechanosensors work together, are functionally linked, and thus may be able to cancel each other out.

### 3.3 Cell matrix receptors such as integrins

Another focus of the review article is placed on apical mechanotransduction and on cell–matrix mechanotransduction. Cell–matrix receptors like integrins act as the major mechanosensors on the basal face of the endothelial cell, establish a direct connection from the actin cytoskeleton to the ECM (Geiger et al., 2009; Eyckmans et al., 2011), and are implicated in the reaction toward substrate-related and flow-related stimuli (Tzima et al., 2005).

Integrins constitute heterodimeric adhesion receptors consisting of α- and β-subunits that are capable of tethering extracellular matrix compounds, among other ligands, that are essential for basement membrane attachment (Hynes et al., 2002). Integrins can create 24 different, specific integrin heterodimers. Various integrin heterodimers couple to various ligands, such as fibronectin receptors, collagen receptors, laminin receptors, and leukocyte-specific receptors (Hynes, 2002). Expression of integrins is specific to cells, and the various α/β-subunit pairs generated determine the types of extrinsic ligands that cells can engage, impacting their differentiation and performance. Integrins are connected to the actin cytoskeleton via focal adhesion proteins, such as talin, vinculin, kindlin, and paxillin, which also serve as signal transduction centers that promote intracellular signal transmission (Sun et al., 2019; Kadry and Calderwood, 2020). Integrin signaling is unique because it is bidirectional as it can be triggered by the engagement of external ligands (outside-in signaling) or by the engagement of cytosolic scaffold proteins (inside-out signaling) (Hynes, 2002; Ye et al., 2012; Humphries et al., 2019; Kadry and Calderwood, 2020). Most of the knowledge about integrins and their functioning stems from the examination of cell–cell interactions among leukocytes (Chigaev and Sklar, 2012; Martín-Cófreces et al., 2018) and cell/ECM adhesion at the basolateral cell surface (Schwartz, 2010; Geiger and Yamada, 2011).

The structure and conformation of integrins are crucial to each part of its operation, ranging from adhesion to signal transduction. The α-subunit comprises a head domain and two leg domains (Luo et al., 2007; Campbell and Humphries, 2011). Half of the α-subunits possess an I domain incorporated into the head domain, which enables them to bind divalent metal ions that can function as an activation switch (Luo et al., 2007; Campbell and Humphries, 2011). Similar to the α-subunit, the β-subunit contains leg domains in the upper and lower parts of the molecule, as well as a head domain with a cation-tethering I-like domain (Luo et al., 2007; Campbell and Humphries, 2011). This structure allows both α- and β-subunits to take on conformations with bent and stretched head groups (Luo et al., 2007; Campbell and Humphries, 2011). In inactive conditions, integrins exist in a bent conformation (Takagi and Springer, 2002; Luo et al., 2007; Ye et al., 2012). The conformational rearrangement that enables the head group to switch to an outstretched conformation coincides with activation (Takagi and Springer, 2002; Ye et al., 2012). Activation is a prerequisite for the engagement of integrins with ligands and for the transduction of intracellular cues. Following activation, integrins exhibit a higher affinity for ligand engagement (Luo et al., 2007; Ye et al., 2012). Although the elongated head group is a characteristic of activated integrins, they can also assume intermediate conformations, such as an elongated closed conformation in which the head group is turned outward but still exhibits a low affinity for the linking ligand (Ye et al., 2012; Su et al., 2016; Li and Springer, 2017).

The resting endothelium expresses a minimum of seven classes of integrins, where β1-integrin, being one of the most well-characterized in the endothelium, confers the specificity for fibronectin (α5β1), laminin (α3β1 and α6β1), and collagen (α1β1 and α2β1) (Stupack and Cheresh, 2002). Simultaneous endothelial-specific α5 and αv deletion results in heart vascular defects and in embryonic lethality up to E14.5 (Van Der Flier et al., 2010). Endothelial cell-specific deletion of β1-integrin throughout development led to embryonic lethality ranging from E9.5-E.10 .5, which has been found to be marked through defective vascular patterning and vascular abnormalities (Lei et al., 2008). Inducible genetic knockdown of β1-integrin in endothelial cells or postnatal pharmacological inhibition of β1-integrin compromised lumen generation and caused apical–basal polarity defects in endothelial cells throughout new vessel outgrowth (Zovein et al., 2010). Moreover, endothelial cell-specific genetic deletion of β1-integrin in mice provides strong evidence for a regulatory mechanism of β1-integrin expression in the stabilization of VE-cadherin at cell–cell junctions (Yamamoto et al., 2015). Overall, these findings imply that the expression of β1-integrin in endothelial cells is decisive for proper vascular development and the stability of blood vessels.

Even though the abovementioned examples only deal with monovalent molecular interactions, there are multivalent interactions in several receptor–ligand systems. P-selectins and E-selectins, for example, can form dimeric links with the dimeric P-selectin-GP ligand 1 (Ramachandran et al., 2001; Zhang et al., 2013). Fibrinogen and VWF are each able to attach multiple replicas of their corresponding receptors to the identical or distinct platelets (Rooney et al., 1998; Zhou Y.-F. et al., 2012). The assembly and disassembly of these intricate linkages involve multi-step kinetic mechanisms that include intermediary events with additional speed variables. In addition, the force loading is probably non-uniformly spread over multiple bonds, which complicates the force sensitivity to bond lifetime (Friddle et al., 2012). Nevertheless, it is probable that a multimeric ligand has a higher probability to reach the cell receptors for elevated mechanopresentation. The total lifetime of attachment is likely also extended by the fact that disconnected elements of the receptor–ligand cluster are maintained in close distance by other members and stay assigned so that they can rapidly reconnect, leading to enhanced mechanoreception. Although cross-linking of receptors by multimeric ligands promotes cell signaling in multiple settings, multivalence may eventually guarantee enhanced mechanosensing. Although the formation of clusters results in a higher local accumulation of signaling molecules to amplify the signaling response, the generation of multiple tethers requires a reduction in the force distributed across the individual linkages, which can attenuate the signaling effect of the individual receptors. When the mechanosensors switch to a threshold mechanism that demands a specific force amplitude at individual receptors, then excessive force scattering may disable the transmission of signals. Moreover, although several proteins lack a dimeric/multimeric architecture, they include binary or plural binding sites that can independently or cooperatively engage. For instance, fibronectin incorporates a synergistic site in its III9 domain that enhances the engagement of the RGD sequence of the III10 domain with integrin α5β1 (Friedland et al., 2009; Kong et al., 2009). Thy-1 is able to concomitantly engage the integrin α5β1 and syndecan-4 in a force-triggered cooperative fashion (Fiore et al., 2014). Consequently, mechanoreception is amplified in these two events.

β1-integrins can sense the direction of the vascular flow (Xanthis et al., 2019; Su et al., 2024). Thereby, the activation of β1-integrins seems to be critical and mandatory for endothelial cell-alignment at locations of the unidirectional flow (Vion et al., 2021). In addition, reduced tension across intercellular VE-cadherin junctions and, thus, impaired lateral forces between adjacent endothelial cells have been demonstrated to prevent endothelial cells from collectively polarizing, such as in processes of vascular wounding, following physical damage. At sites of bidirectional flux, β1-integrins fail to be activated, which is a pivotal mechanism for unraveling the mechanics of blood flow to foster vascular homeostasis. A unidirectional shear stress leads to orientation of the endothelial cells with a simultaneous rest phase, while bidirectional and other irregular shear stress patterns fail to encourage orientation (Sorescu et al., 2004; Wang et al., 2006; Wang et al., 2013; Wu et al., 2011; Feaver et al., 2013; Ajani et al., 2015). The shear stress-mediated activation of α5β1 integrins induces Ca^2+^-dependent signal transduction processes (Matthews et al., 2006; Loufrani et al., 2008; Thodeti et al., 2009; Buschmann et al., 2010; Yang et al., 2011, p. 190), which then guides the migration and invasion of endothelial cells (Urbich et al., 2002) and endothelial inflammatory events (Bhullar et al., 1998; Dekker et al., 2005; Luu et al., 2013; Chen et al., 2015; Sun X. et al., 2016; Yun et al., 2016; Budatha et al., 2018). A model for the integrin involvement in shear stress signal transduction assumes that the tension produced at the apical surface is transferred through the cytoskeleton to the integrins positioned at the basal surface, causing structural alterations that raise their binding capabilities for ECM ligands (Bhullar et al., 1998; Tzima et al., 2001; Orr et al., 2005; Orr et al., 2006; Puklin-Faucher and Sheetz, 2009). Experiments with a chimeric form of the α5 integrin, replacing the cytoplasmic region with that of the α2 integrin, have revealed that the flow fuels ECM-driven signal transduction through the basally situated α5 integrin to facilitate the inflammatory activation of endothelial cells (Yun et al., 2016; Budatha et al., 2018). Nevertheless, further investigations revealed that integrins expressed on the apical face of endothelial cells can also react to mechanical force (Conforti et al., 1992; Matthews et al., 2006), which has been refined thereafter (Xanthis et al., 2019). The following indications lead to the deduction that β1-integrins are force direction-sensitive elements. First, β1-integrin transforms from a deflected inactive shape to an elongated active mode as a reaction to a unidirectional shear force, as contrasted to a bidirectional shear force. Second, steered molecular dynamics (SMD) simulations demonstrated that a force exerted along parallel to the membrane can produce structural redistributions, resulting in elongation of the β1-integrin. Third, unidirectional shear force initiates Ca^2+^ signal transduction through a β1-integrin-driven pathway, while the response to bidirectional force is completely unrelated to β1-integrins. Fourth, deletion of β1-integrin inhibited the orientation of cultured endothelial cells subjected to unidirectional shear stress, whereas it failed to change the shape of cells undergoing bidirectional shear stress. Fifth, β1-integrins underwent selective activation at locations of unidirectional shear stress within the mouse aorta (Xanthis et al., 2019), and sixth, removal of β1-integrin from endothelial cells diminished the orientation of endothelial cells at locations of unidirectional shear stress across the aorta of mice but made no changes to their shape at locations of the disturbed flow (Xanthis et al., 2019).

#### 3.3.1 Basal or apical position of integrins and their reaction to flow

A question arises whether the location of integrins, such as at the basal or apical site of endothelial cells, plays a role in the sensing and reaction to flow-induced shear stress. Another important question that arises is whether apical integrins can transduce physical signals without being tethered to an extracellular substrate. In contrast to basal integrins, which organize focal adhesions or cell–cell interactions, apical integrins are usually distributed over the entire plasma membrane, complicating their assignment to specific structures or molecular complexes. The treatment of human umbilical vein endothelial cells (HUVECs) with a type I collagen hydrogel on the apical surface causes the rearrangement of α2β1 integrins on the apical cell surface (Turner et al., 2020). Activation of α2β1 integrins has also been shown to be an essential step in fast tube assembly and angiogenesis (Turner et al., 2020). The administration of collagen coating on the apical surface of the cell has no influence on the size of the apical β1-integrin pools (Zuk and Matlin, 1996). This indicates that the apical integrins themselves do not influence polarity but that the interplay between the apical integrins and external ECM proteins favors a reorientation of the apical/basolateral polarity axis (Ojakian and Schwimmer, 1994; Zuk and Matlin, 1996).

Interrupting integrin–ligand adhesion with blocking antibodies against α5 or β1 reduces cell adhesion and enhances cell migration (Akiyama et al., 1989). In accordance with this model, integrins are present on the upper surface of migrating skin fibroblasts, as determined by FRAP, whereas in stationary cells, they are clustered into fibrillar streaks (Duband et al., 1988). In line with this finding, cells exhibit high migratory activity when the apical pools of β1-integrins on F98 cells adopt the closed conformation (Holz et al., 2000). Conversely, exposure of cells to ligands that both cluster and activate apical β1-subunits decreases focal adhesion and cellular stretch, thereby impeding migration (Mang et al., 2020). Consequently, these investigations imply that the interaction between apical integrins and specific ligands acts as a molecular on–off switch that regulates cell motility.

**FIGURE 4:**
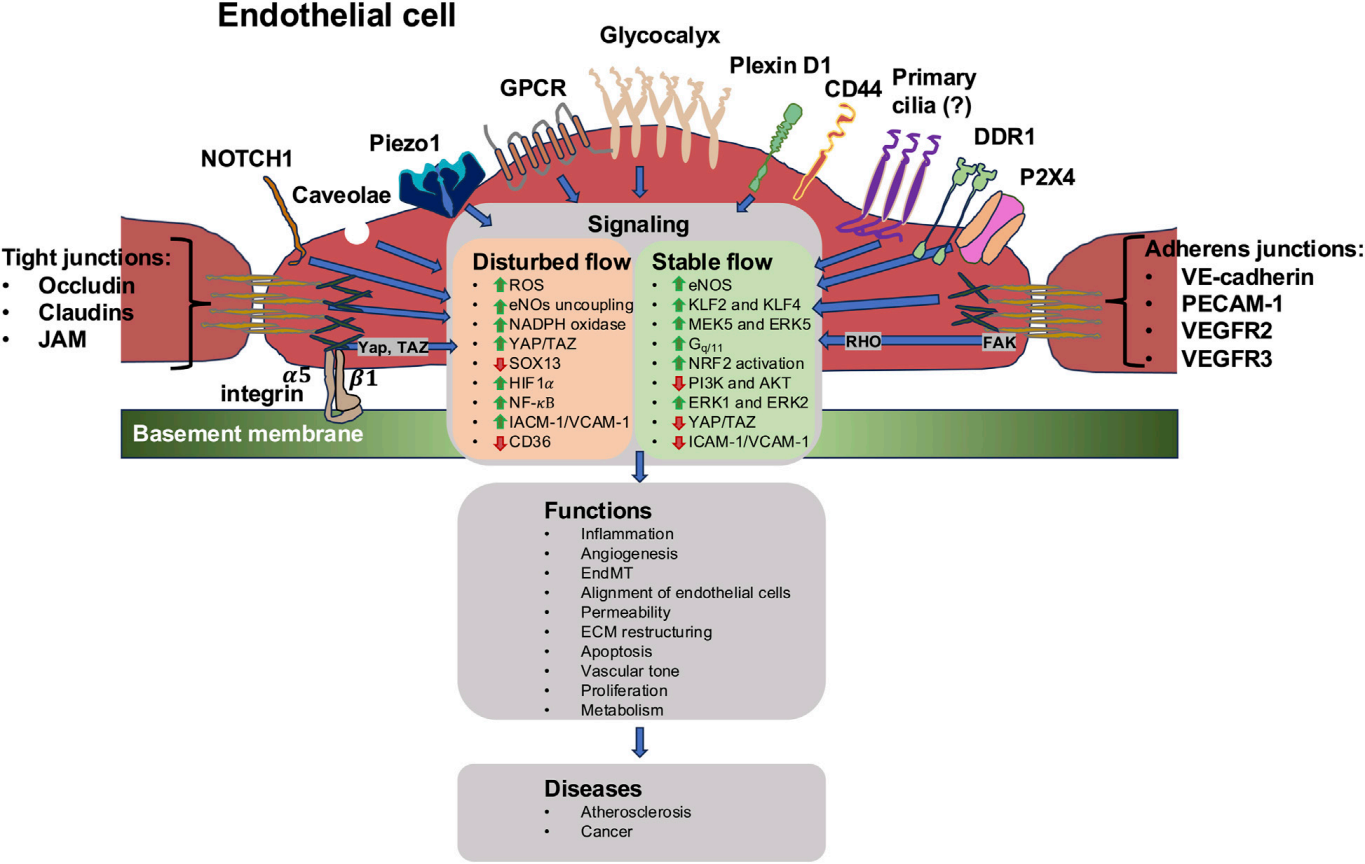
The apical surface of the endothelial cell possesses mechanosensory proteins, such as Plexin D1, NOTCH, Piezo1, P2X4, DDR1, CD44, and G protein-coupled receptors, such as GPR68 and mechanosensitive structures like caveolar, glycocalyx, and primary cilia. Cell–cell junctions, such as adherens junctions, compromise VE-cadherin, PECAM-1, vascular endothelial growth factor 2 (VEGFR2), and VEGFR3. At the basal site of the endothelium is the contact to the basement membrane via integrins, such as α5β1 , which also act as mechanosensors. All these mechanosensors contribute to mechanotransduction processes, including the PI3K-Akt pathway, YAP-TAZ pathway, ERK1-ERK2 pathway, and Rho signal transduction pathway. Several mechanotransduction routes lead to the activation of transcription factors, such as Krüppel-like factor (KLF2) and KLF4, hypoxia-inducible factor 1 α , and nuclear factor- κ B (NF- κ B); endothelial-to-mesenchymal transition (EndMT); junctional adhesion molecule (JAM); focal adhesion kinase (FAK); endothelial nitric oxide synthetase (eNOS); reactive oxygen species (ROS); transcription factor SOX13; and nuclear factor erythroid 2-related factor 2 (NRF2). The mechanosensors affect multiple endothelial vascular functions and can lead to diseases upon the perturbed flow, such as cancer and atherosclerosis.

At first glance, it seems reasonable that the functionality of α5β1-heterodimers differs according to their position on the basal or apical site. For example, basally positioned integrins are activated upon perturbed flow (Figure 4) (Sun Z. et al., 2016), while apically situated integrins are exclusively activated upon unidirectional flow. The mechanisms by which endothelial cells incorporate these diverse types of proximal cues from apical and basal compartments of β1-integrins deserve closer examination. Flow dynamics dictate that blood flow near the vessel wall approaches zero speed (no slippage), and therefore, it is uncertain how shear stress acts to activate proteins at the apical surface. Nevertheless, endothelial cells harbor structures that protrude into the vessel lumen and could be relevant for mechanosensing; among them are the primary cilium and the glycocalyx, which is a coating of glycolipids, glycoproteins, and proteoglycans present on the apical face of endothelial cells. Despite the glycocalyx frequently being lacking in cultured endothelial cells, like HUVECs (Chappell et al., 2009), the glycocalyx has been detected on the arterial endothelium where it may impart shear forces toward the apical face of endothelial cells (Bartosch et al., 2017). It has been established that β1-integrins became activated at the apical face of the aortic endothelium of mice, and it seems to be intriguing to conduct future experiments on the regulatory impact of the glycosylation of β1-integrins (Xu Z. et al., 2019) and the glycocalyx of this kind of process.

#### 3.3.2 Force sensors and anisotropic distribution of forces

Typically, integrins are located on basolateral and lateral cell surfaces, where they promote tethering to the ECM and intercellular adhesion via cytosolic engagement proteins that drive the arrangement of actin microfilaments. Thereby, force transmission plays a pivotal role. So far, it appears that the molecular complexes utilized to drive cell signal transduction via apical integrins are the same as those utilized by integrins in the general setting. Apical-localized integrins, nevertheless, diverge from ECM-localized integrins with respect to their ability to bind ECM proteins, a feature that may affect their physiological role. Apical integrins are not frequently organized into immobile focal adhesions and may, thus, be more quickly rearranged in reaction to different stimuli, as compared to ECM-bound integrins. Moreover, the apical integrin function is most likely affected by the local environment of the apical plasma membrane, which is clearly distinct from the basolateral plasma membrane. They also have entry to apical ligands, comprising ECM elements that are apically secreted, particularly fibronectin.

The accumulation of apical integrins, especially in activated integrins, seems to be a critical step in triggering cellular responses ranging from cell migration to enhanced permeability (Ojakian and Schwimmer, 1994; Zuk and Matlin, 1996; Walsh et al., 2015; Stewart et al., 2017; Badaoui et al., 2020; Mang et al., 2020; Turner et al., 2020). Apical integrin clusters can also promote endocytosis, which may be physiological in the event of integrin turnover in the course of cell migration. How apical integrin clustering is connected to integrin conformation and activation is poorly understood. A number of investigations have shown that integrin activation, typically by ligand binding (Ojakian and Schwimmer, 1994; Zuk and Matlin, 1996; Badaoui et al., 2020; Mang et al., 2020; Turner et al., 2020) but not strictly in clustering (Xu et al., 2016), was needed to trigger a reaction in the cells. β1-integrins tend to be linked to focal adhesion, which comprise nanoclusters with specific populations of both active and inactive integrins, indicating two independent reservoirs according to their activation state (Spiess et al., 2018). Considering the transition from freely mobile integrins to clustered, immobile integrins that occur when cells stabilize (Duband et al., 1988), it is probable that there is also a connection between clustering and the integrin inactivation state at the apical surface.

Similar to their basolaterally localized equivalents, apically localized integrins engage with the actin cytoskeleton (Tafazoli et al., 2000; Li et al., 2009; Yang and Rizzo, 2013; Uehara and Uehara, 2014; Walsh et al., 2015; Badaoui et al., 2020; Mang et al., 2020). The apical integrins may access unique actin pools that differ from actin engaged by integrins involved in focal adhesion. For example, a membrane-proximal pool of F-actin has been characterized, and if apical integrins mainly recruit to this pool of actin, this could account for the participation of apical integrins in processes like apical signal transduction, mechanosensing, and control of barrier function (Bisaria et al., 2020). Identification of specific actin patches preferentially controlled by apical integrins will contribute to the refinement of hypotheses associating them with the organization of other actin-binding proteins, like ZO-1, involved in tight junctions (Belardi et al., 2020).

To elucidate how apical integrins engage the actin cytoskeleton, it is critical to identify how scaffolding proteins can be attracted by stimulation of apical integrins. At present, there is little data on how apical integrin stimulation influences association with scaffold proteins, like kindlin, vinculin, or focal adhesion kinase, even though some indirect evidence indicates a rearrangement of talin in reaction to stimulation with apically presented nanostructured surfaces (Huang et al., 2020). In the actin cytoskeleton, the apical integrins are also associated with alterations in lipid organization of the plasma membrane, increasing ceramide accumulation and decreasing sphingosine (Grassmé et al., 2017). Similarly, data connecting caveolin, eNOS, and connexin hemichannels to apical integrin-facilitated mechanosensing (Batra et al., 2012; Yang and Rizzo, 2013; Liu J. et al., 2020) broaden the range of proteins that associate with integrin past actin and canonical integrin scaffolding proteins. A technique that uses antibodies to coat polystyrene beads mimicking the polyvalency of collagen fibrils causes the formation of tubes similar to the effect of collagen (Turner et al., 2020). This implies that, at a minimum, apical integrin stimulation on its own is capable of producing the same results.

Nevertheless, the apical β1-integrins can perceive force even without ligand engagement. Although apical β1-integrins are unattached to the ECM scaffold, it can be hypothesized that in cells subjected to flows, they function as “sea anchors,” facilitating the transmission of force toward the cell. Therefore, it was hypothesized that unidirectional shear stress triggers tension in β1-integrins that results in downstream signal transduction cascades, while bidirectional shear stress is inadequate as it reverses the flow direction prior to tension buildup (Xanthis et al., 2019). This concept is in agreement with similar findings, which have revealed that mechanical forces are capable of activating β1-integrins, regardless of whether the ligand is tethered or not (Ferraris et al., 2010; Petridou and Skourides, 2016). The direction-specific β1-integrin signal transduction event is anisotropic as it is increased in cells oriented in the force direction, whereas it is diminished in cells subjected to the flow in the reverse or tangential direction. The underlying principle of anisotropy is not determined, but it can be assumed that mechanisms are implicated that restrict rotational diffusion and keep the β1-integrin aligned. The mechanism is likely to entail a flow-driven alteration of actin dynamism or membranous fluid dynamics (Sun Z. et al., 2016), both of which are characterized to affect the alignment and activity of integrins. Because β1-integrin advances endothelial cell orientation, it can be conversely inferred that there is a forward loop present from the activation of β1-integrin to the orientation. Feed-forward schemes are essential for maintaining physiological robustness, and thus, the positive interplay between endothelial cell orientation and β1-mechanosensation is postulated to sustain long-term homeostasis at areas of unidirectional flow.

#### 3.3.3 β1-integrin-dependent Ca^2+^ signaling engages with TRPV4 and Piezo1

There is a requirement to clarify whether β1-integrin-driven Ca^2+^ signals engage Piezo1 and TRPV4 as these Ca^2+^-permeable channels are recognized to perceive shear stress (Köhler et al., 2006; Thodeti et al., 2009; Mendoza et al., 2010). Therefore, HUVECs have been modified with specified siRNAs to inhibit Piezo1 or TRPV4 before applying unidirectional force via magnetic tweezers paired with 12G10 (anti-β1-integrin antibody that targets the β1-domain)-coated superparamagnetic beads (Xanthis et al., 2019). Piezo1 or TRPV4 gene silencing markedly decreased the buildup of Ca^2+^ in HUVECs subjected to the unidirectional flow, implying that the two channels are engaged in Ca^2+^ signal transduction. In agreement with this, β1-integrin-driven Ca^2+^ signal transduction is markedly diminished upon EGTA challenge, suggesting a need for extracellular Ca^2+^. In sharp distinction, the reaction to bidirectional flux upon application of EGTA was only marginally and not significantly decreased. Turning off Piezo1 or TRPV4, nonetheless, had no effect on β1-integrin signal transduction in reaction to unidirectional flow, indicating that these channels are not operating upstream of β1-integrin. It can be inferred that a unidirectional force that triggers Ca^2+^ signaling through a mechanism necessitates β1-integrin-driven activation of Piezo1 and TRPV4 linked to extracellular Ca^2+^, while in contrast, the bidirectional force is initiated by a β1-integrin-independent route. As the shear stress-induced integrin αvβ1 primarily tethers with TGF-β, fibronectin, neural cell-adhesion molecule L1, and osteopontin (Takada et al., 2007), it is likely that, thereby, the ligands contribute to the phenotypic switch of endothelial cells to mesenchymal cells. Consistent with these results, both laminar and perturbed flows can trigger activation of the identical early mechanosignaling route comprising Piezo1- and Gq/G11-based signal transduction (Albarrán-Juárez et al., 2018). According to the flow characteristics, however, the endothelial cells interpret these signaling events alternatively as atheroprotective cues, leading to the activation of eNOS or as inflammatory cues, causing the activation of NF-κB. This variable responsiveness of cells to the initial mechanotransduction event relies on α5-integrin activation, which is enabled only by the perturbed flow and not by the persistent laminar flow (Albarrán-Juárez et al., 2018).

#### 3.3.4 Integrins engage via talin with the adherence junction protein VE-cadherin

A key feature of integrins is well known to be affinity control for extracellular ligands, a process referred to as integrin activation or “inside-out integrin signaling.” An essential terminal stage in the activation of integrins is the tethering of the N-terminal head domain of the cytoskeletal protein talin to the cytoplasmic domain of β-integrins (Calderwood et al., 1999; Tadokoro et al., 2003; Wegener et al., 2007; Ye et al., 2011). Although evidence has emerged on a lot of molecular and structural particulars of how talin engagement activates integrins (Wegener et al., 2007) and the biological relevance of talin-driven integrin activation in hematopoietic cells (Nieswandt et al., 2007; Petrich et al., 2007; Stefanini et al., 2014; Yago et al., 2015; Klann et al., 2017), the precondition for talin-driven integrin activation in committed blood vessels has not yet been addressed so far. This issue was addressed by using a mouse model with deleted talin. Embryonic lethality results from endothelial cell-specific deletion of Tln1 in mice because of deficiencies in angiogenesis, leading to widespread vascular hemorrhage and lethality up to E9.5 (Monkley et al., 2011). This finding substantiates a distinct role of talin in angiogenesis during embryonic development. Mice were studied where Tln1 was genetically removed selectively in the endothelium of mature adult mouse blood vessels utilizing an inducible conditional Cre/loxP recombination setup. These results highlight the significance of talin1 in endothelial cells for the stabilization and barrier role of the visceral microvasculature (Pulous et al., 2019). In addition, both *in vivo* and *in vitro* studies support a function for talin in VE-cadherin regulation, and talin-driven activation of β1-integrin is a pivotal hub in this signaling pathway necessary for adherence junction stability and endothelial integrity (Pulous et al., 2019). Consequently, VE-cadherin is not independently regulated of other cell surface receptors.

#### 3.3.5 Discoidin domain receptor 1 (DDR1)

The tyrosine kinase discoidin domain receptor 1 (DDR1) is critical for normal embryonic development and organogenesis and is also associated with the advancement of several diseases, such as different types of cancer, atherosclerosis, and fibrotic illnesses (Borza and Pozzi, 2014). DDR1 exhibits mechanoresponsivity to the stiffness of the ECM scaffold (Coelho et al., 2017; Ngai et al., 2022; Wang et al., 2022c; Liu et al., 2023a). DDR1 consists of an N-terminal extracellular discoidin (DS) domain and a DS-like domain, a juxtamembrane (JM) extracellular region, a transmembrane helix, a large cytosolic JM region, and a tyrosine kinase domain at its C-terminal end (Leitinger, 2014). The binding of collagen to the DS domain of dimerized DDR1 leads to activation of the receptor by oligomerization and consequent autophosphorylation of cytoplasmic tyrosine residues (Chen and Lin, 2019). A disturbed flow enhances, while a laminar flow attenuates endothelial YAP activity with respect to dephosphorylation and nuclear translocation (Wang K.-C. et al., 2016; Wang L. et al., 2016). The activation of the YAP signal transduction path fosters endothelial inflammation and impaired vascular function, leading to atherogenesis (Wang L. et al., 2016; Wang K.-C et al., 2016). A key outstanding question in YAP signal transduction pertains to the way in which external mechanical stimuli are converted and transmitted to the YAP upstream regulator. Several mechanosensory molecules and complexes, including integrins and focal adhesion proteins, have been linked together (Elosegui-Artola et al., 2017; Nardone et al., 2017). DDR1 was shown to transduce ECM stiffness-triggered YAP activation in vascular smooth muscle cells (Ngai et al., 2022; Liu et al., 2023a). It is uncertain, nevertheless, as to whether endothelial DDR1 senses the flow to orchestrate YAP. DDR1 has been seen to act as a direct mechanosensor in endothelial cells, which governs the cellular responsiveness toward shear flow and consequently the site-specific distribution of atherosclerosis (Liu et al., 2023b). The function and mechanism of its DS-like domain in force-driven oligomerization of DDR1 have been elucidated, and it has been found that force-activated DDR1 assembles liquid-like biomolecular condensates with the 14-3-3ε protein (YWHAE) to block phosphorylation and cytoplasmic retention of YAP. Subsequently, DDR1 tyrosine kinase was identified as a direct mechanosensor and proved to be indispensable for transmitting the force exerted by shearing to endothelial reactions. The flow-driven activation of endothelial DDR1 seems to be atherogenic. The shear force probably results in conformational rearrangement of the DDR1 ectodomain, deploying its DS-like domain to uncover the hidden cysteine-287, whose uncovering promotes force-based receptor oligomerization and phase separation. Upon shear stress, DDR1 generates liquid-like biomolecular condensates and co-condenses together with YWHAE, resulting in YAP nuclear translocation. These results demonstrate a hitherto underappreciated role of DDR1 in directly sensing the flow, suggest a conceptual scheme for how endothelial DDR1 upstream governs YAP signaling, and offer a mechanism through which endothelial DDR1 activation fosters atherosclerosis. Among these mechanosensors, YAP/TAZ has been shown to promote an inflammatory and atherogenic phenotype in endothelial cells of the aortic arch (Wang K.-C. et al., 2016; Wang L. et al., 2016). Laminar flow enhances integrin α5β1–Gα13 coupling and represses YAP through decreased RhoA activity (Wang L. et al., 2016), whereas the disturbed flow enhances active nuclear YAP through the integrin α5β1 signaling path and c-Abl kinase (or ABL1) to boost downstream ICAM1 and VCAM1 expression and atherosclerosis (Li et al., 2019). The YAP/TAZ target CCN1 enhances atherosclerosis through tension-driven engagement of α6β1 integrin to activate downstream NFκB (Hsu et al., 2019). In particular, the transcription factor BACH1 has been characterized as a mechanosensor that moves into the cell nucleus when flow is disturbed, where it functions as a YAP-binding partner (Jia et al., 2022). In cases of the disturbed blood flow, the pro-inflammatory transcription program BACH1-YAP promotes the progression of atherosclerosis. In addition, it has been shown that BACH1 directly activates YAP expression by interacting with the YAP promoter. The mechanosensor JCAD has been pinpointed in genome-wide association analyses of patients with coronary heart disease (Erdmann et al., 2011), and JCAD deficiency attenuated atherosclerosis in Apoe-knockout mice (Xu S. et al., 2019; Douglas et al., 2020). In human endothelial cells, JCAD depletion has also been demonstrated to suppress YAP activity through stabilizing its interaction with the cytoskeletal modulator TRIOBP to prevent its entrance into the cell nucleus (Xu S. et al., 2019). This blocked the expression of its subsequent atherogenic target CCN1 and verified that JCAD encourages endothelial malfunction, inflammation, and atherosclerosis (Xu S. et al., 2019). Despite reduced VCAM1 and ICAM1 levels detected in the aortic arch, consistent with an atheroprotective phenotype, enhanced VCAM1 expression has been observed in the descending aorta of Jcad-knockout mice, indicating that Jcad could also have a site-specific effect in contributing to atherosclerosis (Douglas et al., 2020). Impairment of function experiments showed that DDR1 is important for shear stress-driven alignment of endothelial cells and affects the modulation of mechanoresponsive genes. In addition, direct mechanical force measurements employing magnetic tweezers to exert minimal tensile force on DDR1 expressed on endothelial cell surfaces led to an inflow of calcium. This is critical since small-scale forces, like 4 pN, do not affect the plasma membrane or some other mechanosensors (Monticelli et al., 2016). Using a FRET-based membrane-bound tension biosensor provided evidence that shear force-based DDR1 activation was, at minimum, partially tension-based in terms of DDR1 droplet formation and droplet number enhancement. This mechanism has earlier been proposed for other well-known mechanosensors like the mechanosensitive ion channel Piezo1 (Lewis and Grandl, 2015; Lai et al., 2022) and mechanosensitive GPCRs (Erdogmus et al., 2019).

### 3.4 Junctional proteins act as mechanosensors

There are three major types of cell–cell junctions: adherence junctions, tight junctions, and gap junctions. The adherence junctions have been identified to be highly active in mechanosensation processes, and thus, the focus is placed on them. A brief discussion on the role of tight junctions and gap junction is provided. The adherence junctional proteins PECAM-1, VE-cadherin, and VEGFR2, a tyrosine kinase receptor, have been shown to be abundantly present and required for shear stress signaling within endothelial cells (Yamada et al., 2005). Recently, another junctional protein plexin D1 has been revealed. They are all described briefly in the following.

#### 3.4.1 PECAM-1

The adhesion molecule PECAM-1 is a member of the immunoglobulin superfamily. The extracellular domain of PECAM-1 that facilitates hemophilic engagement with adjacent endothelial cells comprises six Ig-like subunits. The intracellular and extracellular domains of PECAM-1 are linked through a short transmembrane domain. The most crucial characteristics of the intracellular domain include two immunoreceptor tyrosine-based inhibitory motif domains. These domains comprise two tyrosine residues, namely, 663 and 686, which are subject to fast phosphorylation upon the initial application of shear stress. Together with the Ca^2+^ inward current and the activation of the K^+^ channel, the phosphorylation of PECAM-1 is the first recognized reaction to shear stress. It is assumed that the phosphorylation of PECAM-1 occurs regardless of the calcium influx since Ca^2+^ agonists themselves cannot enhance PECAM-1 phosphorylation levels. There are abundant *in vitro* investigations on the involvement of PECAM-1 in shear stress signal transduction. Early supporting data for the involvement of PECAM-1 in endothelial cell force sensing revealed that phosphorylation of PECAM-1, enrollment of Shp2 and Gab1, and phosphorylation of ERK accelerate in reaction to hypoosmotic shock and fluid shear stress (Osawa et al., 2002). In addition, the phosphatase activity of Shp2 is necessary for PECAM-1-driven ERK activation. Fyn has been identified as the kinase acting as a mediator of stretch- and shear stress-induced PECAM-1 phosphorylation, utilizing an extracted endothelial cell model (Chiu et al., 2008). Subsequently, PECAM-1 has been found to be involved with VE-cadherin and VEGFR2 in facilitating multiple reactions to shear stress. These reactions comprise endothelial cell orientation, NF-kB activation, and Akt phosphorylation induced by shear stress (Fleming et al., 2005; Tzima et al., 2005). Crucially, PECAM-1 is necessary for both anti-inflammatory and inflammatory signal transduction in endothelial cells (Tzima et al., 2005). PECAM-1 is needed for polarity determination in endothelial cells, following the onset of shear stress through the spatiotemporal activation of Rac GTPase (Liu Y. et al., 2013). In this model, PECAM-1 is necessary for the activation of Rac subsequent to the phosphorylation of Src and Vav2, whereas VE-cadherin serves as a framework for the localized activation of Rac at the endothelial cell downstream edge. Knockout of PECAM-1 also results in dysregulation of eNOS localization and a consequent rise in NO generation in non-stimulated endothelial cells (McCormick et al., 2011). Moreover, in endothelial cells exhibiting PECAM-1 knockout, the activation of eNOS is reduced in reaction to shear stress (Fleming et al., 2005).

Direct force exposure to PECAM-1 through ferromagnetic beads has permitted the investigation of specific PECAM-1-based signaling cascades. Early work using magnetic beads covered with PECAM-1 antibodies has revealed that direct force exposure to PECAM-1 triggers phosphorylation of both PECAM-1 and ERK (Osawa et al., 2002). The action of PECAM-1 is necessary for these reactions, underlining the significance of confluence in investigations of endothelial cell shear stress signal transduction. It has been demonstrated that a localized force directly exerted on PECAM-1 can activate overall cellular stiffening responses in endothelial cells. Force exerted on PECAM-1 with magnetic beads affects only a small region of the endothelial cell; nevertheless, global growth of focal adhesions and activation of RhoA are monitored, following 5 min of force exposure (Collins et al., 2012). The monitored growth of focal adherence junctions and RhoA signal transduction peaks in a stiffening of the cells in reaction to the exertion of force. Importantly, cellular stiffening, associated cytoskeletal dynamics, and focal adhesion growth are ECM-specific phenomena. When endothelial cells are seeded on collagen, the force-triggered growth of focal adhesion and cytoskeletal dynamics usually witnessed in endothelial cells cultured on fibronectin are impeded (Collins et al., 2014). This indicates that PECAM-1 and integrins work together in transducing subsequent signal transduction for force-driven processes. As the pharmacological blockade of PI3 kinase prevents force-based focal adhesion generation and the activation of integrins (Collins et al., 2012), PECAM-1 possibly engages in biochemical interactions with integrins rather than direct cytoskeletal force transmission.

PECAM-1-null mice remain healthy and fertile (Duncan et al., 1999), which is unexpected considering the involvement of shear stress signal transduction in vascular plexus formation. Starting at 4 weeks of age, nonetheless, PECAM-1-knockout mice exhibit cardiac deficiencies, comprising an enlarged left ventricular diameter, diminished fractional shortening, and decreased cardiac ejection fraction (McCormick et al., 2011). Blood flow-induced dilation is inhibited in *ex vivo* arteries of PECAM-KO mice because eNOS is not activated (Bagi et al., 2005). Moreover, PECAM-1 KO mice undergoing regional carotid artery ligation exhibit defective flow-based vascular remodeling and intima-based thickening because of deficiencies in the NF-kB signal transduction route (Chen and Tzima, 2009). PECAM-1 KO mice are also characterized by less collateral rearrangement, following ligation of the femoral artery, which again is attributed to decreased NF-kB-dependent transcriptional activity (Chen Z. et al., 2010). The results of atherosclerosis experiments with PECAM-1-knockout mice are challenging to understand, perhaps due to variations in the atherogenic genetic background and the period of feeding. PECAM-1-knockout mice, by contrast, have been found to exhibit less atherosclerotic plaque buildup in the smaller curvature of the aortic arch (Goel et al., 2008; Harry et al., 2008; Stevens et al., 2008; Harrison et al., 2013). This region is permanently subjected to perturbed flow, indicating that silencing of PECAM-1 attenuates the inflammatory signaling of the endothelial cells caused by the perturbed flow (Figure 4).

#### 3.4.2 VE-cadherin

When VE-cadherin-expressing cells are pooled with VE-cadherin-knockout cells and subjected to the flow, the VE-cadherin-expressing cells align toward the flow, while VE-cadherin-knockout cells fail to do so (Tzima et al., 2005). Endothelial cells align properly even in the complete lack of homophilic VE-cadherin adhesion. This indicates that VE-cadherin functions as an adapter in shear stress signal transduction. VE-cadherin knockout also prevents integrin activation, following the imposition of shear stress; however, it does not impede the activation of Src (Tzima et al., 2005). Src activation occurs immediately after phosphorylation of PECAM-1 and is among the earliest steps in shear stress signal transduction. This implies that VE-cadherin operates downstream of PECAM-1 in shear stress signal transduction, enabling VEGFR2 transactivation and subsequent signaling pathway initiation (Tzima et al., 2005; Liu et al., 2008). The transmembrane domain of VE-cadherin appears to be also necessary for the enrollment of VEGFR2 to the mechanosensory system, which further implicates VE-cadherin being an adapting molecule in this network (Coon et al., 2015). Emerging indications point to the potential for VE-cadherin to also be a mechanosensitive protein. Previous research indicates that exerting force on VE-cadherin utilizing antibody-coated magnetic beads fails to activate established force-sensitive signaling cascades; nevertheless, more recent findings indicate that this phenomenon could be epitope-specific (Tzima et al., 2005; Barry et al., 2015). Exposure of VE-cadherin to force utilizing the VE-cadherin FC antibody triggered reactions resembling those induced via force on PECAM-1, specifically cell stiffness and rearrangement of actin (Barry et al., 2015). Moreover, FRET experiments employing PECAM-1 and VE-cadherin tension sensors indicate that VE-cadherin bears a high-tension load in non-stimulated endothelial cells. This stress decreases as soon as a shear stress is imposed. Curiously, the tension on PECAM-1 is extremely low before shear stress is exerted. Following the introduction of shear, tension on PECAM-1 rises due to its engagement with vimentin (Conway et al., 2013). The primary function of VE-cadherin in shear stress signaling is probably to act as an adapting factor. The recent reports referred to earlier, however, indicate that VE-cadherin may also play additional functions in shear stress signal transduction in endothelial cells. Further investigations are required to ascertain whether the force-measuring characteristics of VE-cadherin are pertinent to physiological signal transmission.

#### 3.4.3 VEGF receptor 2

VEGF receptors 2 and 3 (VEGFR2 and VEGFR3) are likewise implicated in the perception of shear stress. The ligand VEGF and one of its receptors VEGFR-2 are the master regulatory agents in angiogenesis (Miller and Sewell-Loftin, 2022); nevertheless, VEGFR-2 is recognized to be activated, regardless of whether its ligand is bound or not (Jin et al., 2003; Warren et al., 2014). Multiple studies have demonstrated that mechanical forces can modulate the expression and activation of VEGFR-2 (Chen et al., 1999; Jin et al., 2003; Chen T. T. et al., 2010; Tian et al., 2016; LaValley et al., 2017; Vion et al., 2021; DeCastro et al., 2022). For instance, endothelial cells subjected to shear stress comprised the creation of a VEGFR-2 and VE-cadherin-β-catenin complex, which served as a mechanotransducer and permitted the cells to activate subsequent signal transduction pathways like p38 and Akt (Shay-Salit et al., 2002). VEGFR2, which appears to be the more well-defined of the two VEGFRs, is quickly phosphorylated at the initiation of shear stress exposure (Shay-Salit et al., 2002; Tzima et al., 2005; Liu et al., 2008). Shear stress-derived activation of Akt is inhibited when tyrosines 801 and 1,175 of VEGFR2 become mutated, indicating that VEGFR2 is necessary for shear stress-based PI3-kinase activation and the consequent subsequent signal transduction process. Therefore, although there is a lack of proof that VEGFR2 is directly mechanosensitive, its activity is necessary for proper shear stress signal transduction. Two further members of the VEGF receptor family are VEGFR-1 and VEGFR-3, both of which also engage members of the VEGF family (Takahashi and Shibuya, 2005). Neuropilin-1 (NRP-1) and neuropilin-2 (NRP-2) function as co-receptors for members of the VEGF receptor family and are proven to be involved in vascular growth, in developmental angiogenesis, and tumor-driven angiogenesis (Gelfand et al., 2014; Melincovici et al., 2018; Luo et al., 2020). VEGFR-1, similar to VEGFR-2, modulates angiogenesis, through the repression of pro-angiogenic signal transduction caused by the kinetics of engagement with the VEGF ligand. The extracellular domain of VEGFR-1 exhibits a higher affinity for VEGF relative to VEGFR-2, and there is emerging support that this linkage is involved in the angiogenesis restraint; however, VEGFR-2 displays higher tyrosine kinase activity, which leads to its greater involvement in angiogenesis (Takahashi and Shibuya, 2005; Koch and Claesson-Welsh, 2012; Melincovici et al., 2018). Alternative splicing can result in a soluble VEGFR-1 (sVEGFR-1) that lacks a transmembrane or intracellular domain, and it has been demonstrated that this variant inhibits the proliferation of vascular endothelial cells (Kendall and Thomas, 1993). Conversely, VEGFR-3 is generally linked to the lymphatic system development or lymphangiogenesis, although it is implicated in controlling some other angiogenic characteristics like VEGFR-2 expression (Mäkinen et al., 2001; Veikkola et al., 2001; Heinolainen et al., 2017). In a similar fashion to VEGFR-2, VEGFR-3 can be mechanically activated under sheer stress and produces complexes with VEGFR-2, PECAM-1, and VE-cadherin at the plasma membrane (Coon et al., 2015). These supporting data also suggest that VEGFR3 is involved in the perception of distinct shear forces, which advances the understanding of how distinct shear profiles are perceived. Upon initiation of shear, VEGFR2 and VEGFR3 are attracted to the mechanosensory complex through the transmembrane domain of VE-cadherin, which is immediately succeeded by the phosphorylation of VEGFR2 and VEGFR3 (Coon et al., 2015). There is also indication that VEGFR2 and VEGFR3 respond in a redundant manner to shear stress signals. Silencing both proteins, and not VEGFR2 or VEGFR3 separately, inhibits shear-driven Akt activation (Coon et al., 2015). Intriguingly, VEGFR3 has been involved in assessing the sensitivity of various types of endothelial cells toward shear stress (Baeyens et al., 2015). For example, human dermal lymphatic endothelial cells (HDLECs) and HUVECs exhibit tip alignment at various shear intensities, when subject to a series of shear stresses. HDLECs orient themselves most efficaciously at 5 dynes/cm^2^, whereas HUVECs orient themselves most efficaciously at 10 dynes/cm^2^. Moreover, HDLECs express larger amounts of VEGFR3 compared to HUVECs. HDLECs replicate HUVECs’ phenotype after VEGFR3 silencing and align at higher shear forces relative to normal HDLECs. Collectively, these findings imply that the perception of shear stress could be more diverse in the phenotypic landscape of endothelial cells than initially envisioned. Endothelial mechanosensitivity might not just be an efficient transmission of force and biochemical communication of information through a specialized endothelial cell protein(s), whose mechanisms are the same in all endothelial cells. Mechanosensing mechanisms can differ depending on the endothelial specific niche conferred by the varying expression of mechanosensory components.

#### 3.4.4 Tie receptors and angiopoietin 1

The endothelial TIE1 and TIE2 receptor tyrosine kinases constitute a dedicated subfamily identified through their unique extracellular domains. Tie family members of receptor tyrosine kinases have also been associated with the perception of shear stress (Chen-Konak et al., 2003; Porat et al., 2004). Tie1, which is a tyrosine kinase containing immunoglobulin-like and EGF-like domains 1, is expressed within endothelial cells, and its expression is clearly found in areas subjected to perturbed shear stress (Woo et al., 2011). Its expression is downregulated under laminar shear stress *in vitro*, whereas the perturbed flow enhances the activity of the Tie1 promoter. Deletion of Tie1 augmented eNOS activation and reduced inflammatory signaling in direct reaction to laminar flow *in vitro*. The same group detected a dose-driven decrease in atherosclerosis in Tie1-attenuated ApoE^−/−^ mice, supporting the concept that Tie1 is a crucial controller of the endothelial reaction to perturbed shear stress (Woo et al., 2011). Although it is mechanoresponsive that it changes its expression and thereby alters downstream signal transduction, it is uncertain whether it acts as a direct sensing element for shear stress. The ANG receptors TIE1 and TIE2, which are synonymously referred to as TEK, constitute a small subfamily of growth factor receptor tyrosine kinases (Saharinen et al., 2015). TIE1 and TIE2 are expressed nearly entirely in the endothelium, but there is also evidence of expression in hematopoietic cells, like M2 monocytes (Skiba et al., 2022). Along with angiopoietin growth factors, such as ANGPT1, ANGPT2, and ANGPT4, which is also shortened as ANG, the TIE receptors constitute an endothelial-specific signal transduction pathway with key roles in the control of lymphatic and cardiovascular development and in maintaining vascular homeostasis. The growth factors angiopoietin-1 (ANG1), ANG2, and ANG4, which comprise the human ortholog of mouse ANG3, are the ligands for TIE2 (Suri et al., 1996; Maisonpierre et al., 1997; Lee et al., 2004), while TIE1 is an orphan receptor that can still be triggered by ANG proteins through its engagement with TIE2 (Marron et al., 2000). Angiopoietins are present in multimeric versions that trigger TIE receptors through distinct mechanisms. Upon an endothelial cell–cell interface, angiopoietins stimulate the generation of homomeric in trans-TIE receptor complexes spanning the cell junctions, while matrix-bound ANG1 induces TIE receptor activation in a cis configuration. Compared to vascular endothelial growth factor receptors, TIE receptors are only slightly catabolized after activation by ubiquitin, while TIE2 signaling is negatively controlled via the vascular endothelial protein tyrosine phosphatase VE-PTP. ANG1 activation of TIE2 promotes stabilization of vessels, while ANG2, a context-dependent mild TIE2 agonist/antagonist, enhances abnormal tumor angiogenesis, permeability of vessels, and inflammation. ANG2 has been shown to convey some of its vessel-destabilizing and angiogenic roles through integrin signal transduction. Circulating ANG2 concentrations are increased in cancer and in several human diseases accompanied by inflammation and vascular leakage, such as sepsis. ANG2 blockade has proven to be a promising new therapeutic option for these diseases. Moreover, preclinical findings demonstrate that genetic deletion of TIE1 in mice retards vascularization and growth of tumor isografts and prevents atherosclerosis, with minimal impact on normal vascular homeostasis of adult mice (Saharinen et al., 2015). The ANG-TIE pathway’s capacity to regulate vascular stability and angiogenesis renders it an attractive vascular objective for the therapy of several diseases (Saharinen et al., 2011; Saharinen et al., 2015; Saharinen et al., 2017).

#### 3.4.5 Plexin D1

A novel membrane receptor, namely, plexin D1 (PLXND1), has been identified on endothelial cells that may operate as a mechanoreceptor for sensing forces (Mehta et al., 2020). More specifically, PLXND1 controls the functionality of the vessels and is responsible for the location-specific spread of atherosclerosis. Plexins are generally plasma membrane counterreceptors for semaphorins and are implicated in the axonal guidance, cancer propagation, and immune cell control (Valentini et al., 2021). In addition to its function as a counterreceptor for semaphorin 3E, PLXND1 is capable of perceiving physical forces that are translated into biochemical cues within the cell that subsequently determine the formation of atherosclerotic sites. In general, plexins are critical cell surface receptors belonging to the semaphorin family of signaling molecules and can govern the architecture of cells through modulation of the cytoskeleton and focal adhesion (Sakurai et al., 2010; Aghajanian et al., 2014). First, based on *in vitro* and *in vivo* models, it has been revealed that PLXND1 is necessary for the endothelial cell reaction to shear stress and that PLXND1-based mechanotransduction is not reliant on its ligand semaphorin 3E. Under laminar flow regimes, PLXND1 is necessary for the orientation of endothelial cells in the forward flow direction, which represents a characteristic reaction toward atheroprotective shear stress, and for the elevated expression of Klf2 and Klf4 that encode anti-inflammatory transcription factors. In disturbed flow regimes, PLXND1 is implicated in the elevated expression of pro-inflammatory genes, which include *Ccl2* and *VCAM-1*. PLXND1 has been found to control the site-specific spread of atherosclerotic plaques. In atheroprone mice on a high-fat diet, a lack of PLXND1 in endothelial cells reduced aortic plaque load in the entire aorta and in atheroprone areas, such as the aortic arch, when compared to mice expressing wildtype levels of PLXND1 in ECs. PLXND1 lack resulted in a decreased concentration of CCL2 and VCAM1 in the inside bend of the aortic arch. Finally, PLXND1 was found to act as a direct force sensor that can trigger mechanical responses in endothelial cells, and the mechanism by which a single receptor can have a binary function was uncovered. In specific detail, PLXND1 builds a complex with neuropilin 1 and VEGFR2 in reaction to the fluid flow. This complex functions by acting upstream of the PECAM-1 junctional compound complex and integrins, which comprise two mechanosensory hubs participating in cell–cell and cell–extracellular matrix attachment, and the tri-complex can react to shear stress. PLXND1 fulfills its binary functions as a ligand receptor and force receptor, in which it alternates between two conformations: a ring-shaped state preserves its ligand-dependent features, while flexion of the ectodomain yields a more open configuration necessary for mechanotransduction.

Multiple mechanosensors have been found in the endothelial cell, and among the most well-described mechanosensors is the PECAM-1 complex used for mechanosensing. These studies, nonetheless, failed to provide structural insights into the way the receptors exert mechanosensitivity, their role in disorders, and how they interact with the other established mechanosensors.

#### 3.4.6 Future perspective of junctional proteins as mechanosensors

The findings obtained for endothelial cells may be transferable to epithelial cells exposed to mechanical cues at interfaces. Moreover, the identification of mechanosensors on endothelial cells may help understand the interaction between the endothelium and immune cells or cancer cell transmigration. Apart from adherence junctions, tight junctions and gap junctions may act as mechanosensory proteins. Thus, elevated research effort is needed to fully reveal the function of these two other types of endothelial cell–cell junctions in mechanosensation. Endothelial mechanosensing is certainly essential for vascular functions. In this conjunction, it is important to recognize that the mechanosensory system of endothelial cells is by no means a uniform system. Instead, there exists a diverse nature of endothelial mechanosensing. The perception of hemodynamic forces involves several components: first, mechanosensors, some of which function in tandem and others that act as possible alternative or counter mechanisms; second, signaling proteins that act as regulators or amplifiers; and third, multiprotein assemblies that operate as integrated entities. The mechanotransduction mechanisms of the endothelium include fast reactions, such as acute alterations in vessel diameter, or lingering responses, involving alterations in the cytoskeleton of endothelial cells, or perhaps speculating on various physiological settings where Piezo1 is involved in vascular functioning and blood flow management. Mechanosensory elements can act together and can operate differently due to their location. Endothelial cells can even switch their phenotype upon mechanical stimulation. When examining mechanosensing and mechanotransduction, the physiology is complicated and sometimes enigmatic. The solution requires a comprehensive approach that recognizes that unrelated mechanosensors share downstream effectors and can, therefore, work together. Mechanosensors additionally transmit signals via molecules that reinforce or inhibit the activity of other mechanosensors. The concept also acknowledges the physical connection or colocalization of mechanosensors and mechanotransducers, whereby a common characteristic is the ability of various mechanosensors to trigger the exact same effector.

### 3.5 Membrane structures function as mechanosensors

Several protein microdomains are incorporated in the plasma membrane of endothelial cells. These microdomains endow the various areas with unique mechanical characteristics and thus lead to the heterogeneity of endothelial cells. Exposure of endothelial cells to shear forces generates a buildup of forces in plasma membrane areas with higher mechanical strength, such as focal adhesion. The latter results in the formation of regions with high strain or rigidity, which can function as mechanosensors. Other membrane structures that can be considered mechanosensors are the glycocalyx and caveolae.

#### 3.5.1 Glycocalyx acts as a mechanosensor

Endothelial cells possess a thick pericellular coating that is abundant in carbohydrates termed the glycocalyx. The endothelial glycocalyx functions as a critical interface that regulates the interaction of cells with multiple components of the microenvironment (Purcell and Godula, 2019). The endothelial glycocalyx acts in the apical mechanosensory system. The endothelial glycocalyx consists of a gel-like coating that covers the luminal surfaces of all endothelial cells. In the endothelial glycocalyx, the carbohydrates are linked to glycoproteins and proteoglycans (PGs), which are either sequestered or attached to the plasma membrane (Sprovieri and Martino, 2018). Due to its composition of glycosaminoglycans, proteoglycans, and glycoproteins, as well as its ability to adsorb proteins of the blood plasma (Weinbaum et al., 2007), it was first assumed that the glycocalyx acts as a structural shield and has been referred to as a permeability barrier (Dull and Hahn, 2022).

Determining the structure of the glycocalyx continues to be a difficult endeavor as the physical characteristics appear to vary according to a range of parameters, which includes the species and vascular cradle, resulting in a broad spectrum of assumptions about the thickness and architecture of the glycocalyx (Fronius, 2022). Most of these reciprocal effects are conveyed through the chondroitin sulfate (CS), glycosaminoglycans (GAGs), HA, and heparan sulfate (HS). These GAGs are linked to the PGs of the glycocalyx via covalent linkages, like HS and CS, or via weak binding interactions with non-PG membrane glycoproteins, like HA. PGs, which form a subclass of glycoproteins, have different HS/CS transport properties with different amounts of covalently bound HS and CS, according to the specific PG and PG isoform. The proportion of HS to CS chains in the vascular endothelium is about 4:1, and the amount of sulfation within each chain can also differ based on the underlying cellular microenvironment. In contrary to the sulfated GAGs HS and CS, HA misses sulfation and builds linear polymer chains that meander through the other constituents of the glycocalyx and attach themselves only weakly to certain membranous adhesion glycoproteins (Gao and Lipowsky, 2010; Kolářová et al., 2014). The availability of monosaccharides and the extent of enzymatic glycan activity finally govern the make-up of the glycocalyx, which is constantly changing and contributes to the diversity in building a “homogeneous” glycocalyx structure (Becker et al., 2015). Nevertheless, it has been proven that proteins can utilize saccharide-rich structures of the glycocalyx for several purposes. These involve adhesion to tissue and detection through the immune system, the adjustment of microvascular tonicity, and permeability of the endothelium, as well as the inhibition of excessive inflammation and coagulation reactions. Moreover, these molecular remnants have a decisive function within glycoproteins as they enable proper membrane folding and arrangement (Suzuki and Fujihira, 2021). The endothelial glycocalyx plays numerous roles in the vascular network, extending from the regulation of vascular tonicity to immune response and coagulation of blood. These functions comprise, first, the regulation of the coarse permeability of the vascular wall; second, the modification of the reciprocal exchange between endothelial cells and flowing circulation cells; and third, the perception and transmission of shear forces to endothelial cells, which—in the majority of instances—favors vasodilation (Bai and Wang, 2012). The glycocalyx serves as the principal mechanosensor. Nevertheless, the sole purpose of triggering the countless cell events that arise as a reaction to mechanical forces on the membrane cannot be the mere stimulation of the glycocalyx. Therefore, the force needs to be transferred to other proteins that probably engage in a direct interaction with the glycocalyx and experience conformational shifts as a function of physical disturbances of the glycocalyx, thereby eliciting stimuli for certain cell signaling phenomena (Askari et al., 2023). The glycocalyx has been proposed to fulfill a critical mechanosensing function (Zeng et al., 2018). The glycocalyx acts as an interface between the flowing blood and endothelial cells and transforms mechanical signals into intracellular cues. The glycocalyx engages with mechanosensitive ion channels such as ENaC (Reitsma et al., 2007; Fels and Kusche-Vihrog, 2020) and participates in shear-induced signal transduction routes that implicate endothelial cell junctional proteins like PECAM-1 (Kršek et al., 2024). The glycocalyx is also an important participant in shear stress-driven proteoglycan spreading and the expression of caveolin-1 (CAV1) (Ebong et al., 2014).

The glycocalyx, a layer of sialic acid and glycosaminoglycans, containing hyaluronic acid (HA) and heparan, is attached to the apical membrane through the HA receptor (Aruffo et al., 1990) and transmits shear stress sensing through endothelial cells (Mochizuki et al., 2003; Pahakis et al., 2007). Earlier work has shown that the glycocalyx is disrupted under disturbed flow regimes, undergoes erosion in the internal aortic arch, and modulates CAV1 expression and eNOS signal transduction (Harding et al., 2018). Additionally, it has been proposed that enzymatic cleavage of heparan sulfate fosters the flow-facilitated angiopoietin-2 expression through the AMPK/FoxO1 signaling route, which does not rely on KLF2 (Richter et al., 2022). A signaling cascade was identified that connects the glycocalyx, apical CD44 expression, and the cytoskeletal network of intracellular spectrin filaments, which governs the orientation of endothelial cells in response to shear stress (Mylvaganam et al., 2022). This orientation is junction-independent as subconfluent endothelial cells orient under *in vitro* flow situations, a behavior that necessitates HA, glycocalyx, CD44, and the spectrin scaffold. The importance of the integrity of the spectrin reticulum for preserving the orientation of aortic endothelial cells *in vivo* was also confirmed in the same investigation. In addition, shear stress has been shown to impact plasma membrane tension, which consequently induced caveolae-localized Piezo1 activity and the inflow of Ca^2+^ ions that was needed for downstream mechanosignaling and alignment of endothelial cells (Mylvaganam et al., 2022). This result raises new questions about the glycocalyx and the HA–CD44–spectrin–mechanosensor complex. Moreover, shear-induced generation of NO is controlled by spectrin (Mylvaganam et al., 2022). In the vasculature, NO is produced by eNOS that interferes with CAV1 under basal conditions. CAV1 keeps eNOS inactive through its sequestering via caveolae. Elevations in cytoskeletal Ca^2+^ levels lead to the activation of calmodulin that complexes with Ca^2+^ and tethers with eNOS, which perturbs its inhibitory interference with CAV1 (Schlessinger, 2000). When eNOS is separated from caveolae, it can be phosphorylated and hence activated (Noordeen et al., 2006; Mihai et al., 2009; Carafoli et al., 2012; Su et al., 2021). An apical spectrin scaffold is crucial for transmitting the force exerted by shearing on endothelial mechanosensors. When CD44 is attached, spectrins regulate the cell surface density of hyaluronan and sense shear stress and convert it into alterations of the plasma membrane tension. Spectrins also stabilize apical caveolae, which are likely to contain PIEZO1 mechanosensitive channels. Consequently, the shear-facilitated PIEZO1 activation and the related calcium influx were lacking in cells deficient in spectrin. Subsequently, cell alignment and blood flow-based endothelial nitric oxide synthase stimulation were found to be equally reliant on spectrin. Thus, the apical spectrin mesh is not just required for determining the shear force but also transfers and disperses the resulting tensile forces to mechanosensors that fulfill protective and vasoactive reactions.

Additional junction-independent processes may contribute to endothelial mechanotransduction. In this context, the glycocalyx has been associated with shear-induced reactions, and HA appears to be specifically important in this regard. HA, a non-sulfated glycosaminoglycan, is attached to the cell surface by attaching to its receptors, mainly CD44 (Coelho et al., 2017). Enzymatic breakdown of HA *in vitro* and *in vivo* interferes with endothelial reactions to shear forces (Ngai et al., 2022; Wang et al., 2022c; Liu et al., 2023a). Mechanosensitive ion channels, including PIEZO1, are also involved in endothelial responsiveness toward shear stress (Leitinger, 2014). In this way, several apparently unrelated molecules work together to produce endothelial reactions to the blood flow. However, there is still no coherent mechanism that reveals the coordination of the elements.

#### 3.5.2 Caveolae serve as mechanosensors

Caveolae are 50–100 nm small bottle-shaped indentations in the plasma membrane that are encrusted with cholesterol, caveolin, sphingolipids, and some other signaling molecules. Their creation and sustenance are based on a cholesterol-binding protein, CAV1, in non-muscle cells, and it is implicated in the control of multiple signaling pathways (Drab et al., 2001; Razani et al., 2001; Razani et al., 2002; Park et al., 2002). Caveolae are engaged in several vascular cell behaviors, and endothelial caveolae are probably implicated in mechanosensation and are, therefore, considered mechanosensory elements. Thereby, caveolae have been shown to alter their assembly state, the structural architecture of their membrane liquids, and/or activate mechanosensory receptors in their caveolae membrane. Caveolae respond to mechanical disturbances by breaking down quickly and in a reversible manner, liberating membrane regions and molecules as they flatten (Tomita et al., 2024). In reaction to tension, caveolae liberate free caveolins, the basic component of caveolae, and thus function as a cushion for the membrane tension (Yu et al., 2006; Sinha et al., 2011). Liberated caveolins interfere with other signaling molecules to trigger the initiation of mechanosensing cues.

Spectrin is required for endothelial caveolae stabilization that govern PIEZO1 regulation. Consistent with this, immunofluorescence in wildtype endothelial cells showed substantial colocalization of PIEZO1 and CAV-1, which is a key constituent of caveolae. Since caveolae are stably maintained at the plasma membrane via associations with cytoskeletal proteins (Parton and Del Pozo, 2013), the impact of spectrin on caveolae can be assessed as it may indirectly affect PIEZO1 activity. Although they were still available, significantly fewer caveolae were seen on the surface of SPTBN1-KO cells. Enhanced surface tension at quiescence in SPTBN1-KO cells could be responsible for the loss of caveolae and thus cell flattening. Shear forces lead to caveolae flattening. Collectively, these data indicate that spectrin transmits forces that are perceived by HA to elicit alterations in plasma membrane tension that cause caveolae to flatten, which then activates the tension-sensitive PIEZO1. Spectrin is a protein that is essential for the integrity of apical caveolae in endothelial cells. As an essential membrane reservoir, caveolae provide cell deformability (Sinha et al., 2011) and have been suggested to be part of the shear force sensing apparatus (Parton and Simons, 2007). It was uncertain whether shear forces lead to caveolae flattening or whether they primarily act as mechanosensitive molecular organizers. Spectrin modulates PIEZO1 sensitivity and activation in a dual manner: by affecting caveolae density and sustaining relatively low membrane tension. These findings offer a preliminary unified picture of how shear stress signaling is integrated and relayed to intracellular target effectors. Glycocalyx constituents, particularly HA, appear to be the primary sensors for fluid shear, and CD44 appears indispensable to relay the messages to the subjacent spectrin network. The latter is also associated with caveolae and determines their stability and dispersion. A shift in the spectrin mesh could slightly change the curvature or tension applied to caveolae, thereby activating PIEZO1. The subsequent calcium influx, possibly together with a mechanical deformation of the caveolae, leads to the stimulated liberation and activation of eNOS. In this scheme, spectrin plays a twofold function: on one hand, it modulates the stability of glycocalyx constituents (most directly exposed to the fluid flow) and senses and spreading them; on the other hand, it regulates plasma membrane tension through the stabilization of curved membrane microdomains like caveolae to affect the activation of mechanosensitive ion channels.

Laminar shear stress (15 dynes/cm^2^) elevated the expression of CAV1 at the plasma membrane, which is implicated in inflammation, adhesion, and phagocytosis. CAV1 changes ERK and Akt signaling profiles and promotes the enrichment and activation of eNOS inside the caveolae (Rizzo et al., 1998b; Rizzo et al., 1998a). Caveolae are able to engage with focal adhesion molecules, and this interplay is implicated in flow-induced reactions (Sonveaux et al., 2004; Radel and Rizzo, 2005). In addition, caveolae link mechanical stress to the activation of integrins and thus control the early stages of the mechanosensing reaction (Lolo et al., 2022). These effects underpin the participation of caveolae in endothelial cells in mechanosensitivity. Caveolae are key regulators of some other proteins and signaling molecules, including those implicated in endothelial cell Ca^2+^ signal transduction. Candidate mechanosensors like GPCRs and TRPV4 are two examples, both of which are implicated in endothelium-based vasodilation triggered through shear stress (Frank et al., 2003; Saliez et al., 2008; Rath et al., 2012). Caveolae are engaged in flow-driven ATP liberation of endothelial cells, which, in turn, activates purinergic signal transduction to augment Ca^2+^ signals (Yamamoto et al., 2011). Whether the endothelial Piezo1 channel is controlled through CAV1 and whether Piezo1 is primarily distributed in the caveolae of endothelial cells are still uncertain. In summary, caveolae are implicated in facilitating endothelial mechanosensation and various mechanotransduction events. Thus, CAV1 modulates endothelial NO synthesis, the microvascular permeability, and remodeling (Murata et al., 2007). In addition to caveolins, several accessory proteins have been recognized as fundamental components for the development and functioning of caveolae. Cavin-1, for example, which is synonymously referred to as polymerase transcript release factor (PTRF), and cavin-2, known synonymously as serum deprivation protein response (SDPR), are both necessary for the generation and operation of caveolae (Murata et al., 2007). CAV1 and Cavin-1 are major constituents of caveolae, both of which interfere with caveolae and affect their assembly and stabilization. CAV1 is linked with pulmonary arterial hypertension (PAH). The bone morphogenetic protein (BMP) receptor type 2 (BMPR2) is found in the caveolae linked with CAV1 and is frequently mutated in PAH. BMP/Smad signal transduction has been observed to be reduced in pulmonary microvascular endothelial cells isolated from CAV1-knockout mice. In addition, hypoxia increases the CAV1/Cavin-1 engagement while it weakens the CAV1/BMPR2 engagement and BMPR2 membrane targeting within pulmonary artery endothelial cells (PAECs). Cavin-1 and BMPR2 are both connected to the scaffold domain of CAV1. Cavin-1 reduces membrane localization of BMPR2 through blocking the interplay of BMPR2 with CAV1 and decreases Smad signal transduction within PAECs. Moreover, knockdown of cavin-1 renders it resilient to CAV1-driven pulmonary hypertension *in vivo*. The interaction between cavin-1 and CAV1 has been found to mitigate BMP/Smad signal transduction and represent a very encouraging therapeutic target for PAH (Tomita et al., 2024).

It is possible that caveolae in endothelial cells operate as mechanosensors of shear stress to trigger a set of events that enhance NO generation and vasodilation, but instead, they trigger a structural type of reaction, following stretch. Since flattening occurs as an intrinsic feature of caveolae, it has been hypothesized that this is a rapid cell survival strategy that provides a broader endothelial cell surface area (Parton and Del Pozo, 2013; Parton et al., 2020). This is consistent with other studies that emphasize caveolae as a membrane resource (Dulhunty and Franzini-Armstrong, 1975; Sinha et al., 2011). Flattening activity has been demonstrated to degrade caveolar scaffold proteins and liberate them into the plasma membrane (caveolin) and cytosol (cavin) (Sinha et al., 2011). Beyond conformational modifications, such degradation has no apparent effect. It remains to be seen whether this results in any compensatory responses that encourage the development of caveolae because of their fewer abundance. Based on the breakdown of the caveolae, it was hypothesized that the subunits of the protein can carry out independent functions, such as interactions with other proteins (McMahon et al., 2019). Underpinning evidence for this is a study proving that dissociated cavin-1 can tie to a transcription factor famously referred to as a type I collagen promoter-binding factor (BFCOL1), which, in turn, increases type I collagen, which is a key driver of depositing of the ECM (Hasegawa et al., 2000). CAV1 is able to engage with different types of intracellular signaling molecules accumulated in caveolae, including G-protein-coupled receptors, tyrosine kinases, eNOS, and several members of the MAPK signaling route (Li et al., 1995; García-Cardeña et al., 1996; Huang et al., 1997; Engelman et al., 1998; Dudãu et al., 2020; Zhao et al., 2022). There is an agreement that these molecules act as key effectors in shear stress-based induction of the endothelial cell’s activity. In line with this, exposing cells to chronic shear stress has revealed enhanced tyrosine phosphorylation and activation of surface proteins associated with shear-responsive signaling cascades, such as Akt, eNOS, and ERK (Sun et al., 2002; Boyd et al., 2003; Rizzo et al., 2003; Sun et al., 2003).

Although three isoforms have been recognized in the vascular smooth muscle, CAV1 is the sole compound necessary for caveolae generation, and its removal decreased contractile responsiveness (Gosens et al., 2011). The absence of this isoform results in an inhibition of endothelium-based relaxation, contractility, and myogenic tone (Brozovich et al., 2016). CAV1 and caveolae functionally amplify signaling pathways by incorporating important molecular elements like ion channels, adapter proteins, and receptors into membrane microdomains (Fridolfsson et al., 2014). In addition, CAV1 can control the activity of several ion channels and enzymes. For instance, swelling-activated Cl^−^ flows are inhibited in cell strains deficient in CAV1, whereas the transient expression of CAV1 can restore the phenotype. Actin filaments are reported to bind to and support stabilization of caveolae since depolymerization of the cytoskeleton results in the migration of CAV1 across the cell (Fridolfsson et al., 2014). This connection establishes a highly efficacious mechanosensitive region, which is a localized zone where mechanical signals can be gated, perceived, and handled. This mechanism has been hypothesized for stretch-activated Ca^2+^ channels because they are located in caveolae, and their activity is closely governed through the actin cytoskeleton (Fridolfsson et al., 2014; Davis et al., 2023). CAV1 has been reported to engage directly with Kir2 channels, thereby reducing the current density without affecting the characteristics of individual channels or the expression of membrane proteins. In this respect, it has been revealed that CAV1 serves as a negative controller of Kir channel activity and that CAV1 and cholesterol function to stabilize the channel in a closed, quiescent condition through a common modulatory mechanism (Han et al., 2014), albeit CAV1 is not necessary to impart cholesterol sensitivity on the channel. Using crystallography, two presumptive CAV1-binding domains have been observed, whereby the first lies at the interface between the outer transmembrane helix and the N-terminus and the second is located in the outer transmembrane helix adjacent to the channels’ extracellular domain. In terms of cerebral circulation, it has been revealed that there is a tight connection (<40 nm) of Kir2 channel subunits and CAV1 located within caveolae formations in the membrane of cerebral vascular smooth muscle cells. Through this tandem arrangement, the mechanosensitivity of the Kir2 channel is potentially increased (Sancho et al., 2022). Importantly, simulated pressure utilizing a hypoosmotic stimulus challenge has been demonstrated to inhibit cerebral arterial smooth muscle cell Kir2 flows in whole-cell recordings, which was fully restored by pre-incubation with actin-disrupting agents, such as latrunculin A and/or cytochalasin D. Similarly, disconnection of caveolae-forming proteins hindered the inhibition of Kir2 smooth muscle flows during cell swelling. In addition, immunofluorescence assays revealed the expression of both scaffold proteins, syntrophin and CAV1, in vascular smooth muscle, and a proximity ligation assay emphasized the tight structural connection with Kir2.2 subunits, which was seen within 40 nm of one another, pointing to their participation in mechanosensing of the channel. These results offered convincing support that the mechanosensitivity of Kir2 channels entails distinct interactions with the cytoskeleton that are probably regulated by scaffold proteins.

Knockdown of CAV1 using siRNA (Wang et al., 2009) or caveolae abrogation by methyl-β-cyclodextrin (MβCD) abolished shear stress-driven ATP liberation and Ca^2+^ signal transduction (Albrakati, 2022). Laurdan imaging revealed that caveolar membrane domains react to laminar shear stress (15 dyn/cm^2^) through fast shifting their lipid-ordered configuration from the liquid-ordered state toward the liquid-disturbed state. Moreover, the addition of cholesterol to the endothelial cells inhibits the lipid-ordering reaction of the caveolae and significantly represses shear stress-driven ATP liberation (Parasassi et al., 1997). These results imply that the transition in the caveolar lipid order is engaged in mechanotransduction through shear stress, resulting in ATP liberation. The caveolae are likewise implicated in cellular reactions to stretching. A sustained 20% stretch induced activation of the small Rho family GTPases, such as RhoA and Rac1, in rat cardiomyocytes and then disengaged them from the caveolae. Caveolar disconnection with MβCD prevented stretch-induced activation of RhoA and Rac1 (Sinha et al., 2011). Subjecting mouse mesangial cells to cyclic 1 Hz loading for 10 min induced transactivation of the epidermal growth factor receptor (EGFR) and activation of protein kinase B (Akt). Caveolae disconnection with MβCD or filipin inhibited stretch-induced Akt activation, and both EGFR and Akt activation from stretch were abolished in mice with CAV1 knockout, implying that healthy caveolae are necessary for stretch signals to emerge (Luo et al., 2021).

#### 3.5.3 Primary cilia function as mechanosensors

The contributing function of cilia as endothelial sensors of fluid shear stress is poorly understood, although there is considerable evidence that cilia serve as primary sensors of the flow (Nauli et al., 2008; AbouAlaiwi et al., 2009). The presence of primary cilia on mammalian is still to be confirmed and under debate. Primary cilia appear to be deficient in calcification of mammalian endothelial cells (Sánchez-Duffhues et al., 2015). Shear stress distorts the cilia and initiates downstream calcium signaling necessary for endothelial responsiveness toward the flow (Goetz et al., 2014). In addition, endothelial cells have cilia with a specialized architecture that enables a flexible responsiveness to the flow (Goetz et al., 2014). In the mammalian aorta, primary cilia exist in areas of low disturbed flow (Van der Heiden et al., 2008) and control atherosclerosis (Dinsmore and Reiter, 2016). Recent studies also point to the fact that the deciliation of endothelial cells in high-flow settings could even be a possible biomarker for endothelial injury (Gupta et al., 2022).

Primary cilia consist of 3–5-μm-long cellular protuberances that are organized by microtubule bundles. In the embryonic phase, there is indication that cilia are needed in the evolving vessels, where the flow conditions are low. In a developing zebrafish, disruption of the intraflagellar transport protein Ift81 leads to cranial hemorrhages, whereas silencing of Pkd2, which is specific for endothelial cells, leads to hemorrhages in the skull and back of the developing mice (Sen-Banerjee et al., 2005; Parmar et al., 2006; Hamik et al., 2007; Garcia-Gonzalez et al., 2010; Kallakuri et al., 2015). Primary cilia of endothelial cells are mainly located in parts of the vascular network where the blood flow is low or disturbed. Their existence has been detected in embryonic endocardial and venous endothelial cells of the chicken (Van der Heiden et al., 2006). Cilia are present in the embryonic aorta of mice and in the inner curvature within the aorta in adult mice; both regions of the vascular system are characterized by a low flow (Nauli et al., 2008; Van der Heiden et al., 2008). Moreover, it has been found that zebrafish arteries and veins display cilia (Goetz et al., 2014). Cilia have been identified in primary chicken endothelial cells (Hierck et al., 2008), embryonic mouse aortic endothelial cells (Nauli et al., 2008), and HUVECs (Iomini et al., 2004). Laminar shear forces lead to fast breakdown of cilia in endothelial cells, so the potential role of cilia in prolonged shear stress signal transduction is still uncertain (Iomini et al., 2004).

Several research projects dealing with the reaction of cilia to shear forces indicate that cilia are involved in early calcium signal transduction and NO synthesis. Primary cilia cooperate with polycystin 1 and 2 (PKD1 and PKD2), which are expressed by the Pkd1 and Pkd2 genes, to transduce shear-induced calcium and NO signaling. PKD1, which represents a GPCR, activates Gi/o proteins in a constitutive manner. Physical linkages with PKD2, which belongs to the Ca^2+^ channels, impair this constitutive activation (Delmas et al., 2002). Microfluorimetry has been employed to investigate early shear-dependent calcium inward flow and NO generation *in vitro* in aortic endothelial cells of Pkd1^−/−^ mice. High levels of calcium influx and NO generation have been detected in wildtype cells 20 seconds after shear stress application (Nauli et al., 2008). Nevertheless, early calcium inward flow and NO synthesis have been obstructed in endothelial cells of Pkd1^−/−^ mice. Moreover, Pkd2 is necessary for the shear-triggered calcium inward flow and NO generation. The silencing of Pkd2 within endothelial cells suppresses these initial processes (AbouAlaiwi et al., 2009). Most interestingly, endothelial cells derived from a patient with autosomal dominant polycystic kidney disease exhibit heterogeneous expression of PKD2. In these patients, PKD2-null endothelial cells reveal abnormal eNOS positioning upon shear stress exposure (AbouAlaiwi et al., 2009). *In vivo* investigations on primary cilia add to the earlier *in vitro* investigations on cilia. A morpholino against Pkd2 inhibits the calcium inward flow into the arterial endothelium of zebrafish embryos (Goetz et al., 2014). There are also Tnnt2 and gata1 morpholinos utilized, which inhibit the heartbeat and decrease the hematocrit, respectively. Tnnt2 morphants show no deflection of the cilia, whereas the cilia in gata1 morphants are only mildly deflected. It is significant that the calcium entry into the endothelial cells relates to the level of deflection of the cilia as tnnt2 morphants exhibit a marked decrease in calcium entry into the endothelial cells, whereas gata1 morphants have a moderate decrease (Nauli et al., 2008; Goetz et al., 2014). Notwithstanding the reported evidence of cilia participation in Ca^2+^ and NO signal transduction, the role of primary cilia functioning as shear sensors is not yet clearly elucidated. Cilia are located in areas of the vasculature with low blood flow (Tzima et al., 2001; Li et al., 2005; Liu et al., 2008; Van der Heiden et al., 2008) and tend to be missing in high-flow zones. This raises the fascinating prospect that cilia generation is itself a flow-dependent event as cilia generate when blood flow is low or perturbed. Although this does not rule out cilia acting as mechanosensors, their function as reinforcers of shear signatures (Lan et al., 1994; Hierck et al., 2008; Cuhlmann et al., 2011) is surely coherent with their existence in regions of low blood flow. Cilia can function as a sensor of low flow in the endothelium, but the disentanglement of cilia roles in development and the overall shear signaling of endothelial cells continue to be a major effort.

#### 3.5.4 Cytoskeleton is engaged in endothelial mechanoresponse

It has been postulated that the cytoskeleton serves as an integrative mechanism to transmit forces from mechanosensory receptors to mechanosensitive molecular entities in cells (Davies, 1995). Most studies have concentrated on the actin cytoskeleton due to its fundamental involvement in controlling the activity of integrins and junctional protein complexes, which are frequently implicated in mechanotransduction (Hahn and Schwartz, 2009). Actin filaments, in contrast, cannot sustain large forces and are usually highly dynamic (Palmer et al., 1999). This indicates that actin-rich structures on their own are not well-adapted to serve as a framework for mechanosensitive cells. In addition, the hypothesis was put forward that the spectrin cytoskeleton, which includes only short, relatively stable actin filaments, is a basic integrator of mechanical signal transduction on the apical surface of the endothelium. Spectrins, found abundantly and highly conserved in the tissues of multicellular organisms, are involved in stabilizing cell membranes and regulating the activity of ion channels (Bennett and Healy, 2009). Albeit the underlying mechanism is still unclear, spectrins appear to be especially critical for vascular function. Spectrins are highly flexible and bind with short actin filaments, making them very stable. The ensuing spectrin–actin lattice is particularly flexible and durable (Lambert and Bennett, 1993). The flexibility imparts enhanced conformance and deformability to the membrane and is optimal for cells subjected to the dynamic vascular environment. In fact, the spectrin cytoskeleton is needed to preserve the integrity of the erythrocytes when they are put under hemodynamic stresses (Sheetz and Singer, 1977; Sheetz et al., 1980) and have to pass through narrow constrictions (Lux et al., 1976; Agre et al., 1982; Chasis et al., 1988). Endothelial cells are likewise exposed to hemodynamic stress, notably in the aorta, and in constricted capillaries, where the endothelium is distorted by streaming erythrocytes. Ultimately, it has been found that shear stress causes endothelial spectrin to reshape, a characteristic that is likely to be pivotal in vascular homeostasis (Mylvaganam et al., 2022).

## 4 Mechanical interplay between endothelial cells and vascular smooth muscle cells

**FIGURE 5:**
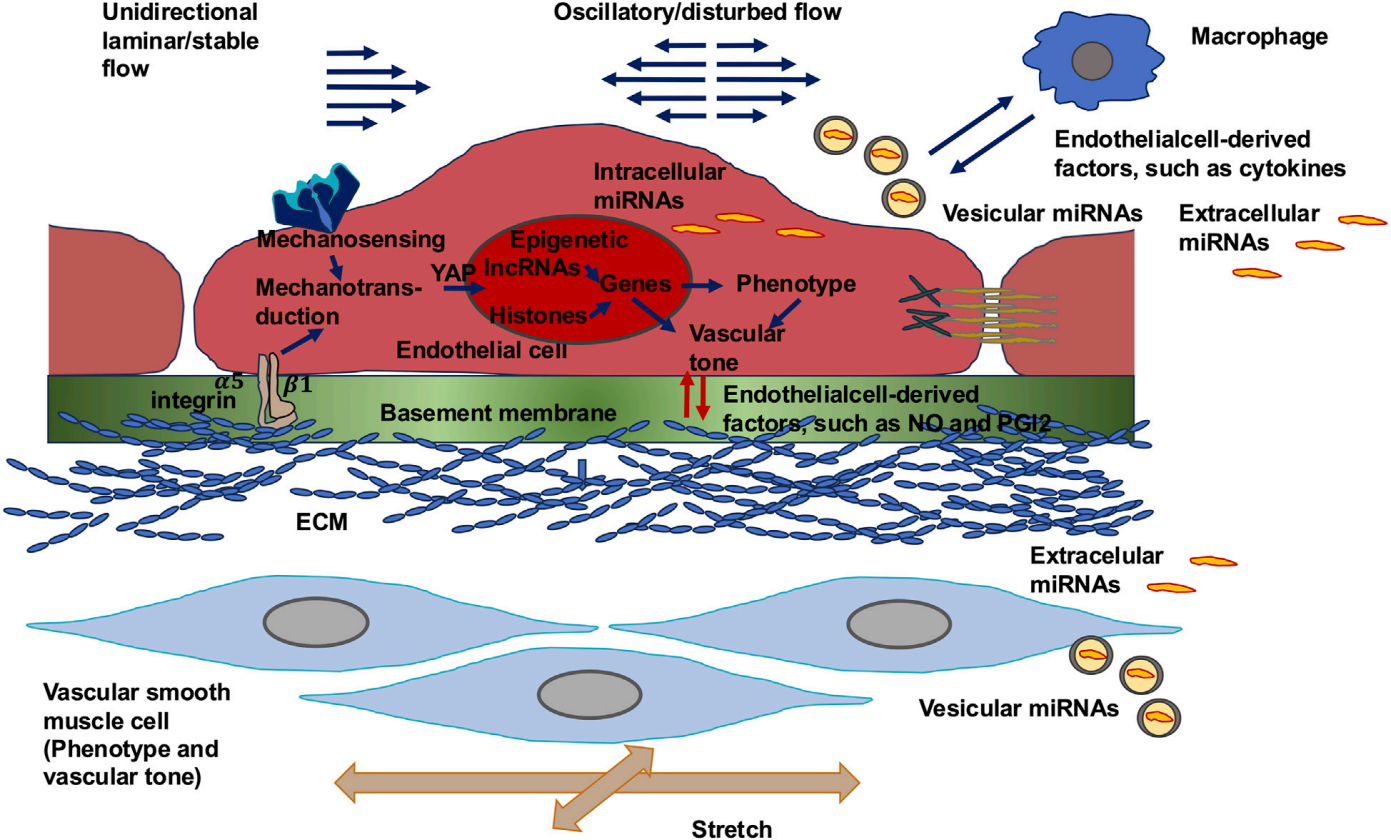
Vascular interference is regulated by shear flow. Shear stress can alter the endothelial cell function via endothelial mechanosensing and mechanotransduction events to govern the epigenome, transcriptome, phenotype, and interplay between endothelial cells and VSMCs and other adjacent cells, such as macrophages. The regulation of this interferences results in homeostasis under normal conditions, and in pathological conditions, it leads to diseases. lncRNAs, long non-coding RNAs.

Not only mechanosensory cells can interact but also different cell types such as endothelial cells and VSMCs can cooperate in mechanosensation, mechanotransduction, and vascular function. Resistance arteries make up small blood vessels that are less than 400 μm in diameter and, therefore, account for peripheral vascular resistance. The vessel wall of these blood vessels responds structurally and intrinsically (locally) to the constantly arising dynamic forces in such a way as to finally control the vascular tone and adjust the blood flow to the metabolic needs of the tissue. This capability is accomplished through the plasma membranes of two of the major parts of the vascular wall, such as smooth muscle and the endothelium, which are permanently subjected to mechanical irritations caused mainly by the pulsatile blood flow, including stretch (tensile stress) and/or shear stress (Lehoux and Tedgui, 1998). In this regard, certain mechanosensitive molecules located in the plasma membrane, such as ion channels, caveolae, and/or surface receptors, can change their conformational status and electrical/chemical characteristics in reaction to mechanical perturbations, transforming them into a physiological reaction (Ingber, 2006; Martinac, 2014). The endothelium is not a closed system in mechanical terms; it can interact with other nearby cells, such as VSMCs (Figure 5). Once the mechanical forces have been perceived from the endothelial cells, they are codified and passed on to the VSMCs, which react by either relaxing or contracting. A tight functional engagement between the endothelial cells and the neighboring VSMCs is necessary for the control of vascular tonicity and the capacity of the cells to respond to different biochemical and mechanical impulses received from the flowing blood. In recent years, it has been found that the mechanical properties of endothelial cells rely on flow-induced forces, on one hand, and govern the contractile nature of VSMCs, on the other. At the heart of this well-known process is the capacity of endothelial cells to liberate NO in accordance with shear stress, causing it to diffuse toward neighboring VSMCs, where it induces vasodilation through cGMP-dependent routes (Bolotina et al., 1994). A decrease in NO is closely related to elevated amounts of reactive oxygen species (ROS) produced by NAD(P)H oxidase, xanthine oxidase, or uncoupled endothelial nitric oxide synthase (eNOS) in the vessel wall, resulting not solely in a depletion of NO but also in an interruption of several signaling cascades that convey NO generation (Lubos et al., 2008). Therefore, the close communication between endothelial cells and VSMCs regulates vascular performance and vascular tonicity. An important physiological feature of the endothelium is mainly the capacity of endothelial cells to modify their mechanical characteristics, that is, to switch between “stiff” and “soft” states. Endothelial cells that have lost this capacity and are stuck in chronic stiffening can be considered dysfunctional (Marti et al., 2012). Moreover, the mechanoresponse involves the interaction of endothelial cells with VSMCs, stromal cells, and immune cells in the local vessel walls via microRNA-driven and extracellular vesicle-driven mechanisms (Tan et al., 2020; Li et al., 2022). Endothelial cells and VSMCs constitute the key adjacent wall cells that build barriers. They can exchange information through direct proximity, the transfer of signaling agents, and the accumulation of ECM. Their interaction is a key mechanism in the control of vascular tonicity. Endothelium-derived factors (PDGF-B, CXCL12, ET-1, and MIF) have been found to drive VSMC proliferation, thereby promoting vascular restructuring and pulmonary arterial hypertension (Dai et al., 2018). Consequently, the endothelial-specific alk5 overexpression in alk5^−/−^ has been hypothesized to reestablish VSMC proliferation in the wall of the coronary effluent tract (Boezio et al., 2020). Finally, the emphasis is the role of endothelial cells in the process of mechanosensing and mechanotransduction, and therefore, in the following, the three main types of endothelial mechanosensors are presented and discussed.

### 4.1 Epigenomic mechanoregulation of vascular smooth muscle cells

Epigenetic signatures characterize the non-genetic alterations to the genome through chemical changes to DNA and its associated proteins like DNA methylation and histone modifications (Rivera and Ren, 2013). These heritable alterations in expression are regulated through various chemical changes of bases in DNA, involving DNA methylation and histone modification, and non-coding RNA (ncRNA) regulatory mechanisms. In contrast to the majority of cell types, nevertheless, VSMCs are non-terminally differentiated and, therefore, exhibit phenotypic plasticity (Babaev et al., 1990). This plasticity varies widely from contractile-resting to migratory-proliferative-synthetic to osteogenic or macrophage-like cells (Jaminon et al., 2019). Endothelial cells may possibly alter the phenotype of VSMCs by changing their epigenetic signatures upon mechanical sensing and signaling, More generally, several genes that determine the VSMC phenotype have been identified to be governed through DNA methylation, involving SRF, platelet-derived growth factor B (PDGF-B) of the GATA-6-PDGF-B pathway in endothelial cells, and TAGLN (De Oca et al., 2010). Thereby, TET2 can serve as a key epigenetic modulator of the phenotype of VSMCs (Liu R. et al., 2013). In addition, knockdown of TET2 attenuates the expression of crucial VSMC genes like MYOCD and SRF with concomitant transcriptional upregulation of KLF4, which enhances pluripotency network reactivation and consequently phenotype transition. Conversely, overexpression of TET2 leads to a contractile VSMC phenotype, reestablishes the epigenetic 5-hmC map, and substantially decreases intimal hyperplasia *in vivo* (Liu R. et al., 2013). Post-transcriptional histone modifications comprise, among others, acetylation, methylation, and ubiquitinylation, which are primarily occurring at arginine (R) and lysine (K) residues (Lawrence et al., 2016). Usually, these changes can take place in combined form, like, for example, dimethylation (me2) or trimethylation (me3) at histone H3K4 in combination with H3K9ac or H3K14ac, all of which activate gene expression (Kimura, 2013). Acetylation by histone acetyltransferases (HATs) generally attenuates the DNA–histone interactivity, thereby rendering genes more amenable to transcription. In contrast, deleting an acetyl group by histone deacetylases (HDACs) reinforces the coupling between DNA and histone, leading to suppression of gene expression (Gomez et al., 2015; Jiang et al., 2018). Several alterations in the levels of histone methylation and acetylation have been determined to have a critical impact on the progression of diseases, such as atherosclerosis, and the transition of VSMC toward the synthetic phenotype, which involves a marked reduction in H3K9 and H3K27 methylation (Greißel et al., 2015) with a simultaneous rise in H3K9 and H3K27 acetylation in progressing atherosclerotic plaques (Greißel et al., 2016). The involvement of H3K27 methylation in the VSMC transition phenotype has been confirmed by experiments, in which a decrease of H3K27me3 in tunica media cells has a key function in the differentiation and proliferation of VSMCs in endothelial dysfunction like atherosclerosis (Wierda et al., 2015). Reduced levels of H3K9me2 have been detected in VSMCs in atherosclerotic lesions and arteries experiencing lesion-related restructuring and are linked to increased transcription of inflammation-responsive genes (Harman et al., 2019). Elevated expression of the histone demethylase KDM3a in diabetic rats has been demonstrated to promote neointimal hyperplasia by diminishing H3K9 dimethylation within the ROCK2 and AGTR1 sites, suggesting that the shift from the contractile to the synthetic VSMC phenotype is amplified through activation of the Rho/ROCK and AngII/AGTR1 signal transduction routes (Chen et al., 2017). Finally, several epigenetic alterations can either enhance or impede arterial restructuring in diseases like atherosclerosis by influencing directly and indirectly the VSMC phenotype. Several target genes and molecules that regulate all forms of epigenetic modifications have been evaluated in animal models and clinical studies for their efficacy in the general therapy of atherosclerosis (Jiang et al., 2018). For instance, the DNMT inhibitor 5-aza-2′-deoxycytidine (5-aza-dC) has inhibited the depressed expression of methylated genes (Mossman et al., 2010), reduced overall 5-mC levels, and re-established the expression of myocardin expression in VSMCs triggered through PDGF, which thereby impedes excessive dedifferentiation of VSMCs (Zhuang et al., 2017).

### 4.2 MIRs facilitate exchange between endothelial cells and vascular smooth muscle cells

MIRs can be secreted by endothelial exosomes, which are small extracellular vesicles of a diameter of 40–150 nm that emanate from endosomal multivesicular bodies (Stoorvogel et al., 2002; Tkach and Théry, 2016). Numerous studies have revealed that exosomes are key paracrine facilitators of intercellular signaling (Loyer et al., 2014; Wu et al., 2019) and modulate the functionality of recipient cells by transmitting various functional molecules, including proteins, lipids, and nucleic acids, such as miRNA, mRNA, and lncRNA (Hergenreider et al., 2012; Deng et al., 2015; Lin et al., 2019). Endothelial cells can sequester exosomal miR-143/145 clusters that are conferred to VSMCs and regulate atherosclerotic lesion development by acting on KLF2 (Hergenreider et al., 2012). Several studies have described the transfer of microRNAs, like miR-143/145, between endothelial cells and their connected VSMCs (Hergenreider et al., 2012; Climent et al., 2015; Deng et al., 2015). It has been observed that miR-143–3p regulates exosome-based intercellular communication between pulmonary arterial endothelial cells and VSMCs in the development of pulmonary arterial hypertension (Deng et al., 2015). Nevertheless, it is uncertain as to whether and in what way exosomes also facilitate endothelial cell–VSMC intercellular communication in diseases (Lin et al., 2022). Finally, a critical role of endothelial cell-derived exosomal miR-670–3p in the regulation of arteriosclerosis has been established. Therefore, it can be hypothesized that miR-670–3p secreted by endothelial cells could be a candidate target for the treatment and prediction of arteriosclerosis and other diseases. Both endothelial dysfunction and VSMC plasticity are key factors in the pathogenesis of hypertension and stiffness of arteries. MicroRNAs can facilitate cellular signaling between vascular endothelial cells and VSMCs. The role of endothelium-derived extracellular miR-92a in promoting arterial stiffness by modulating intercellular communication of endothelial cells and VSMCs has been investigated (Wang C. et al., 2022). The serum level of miR-92a has been seen to be elevated in hypertensive patients compared to control subjects. Circulating miR-92a levels correlated positively with pulse wave velocity (PWV), systolic blood pressure (SBP), diastolic blood pressure (DBP), and serum endothelin-1 (ET-1) levels, although inversely with levels of serum NO. *In vitro*, elevated levels of miR-92a in endothelial cells induced by angiotensin II (Ang II) caused a phenotype shift from contractile to synthetic in cocultured VSMCs. In mice infused with Ang II, a lock nucleic acid-modified antisense miR-92a (LNA-miR-92a) ameliorated Ang II-induced PWV, SBP, and DBP and compromised vasodilation. Application of LNA-miR-92a also completely reverted the elevated levels of proliferative genes and the reduced amounts of contractile genes triggered in the mouse aorta with Ang II. The circulating serum concentration of miR-92a and vascular stiffness were positively related in these mice. These results lead to the hypothesis that endothelial cell miR-92a can be delivered to VSMCs through extracellular vesicles to control phenotypic transitions of VSMCs, which consequently results in elevated arterial stiffness. Moreover, extracellular vesicles secreted by endothelial cells contributed to the contractile phenotype of VSMCs through the miR-206/ARF6&NCX1/exosome route (Lin et al., 2016). MiR-582 secreted by endothelial cells acts on VSMCs (Fontaine et al., 2021). The proliferation of VSMCs is also affected with miR-582 since this miRNA reduces the expression of CASP3 and PTPRJ (Woo et al., 2003; Smart et al., 2012). The regulation of ITGA3 and PDCD6 by miR-582, which is implicated in cell migration, could finally be affirmed (Su et al., 2012; Kurozumi et al., 2016).

### 4.3 Myogenic tone (pressure-driven constriction) control of vessels via vascular smooth muscle cells

**FIGURE 6:**
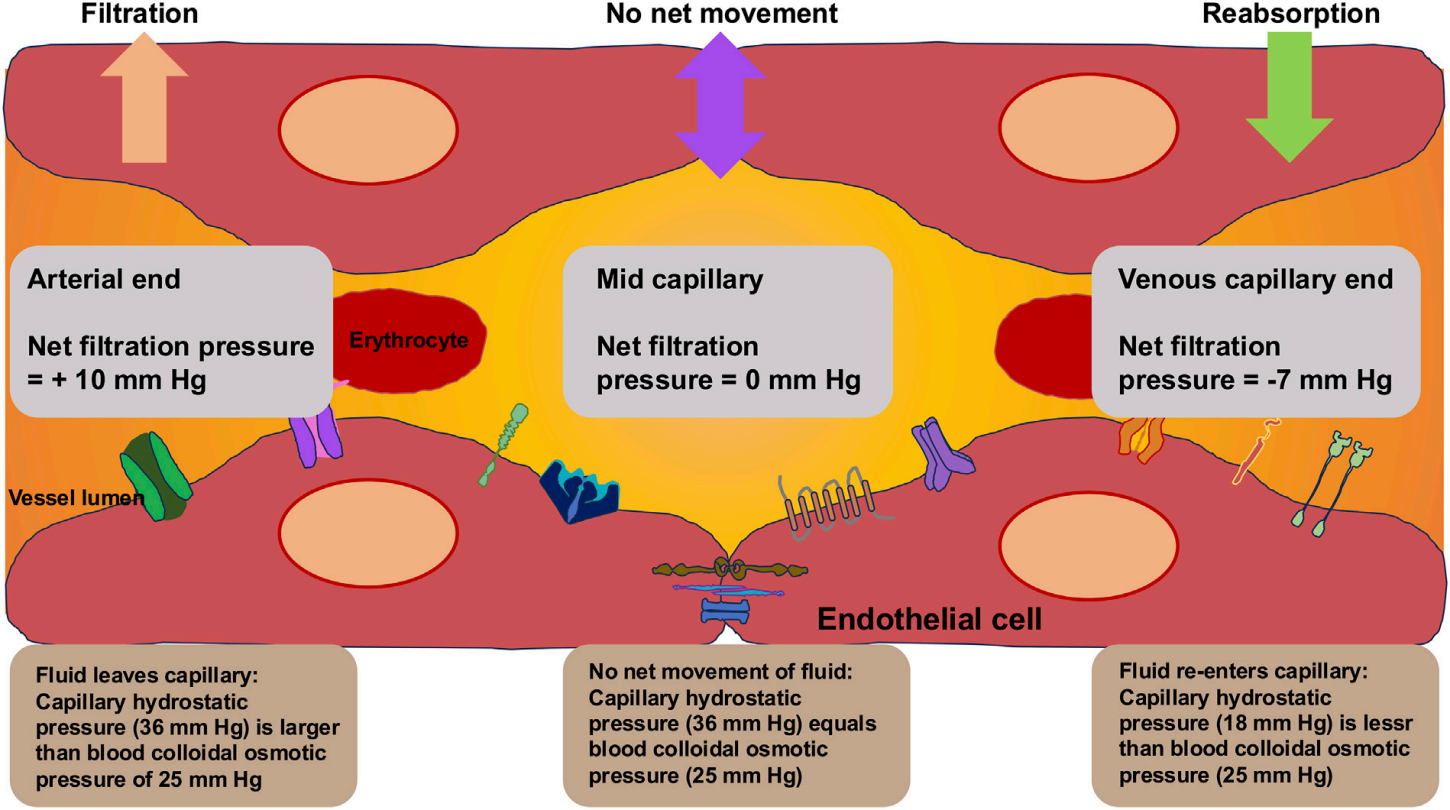
The net filtration takes place close to the arterial end of the capillary, when capillary hydrostatic pressure (CHP) is larger than the blood colloidal osmotic pressure (BCOP). There is no motion of fluid near the midpoint (CHP = BCOP). Net reabsorption takes place close to the venous end of the capillary (BCOP is larger than CHP).

The VSMC layer of a resistance artery or of arterioles penetrating inferiorly has an intrinsic capacity, which is regardless of the endothelium and nerves, to contract and decrease luminal diameter in reaction to an abrasive rise in transmural pressure, such as radial stretch (Bayliss, 1902). Within the brain, this process, termed ‘myogenic tone,’ enhances opposition to the blood flow (Davis and Hill, 1999; Kirby et al., 2007) and is critical for setting marrow basal vascular tone, sustaining steady perfusion across a wide array of intraluminal pressures and finely adjusting local CBF simultaneously safeguarding the downstream capillary meshwork against injury (Figure 6) (Hill and Davis, 2007; Cole and Welsh, 2011). Curiously, the reaction to myogenic tone gets stronger as the vessel size reduces (Russell et al., 1970; Davis, 1993), which may be attributed to an elevation in vessel wall distensibility with the ramification of the vascular tree (Thorin-Trescases et al., 1997). In addition, the myogenic tone of resistance arteries can be affected via hemodynamic forces, such as the flow, metabolic agents, and vasoactive modulators secreted by other cell types, such as the endothelium (Bevan and Joyce, 1990; Garcia-Roldan and Bevan, 1990; Ngai and Winn, 1995). The mechanisms behind this phenomenon have been thoroughly investigated (Davis and Hill, 1999; Welsh et al., 2000; Hill et al., 2001; Osol et al., 2002; Brayden et al., 2008; Sharif-Naeini et al., 2010). An elevation in intraluminal pressure acts to modify the activity of stretch-sensitive ion channels that are expressed in VSMCs, leading to membrane depolarization and the engagement of voltage-gated calcium channels (VGCCs), resulting in elevated intracellular levels of Ca^2+^ ions (Knot and Nelson, 1998; Thorneloe and Nelson, 2005). Therefore, mechanosensitive ion channels in VSMCs are frequently studied because they function as pressure sensors through depolarizing the arterial membrane potential (V_M_) and triggering the myogenic reaction (Davis and Hill, 1999; Welsh et al., 2002). Cerebral myogenic reaction is essential for accurate VSMC functionality, and consequently, disruption of myogenic tone has been linked to multiple vascular diseases, like stroke, hypertension, and dementia (Izzard et al., 2003; Toth et al., 2013; Toth et al., 2017).

## 5 Endothelial mechanosensing in pathological settings, such as cancer

The process of mechanosensing involves a force and a sensor. When one or both are changed, mechanosensing and/or mechanotransduction is compromised, causing endothelial malfunction. Disturbances also arise in ambiguous circumstances, for example, in collateral vessels and in cases of ischemia-reperfusion. At the same time, mechanosensors are susceptible to post-translational alterations and mutations that impair their performance. In the following, pathologies associated with altered mechanosensing are briefly presented with emphasis on cancer.

### 5.1 Flow pattern regulates the signal transduction processes in endothelial cells

Hemodynamic disturbances may cause pathologic results rather than physiologic reactions. Atherosclerosis is a prime illustration of changing forces acting on endothelial cells. Atheroprotective or atherogenic endothelial signals mark vascular areas in relation to blood flow profiles (Albarrán-Juárez et al., 2018). Blood vessels with a high laminar flow show an orientation of endothelial cells in the direction of the flow, an anti-inflammatory phenotype, reduced oxidative stress, turnover of cells, and permeability (Hahn and Schwartz, 2009). In contrast, vascular areas with a low or oscillating flow, like arterial bifurcations, are marked through an inflammatory phenotype, increased endothelial cell proliferation, apoptosis, enhanced permeability, endothelial cell misalignment, enhanced proinflammatory gene expression, and increased extracellular matrix production (Mohan et al., 1997; Brooks et al., 2004; Cheng et al., 2006; Hahn and Schwartz, 2009). This shows that various hemodynamic patterns can alter endothelial signal transduction and thus protect from or exacerbate vascular disease. Shear stress and flow patterns determine inflammatory signal cascades in endothelial cells. Impaired flow activates an inflammatory phenotype centered on elevated regulation of reactive oxygen species generation, oxidative stress, expression of cytokines, alterations in the ECM, and persistent alterations in inflammatory gene expression from hours to days, which can even be seen in atherosclerosis-resistant animal models (Thoumine et al., 1995; Orr et al., 2005; Jongstra-Bilen et al., 2006; Hahn and Schwartz, 2009). These alterations based on perturbed flow led to a permanently activated inflammatory phenotype, sustained inflammation, and reorganization of the blood vessels. In opposition, laminar flow stimulates only transient endothelial inflammation symptoms that decay fast (Hsieh et al., 1998).

### 5.2 Hypertension governs signal transduction processes and mechanosensors of endothelial cells

**FIGURE 7:**
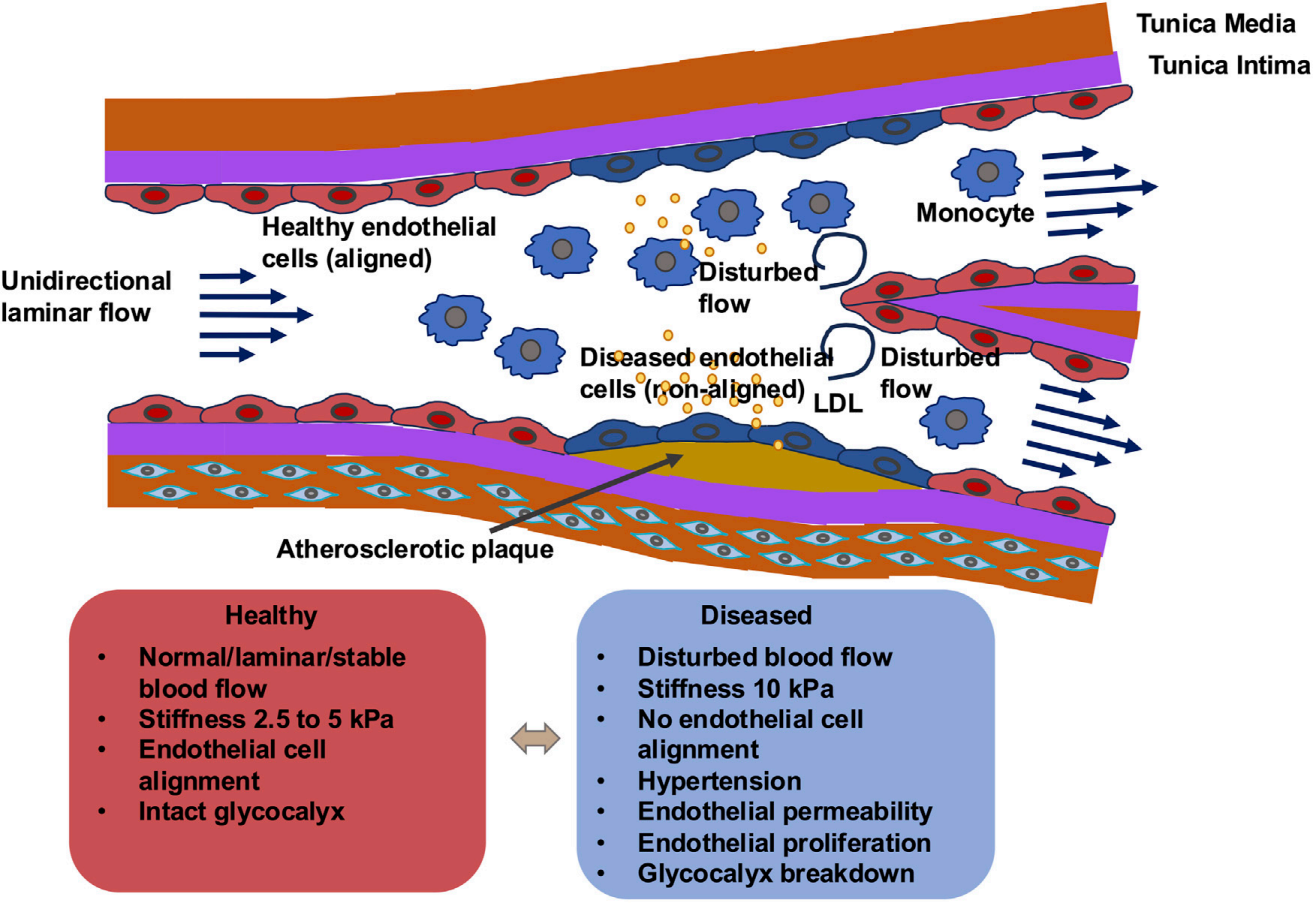
Development of diseases such as atherosclerosis: the behavior of endothelial cells is guided by dynamics of the vascular microenvironment. Most commonly, vessel walls are subject to uniform/laminar/stable flow that fosters the healthy state of endothelial cells. Vessel bifurcations and curvatures can lead to perturbed flow areas, which cause altered endothelial cells with development toward a diseased behavior. For instance, endothelial cells possess hyperpermeability, and they get proinflammatory. Consequently, endothelial cells enable the transmigration of low-density lipoprotein (LDL) and immune cells into the blood vessel wall, whereby atherosclerotic plaques can be built-up.

Chronic hypertension is marked with substantial perturbations of hemodynamic forces (Mayet and Hughes, 2003), to which endothelial cells are exposed and which probably affect mechanosensation. Hypertension leads to oxidative stress elevation, the formation of reactive oxygen species, and inflammation. Blood flow disorders are further risks for the development of atherosclerotic plaques (Figure 7) (Cheng C. et al., 2003; Jongstra-Bilen et al., 2006; Hahn and Schwartz, 2009). It is well known that hypertension is linked to impaired expression and/or dysfunction of mechanosensors and mechanotransducers. Reactive oxygen species produced in hypertension react with NO and remove it, thus preventing vasodilation (Harrison et al., 2006). Animal experiments have also shown that the activation of mechanosensitive channels is compromised because of disease. Whether the changed activity of a mechanosensor is a reason or a consequence of the altered forces is still uncertain. Hypertension influences the vascular channels, such as TRPV4, Kir2.1, and Piezo1 (Koide et al., 2021). Mutations in genes that encode mechanosensors, such as PIEZO1, are widespread in humans and may be cardiovascular disease risk mechanisms (Ma et al., 2018). Overall, impaired hemodynamics and dysregulated mechanosensors are closely associated with vascular impairment and disease.

### 5.3 Mechanoswitching of endothelial cell phenotypes

Endothelial cells sense changes in their environment, must respond to the neighboring mechanical stimuli, and accommodate to the ongoing alterations in their microenvironment. The cellular adaptation of endothelial cells involves a broad spectrum of responses, including (de)activation, (de)differentiation, and cell proliferation/apoptosis. It is, therefore, helpful for the adaptation of endothelial cells to changes in their environment that they themselves can exhibit plastic behavior. Endothelial cells are capable of significant phenotypic plasticity (Dejana et al., 2017), exemplified by their capacity to change their endothelial phenotype to a mesenchymal phenotype. This plasticity, termed endothelial-to-mesenchymal transition (EndMT), was originally reported in cardiac embryonic development; however, it has also been emphasized in different postnatal pathologies like atherosclerosis, cardiac fibrosis, calcification of the vessels, and pulmonary hypertension (Li et al., 2018a). EndMT seems to be important in these inflammation-related diseases and could be an important connecting element between endothelial dysfunction and inflammation (Cho et al., 2018). In the case of cancer, the EndMT mechanism has been identified in melanoma and pancreatic cancer mouse models (Zeisberg et al., 2007) and is significantly implicated in cancer propagation. Due to their plasticity and their capacity to transdifferentiate into mesenchymal cells, these cells have been characterized through a process referred to as EndMT. This complex process is driven by several different factors that convert endothelial cells into a phenotype marked with mesenchymal protein expression and a mobile, contractile shape. EndMT has originally been characterized in normal heart development, but it is now also found in various pathologies and, in particular, in cancer (Clere et al., 2020). This crossover is linked to a marked reduction in endothelial markers, like VE-cadherin, PECAM-1, Tie-1, Tie-2, and vWF, whereas the expressions of mesenchymal biomarkers, like α-SMA, CD44, COL I/III, FSP-1 (S100A4), SM22a, N-cadherin, and vimentin, rise (Piera-Velazquez and Jimenez, 2019). EndMT markers develop from an early stage with regional reduction of endothelial biomarkers and elevation of some early mesenchymal biomarkers (α-SMA, SM22a, and FSP-1) to a subsequent stage marked by a reduction of endothelial biomarkers and elevation of mesenchymal biomarkers such as matrix proteins, such as fibronectin and COL I, and matrix metalloproteases (MMPs) (Dejana et al., 2017). Originally, EndMT has been viewed as a complete differentiation event; however, in the pathophysiological setting, especially in cancer, it can also be partial. Intermediate levels of differentiation of cancer-derived endothelial cells have been observed (Xiao L. et al., 2015), and these cancer endothelial cells exhibit heterogeneity in their capacity for EndMT. It is in agreement with the phenotypic heterogeneity of endothelial cells within various cancer types (Maishi et al., 2019; Goveia et al., 2020), coupled with the variety of cues emanating from the tumor microenvironment, as EndMT subtuning seems to be regulated by various drivers like TGF-β and basic fibroblast growth factor (bFGF) (Xiao and Dudley, 2017). In addition, partial EndMT has been considered a first step of endothelial sprouting in the course of angiogenesis (Welch-Reardon et al., 2015; Wang S.-H. et al., 2017). The reversibility of EndMT in cancer has hardly been characterized; however, a transition from mesenchyme to the endothelium has recently been implicated in Kaposi’s sarcoma (Li et al., 2018b). Limited *in vitro* research indicates that reversibility of EndMT may arise on exposure to EndMT inducers within a brief period (Rieder et al., 2011), while extended exposure compels mesenchymal differentiated cells to progress to a point where there is no reversion to endothelial characteristics (Xiao L. et al., 2015). Apart from their signal mechanotransduction function of endothelial cells, it is possible that they may undergo a mechanoswitch upon mechanical stimulation, like the epithelial-to-mesenchymal transition of epithelial cells. As expected, EndMT can be induced by mechanical cues via the Alk5-Shc mechanotransduction pathway (Egorova et al., 2011b; Egorova et al., 2011a). Vascular pathologies like atherosclerosis, which are typified by aberrant mechanical forces, are often paralleled by EndMT. Nevertheless, it is not well enough established how the forces impact the mechanotransduction pathways that govern cellular plasticity, inflammation, and, finally, pathology of the vessels. A mechanoreceptor unique to EndMT has been identified, and a molecular Alk5-Shc pathway, resulting in EndMT and atherosclerosis, has been elucidated. Depleting Alk5 abolishes shear stress-induced EndMT reactions, and targeting endothelial Shc genetically decreases EndMT and atherosclerosis in zones of impaired flow. Tensile force and readjustment studies highlight a mechanosensory role for Alk5 in EndMT signaling that appears to be unique and separate from that of other mechanosensors. Despite the multifunctional cytokine TGFβ performing a pivotal function in EndMT (Moustakas and Heldin, 2016; Ma et al., 2020), emerging evidence has indicated that the imposition of atheroporotic (or oscillatory/perturbed) shear stress on endothelial cells induces the expression of EndMT transcription factors and mesenchymal hallmarks *in vitro* and *in vivo* (Saito, 2013; Kovacic et al., 2019). In addition to its embryonic developmental involvement, EndMT has also been linked to inflammatory cardiovascular pathologies (Kovacic et al., 2019), including atherosclerosis (Islam et al., 2021). TGFβ-Alk5 signaling is critical for the reaction to shear stress (Egorova et al., 2011b), and emerging evidence has revealed that endothelial-specific deletion of both TGFβR1 (Alk5) and TGFβR2 retards the outgrowth of atherosclerotic lesions and causes complete remission of well-established plaques, demonstrating a clear cause-and-effect connection of EndMT to atherosclerosis (Frutkin et al., 2009). Finally, Alk5 has been implicated as the receptor in charge of mechano-EndMT, and Shc has been established as a candidate driver of EndMT and atherosclerosis in zones of disrupted shear stress (Liu et al., 2008; Mehta et al., 2021). There is even the possibility for endothelial cells to undergo differentiation into adipocytes via EndMT and into mural cells like pericytes and smooth muscle cells (Huang et al., 2015). Endothelial progenitor cells have also been previously shown to undergo smooth muscle-like progenitor cell differentiation through TGF-β1-driven EndMT (Moonen et al., 2015). In addition, tumor EndMT causes the creation of a tumor microenvironment that is accompanied by cancer-associated fibroblasts (CAFs) and abnormal tumor vessels (Huang et al., 2016; Choi et al., 2018; Choi et al., 2020).

## 6 Conclusion and future directions

There is a need for advanced cell culture systems, such as organoids, to study the effects of shear stress on vessel formation in a sophisticated 3D environment. 3D models are needed instead of 2D monolayer cultures that mimic the structural and mechanical properties of the ECM of tissues as closely as possible. In many studies, magnetic tweezers or several magnetic tweezers were used to mimic unidirectional and bidirectional flows. The question now arises whether there are not much better biophysical systems to simulate the fluid flow through vessels. Alternative ways are needed (interaction of magnetized superparamagnetic beads as they induce a magnetic field, hysteresis). Alternative biophysical methods seem to be needed since the interaction of cells with magnetized beads, as they induce a magnetic field, is given, and an unwanted interaction could falsify the results in this case. Another limitation that needs to be overcome is the type of endothelial cells used as there are very large organ-specific differences that need to be considered when comparing results from different groups and identifying general mechanisms. In addition, the interaction of endothelial cells with immune cells also plays a role regarding their mechanosensing and mechanotransduction properties. The possibility of transition of endothelial cells to mesenchymal cells appears to be of crucial importance in angiogenesis and is likely to play a role in tumor angiogenesis, which requires more research activity in this field. All these new research activities will contribute to a more comprehensive understanding of the function of endothelial cells in mechanosensation and mechanotransduction, which may also have a positive impact on the treatment of diseases, such as cancer, which results in an altered vascular system. Finally, the possibility of personalized medicine for diseases that affect the endothelial cells appears to be given here via special “reprogramming” of the endothelial cells from the patient’s own pool.

The literature analysis outlined in this review article has led to the development and deployment of a human cell-based experimental model that subjects endothelial cells to an ambient environment that includes a properly managed dynamic fluid flow with support that provides mechanical stiffness values corresponding to physiological and pathological states. Based on this experimental approach, the goal is to elucidate how the natural mechanical environment with a mixture of fluid and solid tissue affects the organization of the endothelial glycocalyx, which acts as the foremost sensor of the mechanical surrounding, transferring mechanical force to the endothelial cells and converting the force into intracellular activity. It can be postulated that the endothelial glycocalyx is at its most uninterrupted and at minimum porosity under physiological flow conditions in conjunction with relatively soft subendothelial tissue, whereas it is at its most fragmented and at its thinnest under perturbed (pathological) flow regimes in conjunction with fairly stiff subendothelial tissue. Moreover, it is necessary to investigate the mechanisms through which endothelial glycocalyx translates extracellular forces into the biological response of endothelial cells. The cytoskeleton, such as the spectrin scaffold containing short and stable actin filaments, underneath these mechanosensory structures, comprising caveolae, glycocalyx, and membrane receptors, such as integrins, DDR1, and PIEZO, seem to be key elements that are interrelated. Spectrin is needed for stabilization endothelial caveolae, which controls the regulation of PIEZO1. Glycocalyx constituents, especially HA, can be seen as the primary mechanosensors for fluid shear, and CD44 may be indispensable to transduce cues to the nearby spectrin scaffold. When bound to CD44, spectrins control the density of hyaluronan on the cell surface, sensing shear forces and translating them into changes in plasma membrane tension. In turn, an alteration in the spectrin scaffold could slightly alter the curvature or tension exerted on caveolae, which subsequently activates PIEZO1. Consequently, the calcium influx, probably the mechanical deformation of the caveolae, leads to the release of eNOS from the caveolae and to the activation of eNOS. This dual function of spectrin seems to be essential for the mechanotransduction process in endothelial cells.

It is important, in the future, to investigate the participation of YAP/TAZ as a possible mechanism, whereby the glycocalyx exerts an influence on endothelial cells. In addition, it should also be analyzed whether YAP/TAZ acts upstream of the endothelial glycocalyx and regulates the glycocalyx instead. The research findings were expected to yield evidence on how to regulate mechanotransduction in the cells to control and alter cell performance and how cell-dependent vascular functionality can be fixed or restored. DDR1 has been identified as a mechanosensory receptor. Upon shear stress, DDR1 produces liquid-like biomolecular condensates and co-condenses together with YWHAE, which causes the nuclear translocation of YAP that activates transcription of shear stress-induced genes, such as *ICAM1* and *VCAM1*. Developing *in vitro* models is required to examine the impact of mechanical stress on endothelial cells in a defined microenvironment. Complex models that closely mirror the complex microenvironment *in vivo* can be created using dynamic mechanical loading of endothelial cells.

A better insight into cellular reactions can be gained, and high-throughput testing for potential therapeutic treatments can be carried out (He et al., 2015; Zhang et al., 2017). The emergence of high-throughput screening instruments able to systematically investigate the effects of various mechanical stressors and their associations on endothelial cells would be of great benefit to mechanobiology. This can make it easier to identify key mechanosensors, signaling routes, and pharmaceutical targets (Nazari-Jahantigh et al., 2012; Michalik et al., 2014). The incorporation of multi-omics techniques, comprising proteomics, transcriptomics, epigenomics, and genomics, may enable a comprehensive insight into the molecular alterations that arise in endothelial cells in reaction to mechanical stress. With the help of a multidisciplinary analysis, it is potentially feasible to detect new mechanotransduction processes, possible biomarkers, and therapeutic objectives for cardiovascular illnesses (Wang et al., 2019). Progress in omics techniques and experimental models has contributed to the identification of several new potential therapeutic candidates for atherosclerosis (Tamargo et al., 2023). Omics-based analyses have emerged as a standard procedure to assess alterations in endothelial cells reacting to different flow and disease states. In contrast to conventional reductionist strategies, where only a single or a handful of candidate genes or proteins are investigated at a time, amazing breakthroughs in omics technologies and computational bioinformatics have opened the opportunity to identify alterations in genes, proteins, and metabolites at genome-wide, epigenome-wide, proteome-wide, and metabolome-wide scales, often at single-cell-level resolution and utilizing a limited quantity of the sample. The utilization of these methods with *in vitro* and *in vivo* models has generated a wealth of datasets with flow-based transcriptomic, epigenomic, proteomic, and metabolomic patterns in endothelial cells and blood vessels under both healthy and diseased states (Wu et al., 2007; Chen X. et al., 2010; Ni et al., 2010; Dunn et al., 2014; Goveia et al., 2014; Andueza et al., 2020; Ajami et al., 2017). Initial transcriptomic studies utilized bulk RNA and miRNA probes from pooled endothelial cell cultures and animal tissues to perform microarray and RNA sequencing studies. These investigations revealed many unsuspected flow-responsive genes, miRNAs, and lncRNAs, leading to far-reaching new theories concerning their various functions in endothelial cells and atherosclerosis (Tarbell et al., 2014; Simmons et al., 2016a; Demos et al., 2021). Proteomics investigations with modern mass spectrometry have revealed a large number of flow-sensitive proteins that are expressed differently in endothelial cells as a reaction to the flow or are post-translationally altered (Burghoff and Schrader, 2011; Firasat et al., 2014; Simmons et al., 2016b). Endothelial cell secretome studies indicate that the impaired flow changes the abundance of hundreds of proteins, including ANGPT2 and endothelin 1 (Burghoff and Schrader, 2011). A proteome-wide S-sulfhydration of reactive cysteines (S-sulfhydrome) in endothelial cells in reaction to pro-atherogenic stimuli *in vitro* and *in vivo* revealed hundreds of flow-sensitive S-sulfhydrated proteins (Bibli et al., 2021), such as integrins that are involved in the flow-based vascular relaxation reaction.

Future perspectives of the coupling between mechanosensation and function are still needed for a comprehensive and detailed view of the function of endothelial cells in mechanobiology. Mechanotransduction is frequently assessed after the cell has achieved equilibrium and is depicted as a unidirectional pathway from cytoskeletal activation to the transfer of nuclear events, leading to a displacement of state parameters, such as lineage determination. The mechanotransduction can, nevertheless, also activate feedback circuits that change the cell’s reaction to downstream mechanical impulses. These feedback circuits are essential to keep the cytoskeleton in a reactive equilibrium or to maintain an adapted status. A disturbance of these components impairs the mechanosensory system of the endothelial cells and may form the basis of the pathophysiology. Finally, it can be assumed that future endeavors to investigate the colocalization of mechanosensors will largely profit from new technological progress like super-resolution microscopy.
